# Assessing the Impact of Interdisciplinary Multimodal Pain Treatment on Health‐Related Quality of Life in Chronic Pain: A Systematic Review and Meta‐Analyses

**DOI:** 10.1002/ejp.70176

**Published:** 2025-12-02

**Authors:** Alistair Turvill, Frances Maratos, David Sheffield

**Affiliations:** ^1^ Institute of Education, University of Derby Derby UK; ^2^ School of Psychology University of Derby Derby UK

## Abstract

**Background and Objective:**

Chronic pain lowers health‐related quality of life (HRQoL). This review presents the most complete examination to date of how Interdisciplinary Multimodal Pain Treatment (IMPT) affects domains of HRQoL in adults with chronic pain. We compared IMPT with treatment as usual (TAU) and active control groups (ACGs) at short, intermediate and long‐term follow‐up.

**Databases and Data Treatment:**

We searched EBSCO, Embase, Cochrane, Google Scholar, Web of Science, ASSIA, Dimensions and CORE from inception to December 2024. We included RCTs and quasi‐RCTs of IMPTs reporting HRQoL outcomes. Pre‐ and post‐intervention means were extracted; comparable subscales were combined into amalgamated domains. Random‐effects meta‐analyses of standardised mean differences were conducted. Risk of bias was assessed using Cochrane ROB 1, and evidence quality was rated using GRADE. Sensitivity analyses addressed heterogeneity and study quality.

**Results:**

Forty‐one articles (*N* = 6613) informed 39 meta‐analyses. Sample sizes ranged from *n* = 10 to *n* = 703. IMPTs showed small positive effects versus TAU for Physical Functioning at short and long follow‐up, and for Pain, General Health and Emotional Functioning at all follow‐up points. Exploratory analyses found benefits for Pain versus ACG at short and intermediate follow‐up, Vitality versus TAU and Social Functioning versus both comparators. Heterogeneity was high and evidence quality was very low.

**Conclusion:**

This study adds support and novel evidence regarding the continued use of IMPTs to improve domains of HRQoL in chronic pain. Findings suggest broader benefits than previously reported. Future research should develop methodological designs to improve evidence quality and support deeper insights going forward.

**Significance Statement:**

This is the first review to compare Interprofessional Multimodal Pain Therapy (IMPT) with treatment‐as‐usual (TAU) and active control groups (ACG) across all Health‐Related Quality of Life (HRQoL) domains for chronic pain, with short, intermediate and long‐term follow‐up, adjusting for intervention duration. Findings provide evidence of IMPT's superiority over TAU for physical and emotional functioning, pain and general health. Novel findings show benefits over both TAU and ACG for social functioning, over TAU for vitality and over ACG for pain.

## 1 Introduction

Chronic pain (CP; pain lasting > 3 months (Treede et al. 2019)) is among the most impactful of health issues (Cohen et al. 2021) and treatments provide limited relief (Cashin et al. 2025). CP populations report among the lowest quality of life for patient groups (Hadi et al. 2019). Interdisciplinary Multimodal Pain Treatment programmes (IMPT) are recommended to manage persistent pain (IASP 2024), aiming to improve lived experience and function by developing personal skills, understanding and resilience (Jones et al. 2021). Recommended evaluation of IMPT should include measures of Health‐Related Quality of Life (HRQoL) (Kaiser et al. 2018). However, HRQoL is frequently not measured (50% of IMPT evaluations (Dong et al. 2022)), and popular measures are often misused (e.g., totalling SF—36 scores; Lins and Carvalho 2016). In this article the focus is on the underlying constructs of HRQoL based on Mishra et al. (2012) and is detailed within Section 2.2.1.

Recent reviews have sought to understand the impact of IMPT on HRQoL for CP. A recent NICE (2021) review reported HRQoL improvements in populations of mixed CP but omitted statistical analyses, only examining final rather than change scores, against TAU. Romm et al.'s (2021) meta‐analysis reported small to medium HRQoL improvements on some SF—36 subscales (General Health, Pain, Mental Health, Physical Functioning) but did not make comparisons with controls. A further meta‐analysis examining ‘low‐dosed’ IMPT for chronic low back pain for some subscales reported improvements only in emotional wellbeing at post‐treatment compared to active controls (Hochheim et al. 2023). Overall, while previous reviews have provided some insight, they have had limited scope and lacked completeness. The current work addresses some of these limitations.

The main objective of this review is to determine the effectiveness of IMPT on multiple HRQoL domains for CP. Using amalgamated subscales from different measures allows assessment of discrete HRQoL domains (Mishra et al. 2012) and affords greater statistical power. Analyses compare outcomes at short, intermediate and long‐term Follow‐up, alongside evaluating IMPT of differing duration. Comparison of IMPT will be made against TAU and active control groups (ACG).

### 1.1 Hypotheses

*In line with existing evidence, it is hypothesised that IMPT will offer greater improvements than TAU for the following HRQoL domains: Physical Functioning and Wellbeing (* *H1a* *), Pain (* *H1b* *), General Health (* *H1c* *) and Emotional Functioning and Wellbeing (* *H1d* *). By extrapolation, it is also hypothesised that IMPT will be more effective than TAU or ACG for the remaining subscales (* *H1e* *—H1q); these are exploratory analyses*. *H1a* *–H1q are summarised in Table* *1*, *along with supporting references where they are available*.

*More positive effects will be found comparing IMPT with TAU, than comparing IMPT with ACG* (Marin et al. 2017).

*More positive effects favouring IMPT are expected at short‐term compared to intermediate or long‐term Follow‐up* (Chipchase et al. 2013).

*Interventions of greater duration will be associated with greater positive effects of IMPT compared with TAU or ACG* (Romm et al. 2021).

**TABLE 1:** Overview of hypotheses and established or novel status of health related quality of life outcomes with respect to existing evidence and comparison with treatment as usual and active control groups.

Health related quality of life domains	Interdisciplinary multimodal pain treatment versus treatment as usual	Interdisciplinary multimodal pain treatment versus active control groups
Physical Functioning and Wellbeing	Established (H1a) NICE (2021) and Romm et al. (2021)	Exploratory (H1i)
Pain	Established (H1b) Romm et al. (2021)	Exploratory (H1j)
General Health	Established (H1c) Romm et al. (2021)	Exploratory (H1k)
Emotional Functioning and Wellbeing	Established (H1d) NICE (2021) and Romm et al. (2021)	Exploratory (H1l)
Vitality	Exploratory (H1e)	Exploratory (H1m)
Social Functioning	Exploratory (H1f)	Exploratory (H1n)
Sleep	Exploratory (H1g)	Exploratory (H1o)
Overall HRQoL	Exploratory (H1h)	Exploratory (H1p)

## 2 Methods

### 2.1 Protocol and Registration

This systematic review and meta‐analysis adhered to the Preferred Reporting Items for Systematic reviews and Meta‐Analyses (PRISMA) guidelines. This systematic review was registered on the PROSPERO register prior to commencement (CRD42017072031), and protocol information for the study is available on the PROSPERO project page.

### 2.2 Eligibility Criteria

The current work seeks to evaluate interventions for chronic pain that are multimodal and adhere to a biopsychosocial philosophy. The terminology used to describe these treatments has evolved over time. Labels such as ‘multimodal biopsychosocial intervention’, ‘pain management programme’, ‘interprofessional pain management’ have all been used as descriptions (for an overview of the development in terminology see Gatchel et al. (2014)). The present review adopts the current preferred term—Interdisciplinary Multimodal Pain Treatment (IMPT) (IASP 2025), however, studies that use other terms are admissible for inclusion providing they meet our selection criterion. To be eligible, studies must use a randomised controlled trial (RCT) or quasi‐randomised design, such as a controlled clinical trial (CCT). Studies were included if they involved adults (18 years or older) living with chronic non‐cancer pain (defined as pain lasting 3 months or more), who had been referred to an IMPT. The criterion for IMPT programmes was based on Marin et al. (2017) and had to include (a) a physical component and at least one other element from the biopsychosocial model of pain (social, psychological); (b) include at least two practitioners with different areas of expertise; (c) include a comparator group—either treatment as usual (TAU) (defined as care reflective of the usual management of these participants within the health care system in which the study was conducted.), waitlist control (WLC) (participant receiving TAU whilst waiting to receive the intervention at a later date), or active control group (ACG), defined as interventions designed for the RCT/study in question. Where studies included two IMPTs of differing intensity and no ACG, the lower intensity IMPT was accepted as the comparator group. Although structured team meetings are seen as an important part of IMPT programmes, we expected many studies would not report this explicitly and so chose not to apply this as an inclusion criterion. Studies needed to report a HRQoL outcome; although some advocate a unidimensional approach to HRQoL (Beckie and Hayduk 1997), the current work, like the vast majority of research and practice focuses on a multidimensional approach to HRQoL that assesses specific domains such as physical functioning, emotional wellbeing, vitality and pain (see Bowling (2017) for a review), HRQoL is discussed further in Section 2.2.1. Outcomes had to be measured at two time points: prior to treatment and at a point following completion. Articles published in languages other than English, were excluded. See Table 2 for an overview of the PICO details for this study; further detail about the selection process is located in Appendix S1.

**TABLE 2:** Population, intervention, comparison and outcome (pico) inclusion and exclusion criterion.

	Inclusion criterion	Exclusion criterion
Population	Humans Adults People with diagnosed chronic pain condition	Animals Children and adolescents
Intervention	Interdisciplinary Multimodal Pain Treatment consisting of at least two modalities—Psychological, Sociological, Biological. Can be delivered individually, in a group, or a combination of both.	Monomodal approaches
Comparison	Active control group (ACG) Treatment as usual (TAU) Waiting list control (WLC)	No comparison group
Outcome	Health Related Quality of Life measure (HRQoL)	No Health Related Quality of Life measures
Design Type	Randomised Control Trial (RCT), or Quasi Randomised Control Trial (qRCT) such as a Controlled Clinical Trial (CCT)	Cross Sectional Studies, Case Control Studies, Case Serries, Correlational Studies

#### 2.2.1 Health Related Quality of Life

HRQoL differs from the broader construct of ‘quality of life’ which considers life circumstances beyond health, such as housing, work, finances, relationships and environment (Karimi and Brazier 2016).

Kaiser et al.'s (2018, 677) consensus statement on the evaluation of IMPT recognises HRQoL as one of eight core outcome domains. They define it as:

The functional effect of a medical condition and/or its consequent therapy upon a patient. It is thus subjective and multidimensional, encompassing physical & occupational function, psychological state, social interaction & somatic sensation.

Several scales have been developed to assess HRQoL (see Bowling 2017 for a review). While they are distinct instruments, they share common constructs and conceptualisation, and their subscales often overlap. When constructs align, outcomes from different measures can be combined. This is advantageous as it enables larger sample sizes, and in turn more robust statistical analyses. This also reduces biased interpretations by grouping subscales logically *before* data analysis rather than attempting to retrospectively categorise them.

This approach has been used in cancer research (Mishra et al. 2012) but, to our knowledge, not yet in chronic pain (CP). We apply it here.

### 2.3 Databases and Search Strategy

This review adhered to the PICO strategy (see Table 2). The question posed was: In people living with chronic pain (population), can IMPT (intervention) offer greater improvement compared to TAU or ACG (comparison) in domains of HRQoL (outcome). The following databases were searched until December 2024: EBSCO databases (e.g., CINAHL, PsycINFO, MEDLINE, see Appendix S2 for full listing of databases included in this grouping), Google Scholar, Web of Science, ASSIA, Dimensions and CORE databases. To provide a comprehensive approach, cited references from the items identified for inclusion were also searched for further possible items. Search terms and strings were formulated using Boolean terms ‘AND’ and ‘OR’. Based on the PICO categories, these strategies were tailored to the differing database search functions; full details of all terms, locations and strategies can be found in Appendix S3.

### 2.4 Study Selection

All potential suitable items returned by the search strategies described were initially identified based on title and collected into the reference manager software Zotero. Duplicates were identified and removed, after which a more detailed screening of the abstracts was conducted, resulting in the further removal of ineligible items, Finally, a further screening of the full text items was carried out. At each of these steps a provisional screening of at least 25% of the main sample was carried out by the authors AT and DS independently to ensure consistency and agreement in the application of inclusion and exclusion criteria. The remainder of the sample was processed by AT. Any discrepancies were first discussed between AT and DS, with any unresolved discussions planned to be referred to the third author FM for a deciding judgment; however, discrepancies between AT and DS did not occur.

### 2.5 Data Collection Process and Data Items

Study data was extracted by AT in a systematic manner gathering both study characteristics and main outcomes. Extracted data included: (1) study (author and year of publication), (2) study methods and design, (3) sample information (number, age, sex), (4) intervention group details (intervention duration, programme approach and content), (5) control group details (intervention duration, programme approach and content), (6) study outcomes, (7) experimental intervention results, (8) control intervention results.

In line with earlier work investigating the impact of IMPTs for CP populations (Elbers et al. 2022) the current study will extract, and report key details related to intervention characteristics and delivery. This will include intervention components, group size, delivery team and location, duration, time span, level of tailoring and if there was any follow‐up treatment provided after the end of the intervention. Our approach to categorisation aligns with Elbers et al. (2022); details of the extracted components, delivery team and tailoring are presented here:

Intervention Components:

The reviewers took every session description from each integrated multimodal pain treatment (IMPT) programme and placed it in one of the following groups:

- Education (ed) – knowledge passed from a clinician or other expert to the patient.
- Exercise (ex) – planned physical work such as stretching, pool work or walking.
- Graded activity (ga) – activity plans that rise step‐by‐step on a time schedule or are labelled ‘graded activity.’
- Behavioural therapy (bt) – problem‐solving training, exposure in vivo, rational‐emotive therapy or ACT.
- Relaxation (re) – breathing drills, autogenic training, mindfulness, applied relaxation.
- Self‐management (sm) – sessions on coping with pain, setting life goals or day‐to‐day problem‐solving.
- Pharmacological component (ph) – medicine given for long‐standing pain.
- Workplace advice (wo) – work‐site visits or ergonomic guidance.
- Body‐awareness (ba)—physical‐awareness or psychomotor work that helps patients notice body signals.
- Team meetings (te)—counted only when the patient took part.
- Items that did not match these heads were filed as ‘other’ (oth).

Staff labels:

- Physician (phy)
- Psychologist (psy)
- Physiotherapist (pt)
- Occupational therapist (ot)
- Dietician (die)
- Nurse (nur)
- Social worker (swo)
- Pharmacist (ph)

Tailoring levels:

- Low—personal goals set at the start; every programme reached at least this level.
- Medium—some sessions chosen or offered when they matched a patient's stated need or wish.
- High—length and content of the plan shaped case‐by‐case after individual assessment.

### 2.6 Risk of Bias in Individual Studies

In line with (Higgins et al. 2011), the risk of bias both within individual studies, and for the grouped analysis samples overall, was carried out. An initial sample of *n* = 15 was independently reviewed by all authors. Following discussion and agreement (100%) between all, the remainder of the sample was assessed by AT, with subsequent checking and moderation of this process applied by DS.

Regarding individual studies, the categories of *selection*, *performance*, *detection* and *attrition* bias were evaluated based on information provided by the study authors. Specifically, details regarding randomization, allocation, blinding of participants and personnel, blinding of outcome assessment and management of dropouts and missing data, were sought. Studies that adequately explained how these things were addressed were rated as having a *low* risk of bias for the aspect in question. In cases where details indicated one of the aforementioned areas was not handled in a satisfactory manner, a rating of *high* was given. Where insufficient information was provided to make a judgment, and no further details could be recovered from the corresponding authors, a rating of *unclear* was given for the category in question (a full description of this process is presented in Appendix S4).

In line with the GRADE process (Schünemann et al. 2013), the overall quality of each body of evidence meta‐analysed was also graded as either high, moderate, low or very low. A rating of *high* indicates that there is a high degree of reliability in the data, and it is expected that future studies are unlikely to change the outcome presented here. ‘Moderate’ indicates that there is a reasonable potential for future studies to influence confidence in the effect. *Low* indicates that there is a high likelihood that future studies could impact the confidence in the main effect. *Very low* indicates a very low level of certainty in the current estimates provided. RCTs and CCT started from a rating of *high* and were downgraded by one or two points based on their performance in the domains of study design, risk of bias, inconsistency, indirectness and imprecision. Finally, potential publication bias within each sample was assessed via funnel plots within the analysis (a fuller description of this process is presented in Appendix S5).

### 2.7 Synthesis of Results

**FIGURE 1:**
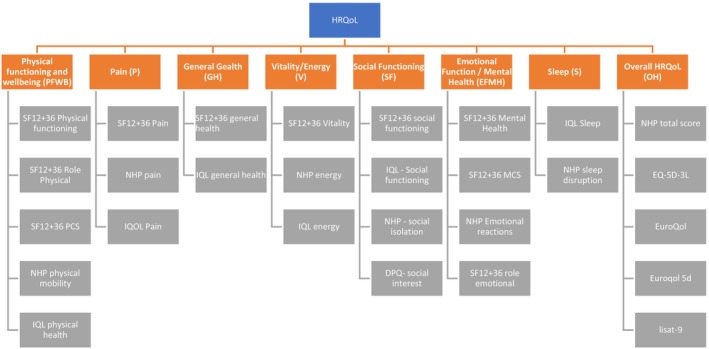
Diagram showing categorisation of subscales from different HRQoL measures into unified subscale constructs.

Data that was extracted included pre–post mean values, Cohen's d and Hedge's g effect sizes, and mean change scores with standard deviation (SD) or confidence interval (CI) values. A key inclusion criterion was for studies to include a specific measure of HRQoL; this could be any of the validated measures with constituent subscales (see Bowling (2017) for a review), or a single item measure. The present review drew upon methods from a 2012 review on HRQoL in cancer treatment (Mishra et al. 2012) to amalgamate directly comparable subscales from different HRQoL measures into a combined outcome. The sample for the present review also included a number of measures not included in Mishra et al.'s (2012) review: Icelandic Quality of Life Scale (Björnsson et al. 1997), Dallas Pain Questionnaire (Lawlis et al. 1989), Nottingham Health Profile (Wiklund 1990). The subscales of these were incorporated into the existing constructs (see Appendix S6). These united constructs, and the various subscales and measures which assessed these, are shown in Figure 1 (see Appendix S6 for full details). In most examples this required the simple combination of Standard Mean Differences (SMDs) and Standard Deviations (SDs) into the analysis. However, in some cases where a single study reported multiple HRQoL subscales which were admissible under the same construct (i.e., the ‘Emotional Wellbeing’ subscale, and ‘Role Emotional’ subscale within the SF‐36), there was a need to avoid potential unit of analysis issues (Higgins et al. 2019). In these cases, the effect sizes for both subscales were calculated and then averaged to create an overall outcome. This average value was then entered into the analysis with the sample size for the study entered once. In addition, potential unit of analysis issues were also identified, as some of the included studies assessed participants at more than one time point. These cases entailed multiple data points from the same treatment group being entered into a single analysis model (i.e., short (0–3 months), intermediate (4–12 months) or long‐term (12+ months) follow‐up). In these cases, the sample size for both groups was divided by two to avoid double counting. Finally, in line with earlier reviews (Marin et al. 2017), where possible the duration of intervention was categorised for each study. Interventions which offered 30 h or less were classified as low duration, those offering 100 h or more were considered high duration, and interventions offering between 31 and 99 h were considered medium duration. In cases where it was not possible to ascertain the number of hours of an intervention, the study was excluded from subgroup analysis.

Where data were missing, corresponding authors were contacted to request additional information. If standard deviations (SDs) were not reported or available, SD values from the baseline for that measure were used instead. There were no instances where neither was reported; all studies reported means or effect sizes.

### 2.8 Quantitative Analyses and Main Outcomes

The software package Meta Essentials was used to analyse the data (Suurmond et al. 2017). All outcomes included in the analyses were continuous. Due to the variation in the samples analysed (i.e., different HRQoL measures, variation of intervention specifics, varied pain populations, different types of practitioners delivering the intervention), Standard Mean Differences (SMDs) and Random Effects Models were chosen as the most appropriate analytical approach (Deeks et al. 2023). Uncertainty for findings was expressed in the form of 95% confidence intervals. Evaluation of effect sizes followed Cohen's suggestions of 0.2 be considered a small effect, 0.5 a moderate effect and 0.8 a large effect (Cohen 1988). Main outcomes for the current meta‐analyses included the eight domains of HRQoL as presented in Figure 1. Comparisons for each of these domains were made at short, intermediate and long‐term follow‐up. For each timeframe, separate analyses were conducted for comparisons between IMPT and TAU/WLC and IMPT and ACGs, resulting in up to potentially 48 different findings (i.e., 8 HRQoL Domains ×3 Timepoints—short, intermediate, long—×2 Comparator Groups—TAU & ACG).

### 2.9 Sensitivity Analysis

Sensitivity analyses were applied in two circumstances:

#### 2.9.1 Analysis Excluding Outliers (AEO)

When the main outcome reported an *I* ^2^ value above 60% (Deeks et al. 2023)—or when the *I* ^2^ was below 60% but the Chi‐square test was statistically significant, outliers were identified and removed in a stepwise fashion. This was done to reduce heterogeneity and bring it within the acceptable range.

#### 2.9.2 Analysis of Higher Quality Studies Only (AHQ)

When there were items in the sample for the main analysis that were rated as ‘*low*’ for the GRADE categories ‘*randomization*’ and ‘*allocation bias*’, these items were excluded from the sample and the analysis was re‐run including only the remaining sources.

In cases where both circumstances were found, both sensitivity analyses were applied independently. In cases where neither of these criteria was met, no sensitivity analysis was applied.

### 2.10 Clinical Relevance

Approaches to determining minimal important differences (MIDs) in HRQoL vary widely, with guidelines recommending a combination of anchored and distribution‐based methods (Mouelhi et al. 2020). Anchored methods use comparisons with simultaneous measures as benchmarks. However, existing MIDs are usually too specific to transfer between contexts (Jayadevappa et al. 2017) and relying on established MIDs alone can underestimate meaningful patient benefits (Schünemann et al. 2023).

Due to methodological limitations in the current study—such as a lack of suitable anchor measures and reliance on combined standardised mean differences (SMD)—a purely distributional approach is adopted. Cohen's (1988) effect size thresholds (small = 0.2, medium = 0.5, large = 0.8) guide the assessment of clinical relevance. Given the complexity and treatment resistance of chronic pain, even small positive effects may be clinically relevant.

The determination of clinical relevance categorisation is reached via the following process (see Table 3):

1. Step 1—Main analysis outcomes are examined, and if, A: SMD > 0.2, B: CIs do not cross 0 and C: heterogeneity estimates are within range (*I* ^2^ < 60%, Chi Sq. < *p* = 0.05)—an outcome is categorised as ‘probably clinically relevant’. If any of these criteria are not met, the clinical relevance categorisation will be determined by step 2.
2. Step 2—If application of either of the two sensitivity analyses processes (AEO, AHQ) results in all criterion (SMD, CI and Heterogeneity) now meet the thresholds presented in step 1, the outcome will be evaluated as ‘maybe’ clinically relevant.

n.b. If trim and fill analysis indicates potential publication bias and reports an adjusted model, this outcome must meet also meet criterion A–C. Further details regarding the clinical relevance assessments can be found in Appendix S7.

**TABLE 3:** Overview of process to determine clinical outcomes categorisation.

	Consider	Criteria A	Criteria B	Criteria C	Criteria D	Outcome	Decision
Step 1	Does the Main Effect meet criteria A–D	SMD ≥ 0.2	95% CIs don't cross 0	*I* ^2^ ≤ 60% and Chi Sq. ≤ *p* = 0.05	If ‘trim and fill’ reports adjusted model it meets A–C (only when *N* ≥ 10)	If yes→	‘Probably Clinically Relevant’
						If no→	Proceed to step 2
Step 2	Do either AEO or AHQ sensitivity analyses meet criteria A–D	SMD ≥ 0.2	95% CIs don't cross 0	*I* ^2^ ≤ 60% and Chi Sq. ≤ *p* = 0.05	If ‘trim and fill’ reports adjusted model it meets A–C (only when *N* ≥ 10)	If yes→	‘Maybe Clinically Relevant’
						If no→	‘Probably Not Clinically Relevant’

Abbreviations: AEO, Analysis Excluding Outliers; AHQ, Analysis of higher quality studies only.

## 3 Results

### 3.1 Sample Overview

**FIGURE 2:**
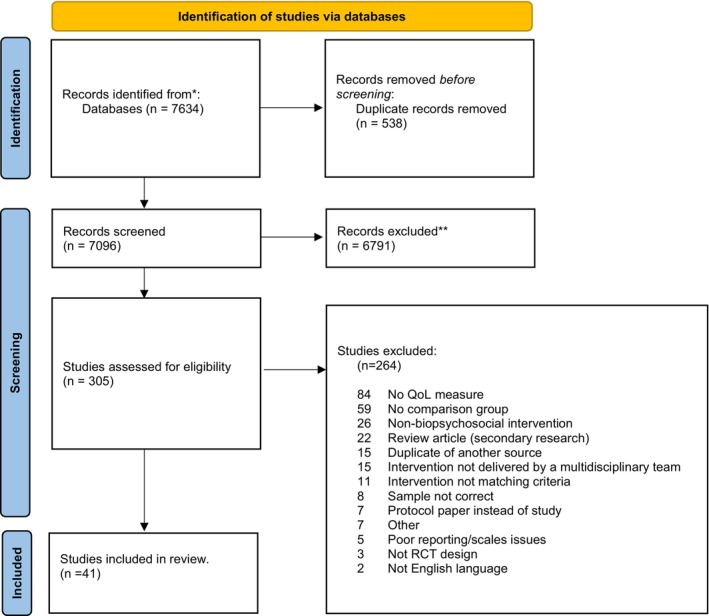
PRISMA flow diagram of CP article inclusion in the systematic reviewer and meta‐analysis.

A total of 7634 references were initially retrieved from the search. After removing duplicates and non‐relevant records, 41 studies were retained (see Figure 2). Data extraction for these studies is summarised in Table 4 and presented in full in Appendix S8. The studies focused on people with CP who were referred for a multimodal biopsychosocial intervention. Pain conditions included chronic widespread pain (*n* = 2), non‐specific chronic pain (*n* = 12), non‐specific chronic low back pain (*n* = 14), non‐specific chronic back pain (*n* = 1), chronic knee pain (*n* = 3), fibromyalgia (*n* = 7), chronic pelvic pain (*n* = 1) and chronic spinal pain (*n* = 1). Sample sizes ranged from 10 to 703, and study designs consisted of randomised controlled trials, quasi‐randomised controlled trials, or controlled clinical trials. HRQoL was evaluated using various self‐report measures, most of which included multiple subscales (see Table 4 and Appendix S9).

**TABLE 4:** Characteristics of studies included in the CP meta‐analysis of HRQoL.

Source	Pain category	Baseline sample size	Sex balance	Comparison	Location	Measure
Amris et al. (2014)	Chronic widespread pain	*n* = 191	100% female	Medium Duration IMPT versus WLC	Denmark	SF36—PCS, MCS, PF
Angeles et al. (2013)	Non‐specific Chronic Pain	*n* = 63	63% female	Low Duration IMPT versus WLC	Canada	SF36—PCS, MCS, PF, BP, GH, RE, V MH, SF, RP
Angst et al. (2009)	Non‐specific chronic back pain	*n* = 331	73% female	Medium duration IMPT versus High duration IMPT	Switzerland	SF36—PCS, MCS, PF, BP, GH, RE, V MH, SF, RP
Becker et al. (2000)	Chronic widespread pain	*n* = 167	65% female	Medium Duration IMPT versus WLC with TAU versus TAU plus single consultation with GP and Pain specialist	Denmark	SF36—PF, BP, GH, RE, V MH, SF, RP
Björnsdóttir et al. (2016)	Non‐specific Chronic Pain	*n* = 234	100% female	High duration IMPT versus high duration ACG versus WLC	Iceland	Icelandic Quality of Life (IQL) (GH, SF, E, PH, P, S, QoL)
Blake et al. (2016)	Non‐specific Chronic Pain	*n* = 46	63% female	Medium Duration IMPT versus WLC	Ireland	SF36—PCS, MCS
Bourgault et al. (2015)	Fibromyalgia	*n* = 56	93% female	Low duration IMPT versus WLC	Canada	SF12—PCS, MCS
Cedraschi et al. (2004)	Fibromyalgia	*n* = 164	76% female	Low duration IMPT versus WLC	Switzerland	SF36—PF, RP, GH, SF
Dufour et al. (2010)	Non‐specific chronic low back pain	*n* = 286	56% female	Medium duration IMPT versus Low duration ACG	Denmark	SF36—PCS, MCS, PF, BP, GH, RE, V MH, SF, RP
Dysvik et al. (2010)	Non‐specific Chronic Pain	*n* = 117	79% female	Medium Duration IMPT versus WLC	Norway	SF36—PCS, MCS, PF, BP, GH, RE, V MH, SF, RP
Gatchel et al. (2009)	Non‐specific Chronic Pain	*n* = 66	33% female	Unclear duration IMPT versus Unclear duration ACG	USA	SF36—PCS, MCS
Grahn et al. (1998)	Non‐specific Chronic Pain	*n* = 236	82% female	High duration IMPT versus TAU	Sweden	Nottingham Health Profile (NHP)—E, P, ER, SI, S, PM, T
Grahn et al. (2000)	Non‐specific Chronic Pain	*n* = 236	82% female	High duration IMPT versus TAU	Sweden	Nottingham Health Profile (NHP)—E, P, ER, SI, S, PM, T
Helminen et al. (2015)	Chronic knee pain	*n* = 111	70% female	Low Duration IMPT versus TAU	Finland	SF36—PF, BP, GH, RE, V MH, SF, RP
Hurley et al. (2007)	Chronic knee pain	*n* = 418	77% female	Low Duration IMPT versus TAU	England	EQ‐5D‐3L
Jensen et al. (2001)	Spinal Pain	*n* = 214	55% female	High duration IMPT versus High duration ACG versus TAU	Sweden	SF36—PF, BP, GH, RE, V MH, SF, RP
Jensen et al. (2011)	Non‐specific chronic low back pain	*n* = 351	52% female	Unclear duration IMPT versus TAU	Denmark	SF36—PF, BP, GH, RE, V MH, SF, RP
Jousset et al. (2004)	Non‐specific chronic low back pain	*n* = 82	34% female	High duration IMPT versus Low duration ACG	France	Dallas Pain Questionnaire (DPQ)—SI
Kwok et al. (2016)	Chronic knee pain	*n* = 46	Unclear	Low Duration IMPT versus WLC	Hong Kong	SF 36—PCS, MCS, PF, GH, RE, V MH, SF, RP
Lang et al. (2003)	Non‐specific chronic low back pain	*n* = 208	58% female	High duration IMPT versus TAU	Germany	SF36—PCS, MCS, PF, BP, GH, RE, V MH, SF, RP
Lera et al. (2009)	Fibromyalgia	*n* = 83	100% female	Medium Duration IMPT versus Medium ACG	Spain	SF36—PCS, MCS
Luciano et al. (2013)	Fibromyalgia	*n* = 216	97% female	Low Duration IMPT versus TAU	Spain	EQ‐5D‐3L
Martins et al. (2014)	Fibromyalgia	*n* = 27	59% female	Low Duration IMPT versus TAU	Brazil	SF36—PCS, MCS
Monticone et al. (2012)	Non‐specific chronic low back pain	*n* = 80	75% female	Low Duration IMPT versus Low duration ACG	Italy	SF36—PF, BP, GH, RE, V MH, SF, RP
Monticone et al. (2013)	Non‐specific chronic low back pain	*n* = 90	58% female	Low Duration IMPT versus Low duration ACG	Italy	SF36—PF, BP, GH, RE, V MH, SF, RP
Monticone et al. (2014)	Non‐specific chronic low back pain	*n* = 10	55% female	Low Duration IMPT versus Low duration ACG	Italy	SF36—PF, BP, GH, RE, V MH, SF, RP
Morone et al. (2011)	Non‐specific Chronic Pain	*n* = 70	64% female	Low Duration IMPT versus TAU	Italy	SF36—PCS, MCS
Nøst et al. (2018)	Chronic pelvic pain	*n* = 121	87% female	Low Duration IMPT versus Low duration ACG	Norway	EQ‐5D‐5L
Nygaard et al. (2020)	Non‐specific chronic low back pain	*n* = 62	100% female	High duration IMPT versus High duration ACG	Norway	EQ‐5D‐5L
Paolucci et al. (2017)	Non‐specific chronic low back pain	*n* = 53	82% female	Low Duration IMPT versus Low duration ACG	Italy	SF36—PF, BP, GH, RE, V MH, SF, RP
Roche‐Leboucher et al. (2011)	Non‐specific chronic low back pain	*n* = 132	35% female	High duration IMPT versus Low duration ACG	France	Dallas Pain Questionnaire (DPQ)—SI
Ronzi et al. (2017)	Non‐specific chronic low back pain	*n* = 159	40% female	High duration IMPT versus Med duration IMPT versus Low duration ACG	France	SF36—PCS, MCS
Rooks (2007)	Fibromyalgia	*n* = 207	100% female	High duration IMPT versus Med duration ACG versus Low duration IMPT	USA	SF36—PF, BP, GH, RE, V MH, SF, RP
Saral et al. (2016)	Fibromyalgia	*n* = 66	100% female	Medium Duration IMPT versus Low duration IMPT versus WLC	Turkey	SF36—PCS, MCS, PF, BP, GH, RE, V MH, SF, RP
Tavafian et al. (2008)	Non‐specific chronic low back pain	*n* = 102	100% female	Low Duration IMPT versus Low duration ACG	Iran	SF36—PCS, MCS
Tavafian et al. (2011)	Non‐specific chronic low back pain	*n* = 197	78% female	Low Duration IMPT versus TAU	Iran	SF36—PF, BP, GH, RE, V MH, SF, RP
Tavafian et al. (2017)	Non‐specific chronic low back pain	*n* = 197	78% female	Low Duration IMPT versus TAU	Iran	SF36—PF, BP, GH, RE, V MH, SF, RP
Taylor et al. (2016)	Non‐specific Chronic Pain	*n* = 703	67% Female	Low Duration IMPT versus TAU	England	EQ‐5D‐3L
van der Hulst et al. (2008)	Non‐specific chronic low back pain	*n* = 163	39% female	Medium duration IMPT versus TAU	Holland	SF36—PCS, MCS
Van der Maas et al. (2016)	Non‐specific Chronic Pain	*n* = 94	81% female	High duration IMPT versus Med duration IMPT.	Holland	SF36—PCS, MCS
Westman et al. (2010)	Non‐specific Chronic Pain	*n* = 158	70% female	Medium duration IMPT versus TAU	Sweden	SF36—PCS, MCS

Abbreviations: ACG, active control group; DPQ—SI, Social Interaction; EQ‐5D‐3L, EuroQoL‐5‐3‐level scale; EQ‐5D‐5L, EuroQoL‐5‐5‐level scale; HP—ER, emotional reactions; IMPT, Interdisciplinary Multimodal Pain Treatment; IQL—E, Energy; IQL—GH, General health; IQL—P, Pain; IQL—PH, Physical health; IQL—QoL, Quality of life; IQL—S, Sleep; IQL—SF, Social functioning; NHP—E, energy; NHP—P, Pain; NHP—PM, Physical Mobility; NHP—S, Sleep disruption; NHP—SI, Social Isolation; NHP—T, Total HRQoL; SF36—BP, bodily pain; SF36—GH, general health; SF36—MCS, mental component summary; SF36—MH, mental health; SF36—PCS, physical component summary; SF36—PF, physical functioning; SF36—RE, role functioning emotional; SF36—RP, role functioning physical; SF36—SF, social functioning; SF36—V, vitality; TAU, treatment as usual; WLC, waiting list control.

#### 3.1.1 Description of Intervention Characteristics

Across the 41 cohorts, 37 (90%) programmes were delivered on an outpatient basis. 21 were exclusively delivered in group settings, with 16 combining group and individual sessions, three were delivered one‐to‐one and one study did not specify the format. Mean group size was 8 (range 1–22). Interventions ran for between 1 and 46 weeks with a mean duration of 7 weeks. Total hours of delivery ranged between 10 h and 150 h with a median of 28.5 h and mean of 48 h. 16 programmes ran in hospitals, 13 in specialised rehabilitation clinics, and the remainder being distributed across community health centres, medical centres, pain clinics, a university facility and a single primary‐care setting. Intervention content was varied between programmes: Education (98%) and physical exercise (95%) appeared in almost every cohort, while self‐management training (78%), behavioural components (73%) and relaxation techniques (66%) were also common. Less frequent elements included body‐awareness work (29%), graded activity (20%), workplace advice (20%), pharmacological input (20%) and structured team meetings with the patient present (2%). Modalities coded as ‘other’ were varied, with examples including vibration‐massage (Björnsdóttir et al. 2016), and balneotherapy (Jousset et al. 2004). Each cohort combined a median of five distinct treatment modalities (range = 2–9). Delivery teams were varied in their constituent make‐up. Physicians participated in 93% of teams and physiotherapists in 88%. Psychologists joined 71%, occupational therapists 39% and nurses 27% of cohorts, with social workers listed in 17%. Tailoring was described as low in 19 cohorts, medium in 14 and high in eight. Structured follow‐up after the main programme (most often refresher group sessions or telephone calls) was reported in 13 cohorts; four of these included an extended module that continued elements of the programme for several weeks or months. Further details of the interventions for each included study are presented in Table 5.

**TABLE 5:** Overview of treatment modalities, healthcare professionals, intervention details including duration and degree of tailoring.

Article	Out/In patient	Group/individual	Treatment modalities	Healthcare providers	Group size	Location	Time span (weeks)	Duration	Level of tailoring	Follow up
Amris et al. (2014)	Out	Group	ed, ex, ga, re, sm, bt, oth	phy, psy, nur, ot, pt	8	Hospital	2 weeks	35 h	Med	Yes
Angeles et al. (2013)	Out	Group	ed, re, bt, ex, sm	ot, swo, phy, ph, pt., die	NA	Health Centre	2 weeks	16 h	Low	No
Angst et al. (2009)	In	Mixed	ba, bt, ed., ex, ga, re, sm, ph, oth	nur, phy, pt., psy, ot	6	Rehabilitation Clinic	4 weeks	100+ hours	Med	Yes
Becker et al. (2000)	Out	Individual	bt, ed., re, ph, oth, ex	phy, psy, pt., nur, swo	1	Pain Clinic	46 weeks	NA	High	No
Björnsdóttir et al. (2016)	In	Mixed	ex, ba, ed., bt, re, ph, sm, oth	pt, psy, nur, phy	NA	Rehabilitation Clinic	4 weeks	101 h	Med	No
Blake et al. (2016)	Out	Mixed	bt, ed., ex, ga, re, sm	phy, ot, pt., psy	6–8	Hospital	4 weeks	72 h	Low	No
Bourgault et al. (2015)	Out	Group	ed, bt, ex, re, sm	psy, pt	8	University Facility	11 weeks	22.5 h	Med	Yes
Cedraschi et al. (2004)	Out	Group	ex, re, ed., sm	pt, ot, phy, psy	8–10	Hospital	6 weeks	18 h	Low	No
Dufour et al. (2010)	Out	Mixed	ex, ed	pt, ot	6	Hospital	12 weeks	95 h	Med	No
Dysvik et al. (2010)	Out	Group	bt, ed., ex, re, sm	nur, pt., psy, phy	10–11	Hospital	8 weeks	40 h	Low	No
Gatchel et al. (2009)	Out	NA	ex, bt, sm	nur, phy, psy, pt., ot	NA	Medical Centre	3 weeks	NA	High	No
Grahn et al. (1998)	In	Mixed	ba, bt, ed., ex, re, sm, wo	phy, pt., ot, swo, nur	NA	Rehabilitation Clinic	4 weeks	NA	High	Yes
Grahn et al. (2000)	In	Mixed	ba, bt, ed., ex, re, sm, wo	phy, pt., ot, swo, nur	NA	Rehabilitation Clinic	4 weeks	NA	High	Yes
Helminen et al. (2015)	Out	Mixed	bt, ed., re, ex, sm	psy, pt	7–13	Medical Centre	6 weeks	12 h	Low	No
Hurley et al. (2007)	Out	Mixed	ex, ed., sm	phy, pt	8	Hospital	6 weeks	12 h	Med	No
Jensen et al. (2001)	Out	Group	ex, re, ba, bt, ed., sm, wo	phy, pt., psy	4–8	Rehabilitation Clinic	4 weeks	135 h	Med	Yes
Jensen et al. (2011)	Out	Individual	ed, ex, ph, sm, wo	phy, pt., swo, ot	1	Hospital	18 weeks	NA	High	No
Jousset et al. (2004)	Out	Group	ex, bt, ed., ga, te, oth	pt, phy, psy, ot	NA	Rehabilitation Clinic	5 weeks	150 h	Medium	No
Kwok et al. (2016)	Out	Mixed	ed, ex, re, sm	phy, nur	6–7	Health Centre	6 weeks	12 h	Low	No
Lang et al. (2003)	Out	Group	ex, bt, re, ed	psy, pt., phy	7–12	Health Centre	7 weeks	85 h	Med	No
Lera et al. (2009)	Out	Group	bt, ed., ex, ph, re, sm	phy, pt., psy	20–22	Hospital	15 weeks	36.5 h	Low	No
Luciano et al. (2013)	Out	Group	ed, re	phy, psy	18	Medical Centre	9 weeks	18 h	Low	No
Martins et al. (2014)	Out	Group	ed, bt, ex, ba, re, sm, oth	phy, ot, pt., psy, swo	12	Pain Clinic	12 weeks	12 h	Low	No
Monticone et al. (2012)	Out	Individual	ex, ed., bt, ga, sm	pt, phy, psy	1	Hospital	8–12 weeks	10 h	High	No
Monticone et al. (2013)	Out	Mixed	bt, ex, ed., sm, ga	phy, psy, pt	1	Rehabilitation Clinic	5 weeks	27 h	Low	Yes
Monticone et al. (2014)	Out	Mixed	ex, bt, ed., sm, ga	phy, psy, ot, pt	1	Hospital	8 weeks	24 h	High	No
Morone et al. (2011)	Out	Group	ba, ed., ex, sm, wo	phy, pt	4–5	Rehabilitation Clinic	4 weeks	10 h	Low	No
Nøst et al. (2018)	Out	Group	ba, bt, ed., ex, re, sm	phy, pt	7–13	Health Centre	6 weeks	15 h	Low	No
Nygaard et al. (2020)	Out	Group	ba, bt, ed	pt, nur, phy	NA	Hospital	2 weeks	NA	Low	Yes
Paolucci et al. (2017)	Out	Mixed	ba, ed., ex, sm	phy, pt	4–5	Hospital	5 weeks	10 h	Low	No
Roche‐Leboucher et al. (2011)	Out	Mixed	ex, re, ed., wo, bt	ot, phy, psy, pt	6–8	Rehabilitation Clinic	5 weeks	150 h	Med	No
Ronzi et al. (2017)	Out	Mixed	ex, ed., re, bt	phy, psy, pt., nur	6–8	Rehabilitation Clinic	5 weeks	150 h	Med	No
Rooks (2007)	Out	Group	ex, ed., sm	phy, pt	NA	Hospital	16 weeks	46 h	Low	No
Saral et al. (2016)	Out	Mixed	bt, ed., ex, re, sm	phy, psy	22	Hospital	10 weeks	45 h	Low	No
Tavafian et al. (2008)	Out	Group	ed, ex, re, sm, bt, ph	psy, phy, pt	NA	Hospital	1 week	30 h	Med	Yes
Tavafian et al. (2011)	Out	Group	ed, ex, re, bt, sm, ph	pt, phy, psy	NA	Hospital	1 week	10 h	Low	Yes
Tavafian et al. (2017)	Out	Group	ed, ex, sm, ph	phy, psy	NA	Hospital	1 week	10 h	Low	Yes
Taylor et al. (2016)	Out	Group	bt, ed., ex, re, sm	phy, psy, pt	14	Health Centre	3 weeks	14 h	Low	Yes
van der Hulst et al. (2008)	Out	Mixed	ed, ex, wo, sm	phy, pt., ot, psy	8	Rehabilitation Clinic	7 weeks	63 h	Med	Yes
Van der Maas et al. (2016)	Out	Group	ba, bt, ed., ex, ga, re, oth	phy, psy, ot	NA	Rehabilitation Clinic	12 Weeks	109 h	Med	No
Westman et al. (2010)	Out	Mixed	ba, bt, ed., ex, re, sm, wo, oth	phy, pt., psy, swo	6–8	Health Centre	6 weeks	96 h	High	No

*Note:* The level of tailoring (Tailoring was classed as Low when care started with shared goal‐setting, Medium when some modules were added or omitted for patient needs, and High when schedule and content were planned Individually after assessment.) NA—Information not available from journal or authors.

Abbreviations: ba, body‐awareness; bt, behavioural therapy; die, dietician; ed., education; ex, exercise; ga, graded activity; nur, nurse; ot, occupational therapist; oth, other; ph, pharmacist; ph, pharmacological component; phy, physician; psy, psychologist; pt. = physiotherapist; re, relaxation; sm, self‐management; swo, social worker; te, team meetings; wo, workplace advice.

#### 3.1.2 Description of TAU and WLC Conditions as Described Within the Sample

23 studies used a waiting‐list or usual‐care arm. In nine studies the control group stayed on a list while any current medication or appointments carried on (Amris et al. 2014; Bourgault et al. 2015; Cedraschi et al. 2004). Several reported relying on routine general‐practice management: Becker et al. (2000), Hurley et al. (2007) and Helminen et al. (2015) let doctors adjust drugs or make referrals as required, and Westman et al. (2010) noted that some controls received physiotherapy or orthopaedic treatment. Lang et al. (2003) provided greater detail, listing drug therapy for most controls, plus injections, physiotherapy, manual therapy, acupuncture or TENS in smaller proportions, while Grahn et al. (1998) described examinations, medicine, stretching advice and home exercises. Jensen et al. (2011) gave a two‐visit assessment with clear reassurance and exercise advice, and Taylor et al. (2016) sent a pain‐education leaflet and relaxation CD, asking patients to practise daily for 3 weeks.

### 3.2 Synthesis of Results

Of the 48 potential analyses within the current review design, sufficient data was recovered to conduct 39 meta‐analyses (there was insufficient data to carry out analyses for IMPT versus ACG ‘overall HRQoL’ at short, intermediate and long‐term follow‐up and insufficient data to carry out any analyses for ‘sleep‐related HRQoL’). As described in Section 2.9 above, where possible, sensitivity analysis aimed at mitigating uncertainty associated with heterogeneity and lower‐quality evidence was applied. For all these outcomes SMD, CIs, *p* and *I* ^2^ values are reported; in cases where *I* ^2^ was < 60% but chi‐square test for heterogeneity was *p* < 0.05, this value is additionally reported in parentheses. Trim and fill analysis was applied to test for asymmetry in the sample. Results for these analyses do not report *p* or *I* ^2^ values but do include Egger testing.

Presented below are narrative descriptions of first the established ([Link], [Link], [Link], [Link]), and then the exploratory analyses which were categorised as *maybe* or *probably* clinically relevant. Data for all outcomes including *probably not* clinically relevant outcomes are presented in Table 6, with an overview of all clinical relevance outcomes also provided in Table 7. Forest plots and details of ROB evaluations for all analyses are presented in supporting information document Data S1.

*Interdisciplinary Multimodal Pain Treatment will offer greater improvements than Treatment as Usual for the Health Related Quality of Life domain: Physical Functioning and Wellbeing* (NICE 2021; Romm et al. 2021).

**TABLE 6:** All results and outcomes from meta‐analyses, presented by construct, comparison group and follow‐up point.

	Follow‐up months	Analysis type	Removed items	Grade evaluation (categories)	Comparative effect. SMD (95% CI); *p*; *I* ^2^; *n*	Clinical relevance (Categories)
*Physical Functioning and Wellbeing*						
IMPT versus TAU	0–3	Main Analysis		⊕⊝⊝⊝ (1, 2, 3)	SMD = 0.41; 95% CI (0.20; 0.62); *p* < 0.001; *I* ^2^ = 62%; *n* = 1383 from 14 studies	Maybe (2)
		Higher Quality Only	Angeles et al. (2013), Becker et al. (2000), Björnsdóttir et al. (2016), Blake et al. (2016), Dysvik et al. (2010), Martins et al. (2014) and Morone et al. (2011)		SMD = 0.46; 95% CI (0.17; 0.76); *p* < 0.001; *I* ^2^ = 52%; *n* = 686 from 7 studies	
		Excluding Outliers	Angeles et al. (2013), Björnsdóttir et al. (2016) and Tavafian et al. (2008)		SMD = 0.33; 95% CI (0.18; 0.48); *p* < 0.001; *I* ^2^ = 4%; *n* = 999 from 10 studies	
	4–11	Main Analysis		⊕⊝⊝⊝ (1, 2, 3)	SMD = 0.36; 95% CI (0.16; 0.56); *p* < 0.001; *I* ^2^ = 61%; *n* = 1480 from 11 studies	Probably not (4)
		Higher Quality Only	Becker et al. (2000), Grahn et al. (1998), Lang et al. (2003), Morone et al. (2011) and Saral et al. (2016)		SMD = 0.35; 95% CI (0.06; 0.63); *p* < 0.001; *I* ^2^ = 64%; *n* = 834 from 6 studies	
		Excluding Outliers	(Saral et al. 2016; Tavafian et al. 2011)		SMD = 0.26; 95% CI (0.09; 0.42); *p* < 0.001; *I* ^2^ = 32%; *n* = 1294 from 9 studies	
		Trim and fill analysis			SMD = 0.14, 95% CI (−0.07; 0.35); (Egger. 11 studies; slope = −0.82; *p* = 0.09)	
	12+	Main Analysis		⊕⊝⊝⊝ (1, 2, 3)	SMD = 0.42; 95% CI (0.03; 0.82); *p* = 0.003; *I* ^2^ = 76%; *n* = 696 from 5 studies	Maybe (2)
		Higher Quality Only	Grahn et al. (2000)		SMD = 0.54; 95% CI (0.18; 0.9); *p* < 0.001; *I* ^2^ = 38%; *n* = 429 from 4 studies	
		Excluding Outliers	Grahn et al. (2000)		SMD = 0.54; 95% CI (0.18; 0.9); *p* < 0.001; *I* ^2^ = 38%; *n* = 429 from 4 studies	
IMPT versus ACGs	0–3	Main Analysis		⊕⊝⊝⊝ (1, 2, 3, 4, 5)	SMD = 0.32; 95% CI (−0.05; 0.7); *p* = 0.05; *I* ^2^ = 78%; *n* = 1190 from 10 studies	Probably not (5)
		Higher Quality Only	Van der Maas et al. (2016)		SMD = 0.55; 95% CI (0.01; 1.10); *p* = 0.01; *I* ^2^ = 76%; *n* = 618 from 7 studies	
		Excluding Outliers	Gatchel et al. (2009) and Monticone et al. (2014)		SMD = 0.15; 95% CI (−0.02; 0.57) *p* = 0.15; *I* ^2^ = 58%; *n* = 1104 from 8 studies	
		Trim and fill analysis			SMD = 0.25, 95% CI (−0.16; 0.65) (Egger. 10 studies. Slope = −2.55, *p* = 0.06)	
	4–11	Main Analysis		⊕⊝⊝⊝ (1, 2, 3, 5)	SMD = 0.33; 95% CI (−0.05; 0.7); *p* = 0.02; *I* ^2^ = 74%; *n* = 1006 from 9 studies	Probably not (5)
		Higher Quality Only	Angst et al. (2009), Becker et al. (2000) and Van der Maas et al. (2016)		SMD = 0.44; 95% CI (−0.14; 1.01); *p* = 0.06; *I* ^2^ = 75%; *n* = 868 from 8 studies	
		Excluding Outliers	(Gatchel et al. 2009; Monticone et al. 2014)		SMD = 0.13; 95% CI (−0.04; 0.31); *p* = 0.06; *I* ^2^ = 18%; *n* = 920 from 7 studies	
		Trim and fill analysis			SMD = 0.23; 95% CI (−0.16; 0.65) (Egger. 9 studies. Slope = −2.59, *p* = 0.02)	
	12+	Main Analysis		⊕⊝⊝⊝ (1, 2, 3, 5)	SMD = 0.34; 95% CI (0.03; 0.65); *p* = 0.01; *I* ^2^ = 75%; *n* = 1061 from 9 studies	Probably not (5)
		Higher Quality Only	Monticone et al. (2012), Van der Maas et al. (2016) and Westman et al. (2010)		SMD = 0.40; 95% CI (−0.05; 0.86); *p* = 0.03; *I* ^2^ = 83%; *n* = 618 from 5 studies	
		Excluding Outliers	Gatchel et al. (2009) and Monticone et al. (2013)		SMD = 0.16; 95% CI (−0.05; 0.38); *p* = 0.08; *I* ^2^ = 50%; *n* = 905 from 7 studies	
*Emotional Functioning and Mental Health*						
IMPT versus TAU	0–3	Main Analysis		⊕⊝⊝⊝ (135)	SMD = 0.36; 95% CI (0.18; 0.54); *p* < 0.001; *I* ^2^ = 30%; *n* = 1048 from 12 studies	Probably (1)
		Higher Quality Only	Angeles et al. (2013), Becker et al. (2000), Blake et al. (2016), Martins et al. (2014) and Morone et al. (2011)		SMD = 0.35; 95% CI (0.11; 0.59); (*p* < 0.001); *I* ^2^ = 30%; *n* = 620 from 6 studies	
		Excluding Outliers			NA—acceptable heterogeneity in the main analysis	
		Trim and fill analysis			SMD = 0.32; 95% CI (0.10; 0.54) (Egger. 12 studies; slope = −0.30; *p* = 0.15)	
	4–11	Main Analysis		⊕⊝⊝⊝ (1, 3)	SMD = 0.29; 95% CI (0.15; 0.42); *p* < 0.001; *I* ^2^ = 5%; *n* = 1305 from 10 studies	Probably (1)
		Higher Quality Only	Becker et al. (2000), Grahn et al. (1998), Lang et al. (2003), Morone et al. (2011) and Saral et al. (2016)		SMD = 0.24; 95% CI (0.04; 0.44); *p* = 0.002; *I* ^2^ = 0%; *n* = 671 from 5 studies	
		Excluding Outliers			NA—acceptable heterogeneity in the main analysis	
	12+	Main Analysis		⊕⊝⊝⊝ (1, 3, 5)	SMD = 0.37; 95% CI (0.06; 0.68); *p* = 0.001; *I* ^2^ = 56% (*p* = 0.041); *n* = 665 from 5 studies	Maybe (3)
		Higher Quality Only	Grahn et al. (2000)		SMD = 0.45; 95% CI (0.08; 0.82); *p* = 0.001; *I* ^2^ = 47%; *n* = 429 from 4 studies	
		Excluding Outliers			No outliers to exclude	
IMPT versus ACGs	0–3	Main Analysis		⊕⊝⊝⊝ (1, 2, 3, 5)	SMD = 0.36; 95% CI (0.02; 0.87); *p* = 0.02; *I* ^2^ = 77%; *n* = 1190 from 10 studies	Probably not (4)
		Higher Quality Only	Angst et al. (2009), Becker et al. (2000), 2; Monticone et al. (2012) and Van der Maas et al. (2016)		SMD = 0.58; 95% CI (0.07; 1.09); *p* = 0.005; *I* ^2^ = 82%; *n* = 618 from 6 studies	
		Excluding Outliers	Monticone et al. (2013)		SMD = 0.22; 95% CI (−0.02; 0.46); *p* = 0.04; *I* ^2^ = 49%; *n* = 1100 from 9 studies	
		Trim and fill analysis			SMD = −0.01; 95% CI (−0.43; 0.40) (Egger. studies = 10. slope = −1.75, *p* = 0.18)	
	4–11	Main Analysis		⊕⊝⊝⊝ (1, 3, 5)	SMD = 0.18; 95% CI (0; 0.36); *p* = 0.03; *I* ^2^ = 26%; *n* = 1006 from 9 studies	Probably not (5)
		Higher Quality Only	Angst et al. (2009), Becker et al. (2000) and Van der Maas et al. (2016)		SMD = 0.25; 95% CI (0; 0.49); *p* = 0.02; *I* ^2^ = 21%; *n* = 611 from 6 studies	
		Excluding Outliers			NA—acceptable heterogeneity in the main analysis	
	12+	Main Analysis		⊕⊝⊝⊝ (1, 2, 3, 5)	SMD = 0.29; 95% CI (−0.18; 0.76); *p* = 0.16; I^2^ = 84%; *n* = 1110 from 8 studies	Probably not (5)
		Higher Quality Only	(Monticone et al. 2012; Van der Maas et al. 2016; Westman et al. 2010)		SMD = 0.43; 95% CI (−0.34; 1.20); *p* = 0.15; *I* ^2^ = 89%; *n* = 825 from 5 studies	
		Excluding Outliers	(Monticone et al. 2013)		SMD = 0.13; 95% CI (0.03; 0.24); *p* = 0.002; *I* ^2^ = 0%; *n* = 1020 from 7 studies	
General Health						
IMPT versus TAU	0–3	Main Analysis		⊕⊝⊝⊝ (1, 3)	SMD = 0.37; 95% CI (0.11; 0.62); *p* < 0.001; *I* ^2^ = 58% (*p* = 0.014); *n* = 955 from 8 studies	Maybe (2)
		Higher Quality Only	Angeles et al. (2013), Becker et al. (2000), Björnsdóttir et al. (2016) and Dysvik et al. (2010)		SMD = 0.23; 95% CI (−0.08; 0.54); *p* = 0.04; *I* ^2^ = 13%; *n* = 401 from 4 studies	
		Excluding Outliers	Björnsdóttir et al. (2016)		SMD = 0.30; 95% CI (0.06; 0.53); *p* = 0.001; *I* ^2^ = 31%; *n* = 686 from 7 studies	
	4–11	Main Analysis		⊕⊝⊝⊝ (1, 3)	SMD = 0.27; 95% CI (0.04; 0.49); *p* = 0.001; *I* ^2^ = 16%; *n* = 716 from 5 studies	Probably (1)
		Higher Quality Only	Becker et al. (2000) and Lang et al. (2003)		SMD = 0.24; 95% CI (0.05; 0.43); *p* < 0.001; *I* ^2^ = 0%; *n* = 416 from 3 studies	
		Excluding Outliers			NA—acceptable heterogeneity in the main analysis	
	12+	Main Analysis		⊕⊕⊕⊝ (1)	SMD = 0.43; 95% CI (0.12; 0.74); *p* < 0.001; *I* ^2^ = 0%; *n* = 252 from 2 studies	Probably (1)
		Higher Quality Only			No low—quality studies to exclude	
		Excluding Outliers			NA—acceptable heterogeneity in the main analysis	
IMPT versus ACGs	0–3	Main Analysis		⊕⊝⊝⊝ (1, 2, 3, 5)	SMD = 0.32; 95% CI (−0.23; 0.96); *p* = 0.18; *I* ^2^ = 87%; *n* = 1030 from 8 studies	Probably not (5)
		Higher Quality Only	Angst et al. (2009), Becker et al. (2000) and Monticone et al. (2012)		SMD = 0.52; 95% CI (−0.36; 1.4); *p* = 0.13; *I* ^2^ = 91%; *n* = 522 from 5 studies	
		Excluding Outliers	Monticone et al. (2013)		SMD = 0.03; 95% CI (−0.18; 0.25); *p* = 0.73; *I* ^2^ = 25%; *n* = 940 from 7 studies	
		Trim and fill analysis			SMD = −0.69; 95% CI (−1.42; 0.04) (Egger. studies 8. Slope = −4.01, *p* = 0.20)	
	4–11	Main Analysis		⊕⊝⊝⊝ (1, 3)	SMD = 0.21; 95% CI (−0.12; 0.55); *p* = 0.12; *I* ^2^ = 55%; *n* = 780 from 6 studies	Probably not (5)
		Higher Quality Only	Angst et al. (2009) and Becker et al. (2000)		SMD = 0.26; 95% CI (−0.36; 0.87); *p* = 0.25; *I* ^2^ = 66%; *n* = 479 from 4 studies	
		Excluding Outliers			NA—acceptable heterogeneity in the main analysis	
	12+	Main Analysis		⊕⊝⊝⊝ (1,2,3,4,5)	SMD = 0.44; 95% CI (−0.3; 1.18); *p* = 0.37; *I* ^2^ = 88%; *n* = 800 from 5 studies	Probably not (5)
		Higher Quality Only	Monticone et al. (2012)		SMD = 0.51; 95% CI (−0.43; 1.46); *p* = 0.13; *I* ^2^ = 90%; *n* = 720 from 4 studies	
		Excluding Outliers	Monticone et al. (2013)		SMD = 0.17; 95% CI (0.03; 0.08); *p* < 0.001; *I* ^2^ = 0%; *n* = 710 from 4 studies	
*Overall HRQoL*						
	0–3	Main Analysis		⊕⊝⊝⊝ (1, 2, 3, 4, 5)	SMD = 0.55; 95% CI (−1.22; 2.21); *p* = 0.08; *I* ^2^ = 58% (*p* = 0.014); *n* = 827 from 3 studies	Probably not (5)
		Higher Quality Only			No low—quality studies to exclude	
		Excluding Outliers			Not possible due to the small sample size	
	4–11	Main Analysis		⊕ ⊕ ⊕⊝ (1, 3)	SMD = 0.11; 95% CI (0.02; 0.19); *p* < 0.001; I^2^ = 0%; *n* = 1155 from 3 studies	Probably not (5)
		Higher Quality Only			No low—quality studies to exclude	
		Excluding Outliers			NA—acceptable heterogeneity in the main analysis	
	12+	Main Analysis		⊕⊝⊝⊝ (1, 2, 3)	SMD = 0.15; 95% CI (−0.24; 0.54); *p* = 0.11; *I* ^2^ = 73%; *n* = 1206 from 4 studies	Probably not (5)
		Higher Quality Only	Grahn et al. (2000) and Luciano et al. (2013)		SMD = −0.03; 95% CI (−0.13; 0.06); *p* < 0.001; *I* ^2^ = 0; *n* = 754 from 2 studies	
		Excluding Outliers	Luciano et al. (2013)		SMD = 0.01; 95% CI (−0.13; 0.06); *p* = 0.81; *I* ^2^ = 0; *n* = 990 from 3 studies	
*Pain*						
IMPT versus TAU	0–3	Main Analysis		⊕⊝⊝⊝ (1, 3)	SMD = 0.38; 95% CI (0.16; 0.60); *p* < 0.001, *I* ^2^ = 31%; *n* = 909 from 7 studies	Probably (1)
		Higher Quality Only	Angeles et al. (2013), Becker et al. (2000), Björnsdóttir et al. (2016) and Dysvik et al. (2010)		SMD = 0.25; 95% CI (−0.08; 0.57); *p* = 0.016; *I* ^2^ = 0%; *n* = 355 from 2 studies	
		Excluding Outliers			NA—acceptable heterogeneity in the main analysis	
	4–11	Main Analysis		⊕⊝⊝⊝ (1, 2, 3)	SMD = 0.44; 95% CI (0.06; 0.81); *p* = 0.001; *I* ^2^ = 69%; *n* = 788 from 5 studies	Maybe (2)
		Higher Quality Only	Becker et al. (2000), Grahn et al. (1998) and Lang et al. (2003)		SMD = 0.52; 95% CI (−0.57; 1.62); *p* = 0.04; *I* ^2^ = 62%; *n* = 253 from 2 studies	
		Excluding Outliers	Becker et al. (2000), Grahn et al. (1998) and Jensen et al. (2001)		SMD = 0.55; 95% CI (0.19; 0.90); *p* < 0.001; *I* ^2^ = 35%; *n* = 354 from 2 studies	
	12+	Main Analysis		⊕⊝⊝⊝ (1,2,3,4)	SMD = 0.53; 95% CI (−0.16; 1.21); *p* = 0.007; *I* ^2^ = 80%; *n* = 488 from 3 studies	Maybe (2) (3)
		Higher Quality Only	Grahn et al. (2000)		SMD = 0.70; 95% CI (0.08; 1.32); *p* < 0.001; *I* ^2^ = 13%; *n* = 252 from 2 studies	
		Excluding Outliers	Grahn et al. (2000)		SMD = 0.70; 95% CI (0.08; 1.32); *p* < 0.001; *I* ^2^ = 13%; *n* = 252 from 2 studies	
IMPT versus ACGs	0–3	Main Analysis		⊕⊝⊝⊝ (1, 2, 3, 5)	SMD = 0.31; 95% CI (−0.12; 0.75); *p* = 0.10, *I* ^2^ = 84%; *n* = 1030 from 8 studies	Probably not (5)
		Higher Quality Only	Angst et al. (2009), Becker et al. (2000) and Monticone et al. (2012)		SMD = 0.49; 95% CI (−0.17; 1.16); *p* = 0.06; *I* ^2^ = 84%; *n* = 552 from 5 studies	
		Excluding Outliers	Monticone et al. (2013)		SMD = 0.11; 95% CI (−0.11; 0.34); *p* = 0.24; *I* ^2^ = 49%; *n* = 940 from 7 studies	
		Trim and fill analysis			SMD = 0.25; 95% CI (−0.83; 0.34); (Egger.8 studies. Slope = −1.74, *p* = 0.43)	
	4–11	Main Analysis		⊕⊝⊝⊝ (1,2,3)	SMD = 0.20; 95% CI (−0.1; 0.49); *p* = 0.10; *I* ^2^ = 56%; *n* = 780 from 6 studies	Maybe (2)
		Higher Quality Only	Angst et al. (2009) and Becker et al. (2000)		SMD = 0.25; 95% CI (−0.02; 0.51); *p* = 0.01; *I* ^2^ = 3%; *n* = 479 from 4 studies	
		Excluding Outliers	Angst et al. (2009)		SMD = 0.25; 95% CI (0.05; 0.45); *p* = 0.001; *I* ^2^ = 0%; *n* = 570 from 5 studies	
	12+	Main Analysis		⊕⊝⊝⊝ (1, 2, 3, 4, 5)	SMD = 0.36, 95% CI (−0.42; 1.13); *p* = 0.24; *I* ^2^ = 91%; *n* = 801 from 5 studies	Maybe (2)
		Higher Quality Only	Monticone et al. (2012)		SMD = 0.49; 95% CI (−0.42; 1.41); *p* = 0.14; *I* ^2^ = 91%; *n* = 721 from 4 studies	
		Excluding Outliers	Jensen et al. (2011) and Monticone et al. (2012, 2013)		SMD = 0.27; 95% CI (0.04; 0.49); *p* < 0.001; *I* ^2^ = 0%; *n* = 389 from 2 studies	
*Social Functioning*						
IMPT versus TAU	0–3	Main Analysis		⊕⊝⊝⊝ (1, 3, 5)	SMD = 0.26; 95% CI (0.02; 0.50); *p* = 0.01; *I* ^2^ = 55% (*p* = 0.021); *n* = 955 from 8 studies	Probably not (5)
		Higher Quality Only	Angeles et al. (2013), Becker et al. (2000), Björnsdóttir et al. (2016) and Dysvik et al. (2010)		SMD = 0.21; 95% CI (−0.13; 0.55); *p* = 0.09; *I* ^2^ = 22%; *n* = 401 from 4 studies	
		Excluding Outliers	Björnsdóttir et al. (2016)		SMD = 0.17; 95% CI (0; 0.33); *p* = 0.02; *I* ^2^ = 0%; *n* = 686 from 7 studies	
		Trim and fill analysis			SMD = 0.02; 95% CI (0.02; 0.5); (Egger.8 studies. Slope = 0.80; *p* = 0.56)	
	4–11	Main Analysis		⊕⊝⊝⊝ (1, 2, 3, 5)	SMD = 0.26; 95% CI (0.11; 0.41); *p* < 0.001; *I* ^2^ = 0%; *n* = 952 from 6 studies	Probably (1)
		Higher Quality Only	Becker et al. (2000), Grahn et al. (1998) and Lang et al. (2003)		SMD = 0.24; 95% CI (0.04; 0.44); *p* < 0.001; *I* ^2^ = 0%; *n* = 416 from 3 studies	
		Excluding Outliers			NA—acceptable heterogeneity in the main analysis	
	12+	Main Analysis		⊕⊝⊝⊝ (1,3,4)	SMD = 0.35; 95% CI (−0.16; 0.86); *p* = 0.03; *I* ^2^ = 56%; *n* = 488 from 3 studies	Probably not (5)
		Higher Quality Only	Grahn et al. (1998)		SMD = 0.47; 95% CI (−0.37; 1.31); *p* = 0.008; *I* ^2^ = 44%; *n* = 252 from 2 studies	
		Excluding Outliers			NA—acceptable heterogeneity in the main analysis	
IMPT versus ACGs	0–3	Main Analysis		⊕⊝⊝⊝ (1,2,3,5)	SMD = 0.27; 95% CI (−0.18; 0.71); *p* = 0.17; *I* ^2^ = 80%; *n* = 1030 from 8 studies	Probably not (5)
		Higher Quality Only	Angst et al. (2009), Becker et al. (2000) and Monticone et al. (2012)		SMD = 0.45; 95% CI (−0.25;1.16); *p* = 0.10; *I* ^2^ = 84%, *n* = 552 from 5 studies	
		Excluding Outliers	Monticone et al. (2013, 2014)		SMD = 0.06; 95% CI (−0.15; 0.26); *p* = 0.50; *I* ^2^ = 36%, *n* = 920 from 6 studies	
		Trim and fill analysis			SMD = 0.17; 95% CI (−0.31; 0.65); (Egger. 8 studies. Slope = −2.38, *p* = 0.16)	
	4–11	Main Analysis		⊕⊝⊝⊝ (1, 2, 3)	SMD = 0.18; 95% CI (−0.27; 0.63); *p* = 0.33; *I* ^2^ = 74%; *n* = 862 from 8 studies	Probably not (5)
		Higher Quality Only	Angst et al. (2009), Becker et al. (2000) and Jousset et al. (2004)		SMD = 0.42; 95% CI (−0.22; 1.05); *p* = 0.07; *I* ^2^ = 65%, *n* = 479 from 4 studies	
		Excluding Outliers	Jousset et al. (2004) and Monticone et al. (2014)		SMD = 0.15; 95% CI (−0.1; 0.4); *p* = 0.12; *I* ^2^ = 39%; *n* = 760 from 5 studies	
	12+	Main Analysis		⊕⊝⊝⊝ (1, 2, 3, 4, 5)	SMD = 0.47; 95% CI (−0.17; 1.11); *p* = 0.04; *I* ^2^ = 88%; *n* = 915 from 6 studies	Maybe (2)
		Higher Quality Only	Monticone et al. (2012)		SMD = 0.55; 95% CI (−0.21; 1.31); *p* = 0.06; *I* ^2^ = 90%, *n* = 835 from 5 studies	
		Excluding Outliers	Monticone et al. (2013)		SMD = 0.22; 95% CI (0.01; 0.43); *p* = 0.008; *I* ^2^ = 20%; *n* = 825 from 5 studies	
Vitality						
IMPT versus TAU	0–3	Main Analysis		⊕⊝⊝⊝ (1, 2, 3)	SMD = 0.44; 95% CI (0.1; 0.77); *p* = 0.001; *I* ^2^ = 81%; *n* = 955 from 8 studies	Maybe (2)
		Higher Quality Only	Angeles et al. (2013), Becker et al. (2000), Björnsdóttir et al. (2016) and Dysvik et al. (2010)		SMD = 0.37; 95% CI (−0.01; 0.63) *p* = 0.006; *I* ^2^ = 17%; *n* = 401 from 4 studies	
		Excluding Outliers	Björnsdóttir et al. (2016)		SMD = 0.30; 95% CI (0.12; 0.48); *p* < 0.001; *I* ^2^ = 0%; *n* = 686 from 7 studies	
	4–11	Main Analysis		⊕⊝⊝⊝ (1, 3)	SMD = 0.37; 95% CI (0.11; 0.62); *p* < 0.001; *I* ^2^ = 35%; *n* = 774 from 5 studies	Probably (1)
		Higher Quality Only	Becker et al. (2000), Grahn et al. (1998) and Lang et al. (2003)		SMD = 0.40; 95% CI (0.09; 0.71); *p* < 0.001; *I* ^2^ 0%; *n* = 252 from 2 studies	
		Excluding Outliers			NA—acceptable heterogeneity in the main analysis	
	12+	Main Analysis		⊕⊝⊝⊝ (1, 3, 5)	SMD = 0.38; 95% CI (−0.01; 0.78); *p* = 0.001; *I* ^2^ = 35%; *n* = 518 from 3 studies	Probably not (5)
		Higher Quality Only	Grahn et al. (2000)		SMD = 0.49; 95% CI (−0.03; 1.02); *p* < 0.001; *I* ^2^ = 0%, *n* = 252 from 2 studies	
		Excluding Outliers			NA—acceptable heterogeneity in the main analysis	
IMPT versus ACGs	0–3	Main Analysis		⊕⊝⊝⊝ (1, 3, 5)	SMD = 0.35; 95% CI (−0.07; 0.77); *p* = 0.06; *I* ^2^ = 76%; *n* = 1030 from 8 studies	Probably not (5)
		Higher Quality Only	Angst et al. (2009), Becker et al. (2000) and Monticone et al. (2012)		SMD = 0.53; 95% CI (−0.15; 1.2); *p* = 0.05; *I* ^2^ = 85%; *n* = 552 from 5 studies	
		Excluding Outliers	Monticone et al. (2013)		SMD = 0.17; 95% CI (0; 0.33); *p* = 0.02; *I* ^2^ = 3%; *n* = 940 from 7 studies	
		Trim and fill analysis			SMD = −0.25 95% CI (−0.81; 0.32); (Egger. 8 studies. Slope = 1.71 *p* = 0.27)	
	4–11	Main Analysis		⊕⊝⊝⊝ (1, 3, 5)	SMD = 0.25; 95% CI (−0.04; 0.55); *p* = 0.04; *I* ^2^ = 44%; *n* = 780 from 6 studies	Probably not (5)
		Higher Quality Only	Angst et al. (2009) and Becker et al. (2000)		SMD = 0.28; 95% CI (−0.19; 0.75); *p* = 0.10; *I* ^2^ = 42%; *n* = 479 from 4 studies	
		Excluding Outliers			NA—acceptable heterogeneity in the main analysis	
		Trim and fill analysis			SMD = 0.19; 95% CI (−0.21; 0.58); (Egger. 6 studies. Slope = −0.65 *p* = 0.05)	
	12+	Main Analysis		⊕⊝⊝⊝ (1, 2, 3, 4, 5)	SMD = 0.44; 95% CI (−0.41; 1.29); *p* = 0.02; *I* ^2^ = 90%; *n* = 800 from 5 studies	Probably not (5)
		Higher Quality Only	Monticone et al. (2012)		SMD = 0.51; 95% CI (−0.59; 1.62); *p* = 0.20; *I* ^2^ = 92%; *n* = 720 from 4 studies	
		Excluding Outliers	Monticone et al. (2013)		SMD = 0.16; 95% CI (0.03; 0.29); *p* = 0.001; *I* ^2^ = 0%; *n* = 710 from 4 studies	

*Note:* Trim fill analyses are only reported in cases where publication bias was indicated for the main analyses. For the Grade Evaluation (categories) Column: ⊕⊝⊝⊝ = Very Low; ⊕⊕⊕⊝ = Moderate. 1 = Study Design, 2 = Risk of Bias, 3 = Inconsistency, 4 = Indirectness, 5 = Impression. Clinical Relevance Categories: 1 = Main outcome at least SMD > 0.2 with good heterogeneity and CIs. 2 = Main outcome SMD < 0.2 and/or invalid for heterogeneity and/or CIs, all factors resolved via analysis excluding outliers. 3 = Main outcome < 0.2 and/or invalid for heterogeneity and/or CIs, all factors resolved via analysis of higher quality studies only. 4 Sample *n* = 10 or greater, trim and fill analysis indicates asymmetry and adjusted model of SMD < 0.2. 5 = main outcome and subsequent sensitivity analyses SMD < 0.2 and have significant problems with heterogeneity and/or CIs (see Appendix S6 for full summary).

**TABLE 7:** Overview of clinical relevance outcomes for all outcomes.

Interdisciplinary multimodal pain treatment versus treatment as usual				
	Short	Intermediate	Long	Hypothesis
Established outcomes				
Physical Functioning and Wellbeing	Maybe	Probably Not	Maybe	H1a
Pain	Probably	Maybe	Maybe	H1b
General Health	Maybe	Probably	Probably	H1c
Emotional Functioning and Wellbeing	Probably	Probably	Maybe	H1d
Exploratory Outcomes				
Vitality	Maybe	Probably	Probably Not	H1e
Social Functioning	Probably Not	Probably	Probably Not	H1f
Sleep	Insufficient data	Insufficient data	Insufficient data	
Overall HRQoL	Probably Not	Probably Not	Probably Not	

Interdisciplinary multimodal pain treatment versus active control groups				
	Short	Intermediate	Long	Hypothesis
Exploratory Outcomes				
Physical Functioning and Wellbeing	Probably Not	Probably Not	Probably Not	
Pain	Probably Not	Maybe	Maybe	
General Health	Probably Not	Probably Not	Probably Not	
Emotional Functioning and Wellbeing	Probably Not	Probably Not	Probably Not	
Vitality	Probably Not	Probably Not	Probably Not	
Social Functioning	Probably Not	Probably Not	Maybe	H1n
Sleep	Insufficient data	Insufficient data	Insufficient data	
Overall HRQoL	Insufficient data	Insufficient data	Insufficient data	

Outcomes in support of H1a were reported for short and long‐term follow‐up. In each case the main analyses *I* ^2^ values exceeded the 60% threshold set (62% and 72%, respectively). Both values were mitigated in subsequent sensitivity analyses. In line with the process described (2.10) this resulted in both outcomes being categorised as ‘maybe clinically relevant’. Intermediate term follow up didn't provide findings in support of IMPT over TAU. In sum, there is support that IMPT outperforms TAU for improving Physical Functioning and Wellbeing in CP populations at short and longer term follow up.

*Interdisciplinary Multimodal Pain Treatment will offer greater improvements than Treatment as Usual for the Health Related Quality of Life domain: Pain* (Romm et al. 2021).

There was a small significant effect in favour of IMPT at short (SMD = 0.38; 95% CI (0.16; 0.60)), intermediate term follow up (SMD = 0.44; 95% CI (0.06; 0.81)) and a medium effect long‐term follow up (SMD = 0.53; 95% CI (−0.16; 1.21)). Heterogeneity estimates for intermediate and long term follow up exceeded *I* ^2^ thresholds (62% and 60%, respectively), sensitivity analyses sufficiently reduced these values in both cases, resulting in these outcomes being categorised as ‘maybe clinically relevant*’*. Heterogeneity estimates in the main analysis for short term follow up were within thresholds and so this outcome was categorised as probably clinically relevant. In sum, there is support for H1b at short, intermediate and long term follow up.

*Interdisciplinary Multimodal Pain Treatment will offer greater improvements than Treatment as Usual for the Health Related Quality of Life domain: General Health* (Romm et al. 2021).

Small significant effects in favour of IMPT were found at short (SMD = 0.37; 95% CI (0.11; 0.62)), intermediate (SMD = 0.27; 95% CI (0.04; 0.49)) and long term follow up (SMD = 0.43; 95% CI (0.12; 0.74)). While *I* ^2^ values for the main analysis in short term follow up were below the 60% threshold, the corresponding chi‐square test was significant, and so, secondary sensitivity analysis excluding significant outliers, and studies of lower‐quality was applied, outcomes support the size and direction of the main effect with no heterogeneity violations. In line with the approach detailed in, this outcome was categorised as ‘Maybe clinically relevant*’*. There were no heterogeneity violations in the main analyses for intermediate, and long‐term follow up and so both were categorised as *probably clinically relevant*’. In sum, support for H1c was found at all follow up points.

*Interdisciplinary Multimodal Pain Treatment will offer greater improvements than Treatment as Usual for the Health Related Quality of Life domain: Emotional Functioning and Wellbeing* (NICE 2021; Romm et al. 2021).

Support for IMPT over TAU to improve Emotional Functioning and Wellbeing as found at all follow up points. For short (SMD = 0.36; 95% CI (0.18; 0.54)) and intermediate term follow up (SMD = 0.29; 95% CI (0.15; 0.42)) there were no issues with heterogeneity estimates, however trim and fill analysis did report an adjusted model for short term follow up (SMD = 0.32; 95% CI (0.10; 0.54)). The size and direction of this supported the main effect, as did all sensitivity analyses for both outcomes. These outcomes were categorised as *maybe*, and *probably clinically relevant* respectively. There was a small significant effect in favour of IMPT for long term follow up (SMD = 0.37; 95% CI (0.06; 0.68)). While *I* ^2^ values for the main analysis were below the 60% threshold the corresponding chi square test was significant, and so, secondary sensitivity analysis excluding significant outliers was attempted. However, there were no outliers within the sample which were appropriate for exclusion. As there was agreement between the main analysis and first sensitivity analysis (and no heterogeneity concerns for this second analysis). This outcome was categorised as *maybe* clinically relevant. Findings provide support for H1d at all points.

#### 3.2.1 Effectiveness of IMPTs on Exploratory Outcomes

Exploratory analyses reporting findings in favour of IMPT (H1e, H1f, H1j, H1n) are presented below. Findings for hypotheses H1g, H1h, H1i, H1k, H1l, H1m, H1o, and H1p did not favour IMPT and are not included in the narrative summary below, however, outcomes for all analyses are provided in Table 7.

*Interdisciplinary Multimodal Pain Treatment will offer greater improvements than Treatment as Usual for the Health Related Quality of Life domain: Vitality*.

Analyses for short and intermediate term follow up reported findings in favour of IMPT over TAU. *I* ^2^ was < 60% for long term follow‐up and so this outcome was categorised as ‘probably clinically relevant’. For short term follow up *I* ^2^ = 81% in the main analysis, with both subsequent sensitivity analyses reporting small, significant effects in favour of IMPT with admissible *I* ^2^ values (17% and 0%, respectively). In turn this outcome was categorised as *maybe clinically relevant’*. In sum, support for H1e was found at short and intermediate term, but not long‐term follow‐up.

*Interdisciplinary Multimodal Pain Treatment will offer greater improvements than Treatment as Usual for the Health Related Quality of Life domain: Social Functioning*.

There was a small, significant, positive effect in favour of the intervention compared to TAU at intermediate term follow up (SMD = 0.26; 95% CI (0.11; 0.41)). Follow‐up sensitivity analyses excluding studies of lower quality supported the direction, size and statistical significance of the main analysis (SMD = 0.24; 95% CI (0.04; 0.44)). Secondary sensitivity analysis excluding significant outliers was not applied as heterogeneity estimates were within bounds for the main analysis. In sum, this outcome was classified as a *probably* clinically relevant effect.

*Interdisciplinary Multimodal Pain Treatment will offer greater improvements than Active Control Groups for the Health Related Quality of Life domain: Pain*.

Evidence in support of H1j was reported at intermediate (SMD = 0.20; 95% CI (−0.1; 0.49)), and long‐term follow‐up (SMD = 0.36; 95% CI (−0.42; 1.13)). Sensitivity analyses excluding lower‐quality studies (SMD = 0.25; 95% CI [−0.02, 0.51]) and significant outliers (SMD = 0.25; 95% CI [0.05, 0.45]) both yielded small significant effects for intermediate‐term follow‐up. Given the non‐significance of the main analysis, the outcome was categorised as ‘maybe clinically relevant*’*. For long‐term follow‐up, sensitivity analysis excluding lower‐quality studies supported the main analysis (SMD = 0.49; 95% CI (−0.42, 1.41)) but remained non‐significant. Both analyses exceeded the heterogeneity threshold (*I* ^2^ > 60%). A further sensitivity analysis excluding significant outliers indicated a reduced but significant positive effect (SMD = 0.27; 95% CI (0.04, 0.49)). Consequently, this outcome was categorised as ‘*maybe clinically relevant*’. In sum support for H1j was found at intermediate and long‐term follow‐up but not short.

*Interdisciplinary Multimodal Pain Treatment will offer greater improvements than Active Control Groups for the Health Related Quality of Life domain: Social Functioning*.

There was a small, significant, positive effect in favour of the intervention compared to ACG at long term follow up (SMD = 0.47; 95% CI (−0.17; 1.11)). Follow‐up sensitivity analyses excluding studies of lower quality supported the direction, size and statistical significance of the main analysis (SMD = 0.55; 95% CI (−0.21; 1.31)). Secondary sensitivity analysis excluding significant outliers also supported the direction, size and statistical significance of the main analysis SMD = 0.22; 95% CI (0.01; 0.43) and lowered *I* ^2^ to 20%. High heterogeneity in the main analysis means this cannot be classified as a small *probably* clinically relevant effect, however support and improvement from the sensitivity analyses means this outcome is classified as *maybe* clinically relevant.

To conclude: Exploratory analyses revealed novel positive findings in favour of IMPT for four outcomes across varied follow‐up points.

*A greater number of positive effects in favour of the intervention will be found in analysis comparing Interdisciplinary Multimodal Pain Treatment with Treatment as Usual rather than Interdisciplinary Multimodal Pain Treatment versus Active Control Groups* (Marin et al. 2017).

Outcomes reporting *probably/maybe* clinically relevant outcomes in favour of IMPT (*n* = 18) were predominantly found in TAU comparisons (*n* = 14); see Table 7.

Of the 39 analyses conducted, seven were categorised as *probably* clinically relevant, 11 were categorised as *maybe* clinically relevant and 21 were rated as *probably not* clinically relevant.

Specifically, positive findings favouring IMPT over TAU were found for: Emotional Functioning and Mental Health, Pain and General Health at all Follow‐up points; Physical Functioning and Wellbeing at short and long‐term Follow‐up; Vitality at short and intermediate‐term Follow‐up; and Social Functioning at intermediate‐term Follow‐up.

Comparisons with ACGs indicated IMPT offered small positive effects for Pain, and Social Functioning at long‐term follow‐up, Physical Functioning and Wellbeing at intermediate follow‐up and Emotional Functioning and Mental Health at short‐term follow‐up.

*A greater number of positive effects in favour of Interdisciplinary Multimodal Pain Treatment are expected at short‐term Follow‐up compared to intermediate or long‐term Follow‐up* (Chipchase et al. 2013; Williams et al. 2012).

There was no significantly greater number of outcomes favourable to IMPT at short term (*n* = 5), compared to intermediate (*n* = 7) or long term (*n* = 6) follow‐up.

*Interventions of greater duration will be associated with greater positive effects of Interdisciplinary Multimodal Pain Treatment compared with Treatment as Usual or ACG* (Romm et al. 2021).

Finally, there was no effect of intervention duration, indexed by the number of programme hours, found in any analyses. An example of subgroup analysis outcomes is shown in Appendix S11, with full details available upon request from the first author.

### 3.3 Risk of Bias (RoB) Within Studies

**FIGURE 3:**
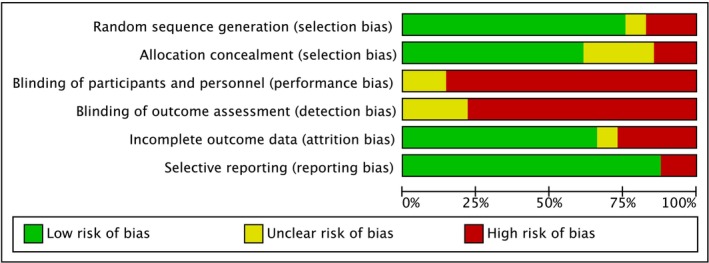
Risk of bias using the GRADE method for all included studies (*n* = 41).

The overall RoB for studies was high, with the quality of evidence as evaluated by the GRADE process found to be ‘very low’ in all but two outcomes (see Table 6). Challenges related to blinding of participants and practitioners (performance bias) were a consistent feature across all studies, with 35 rated as high risk, 6 rated as unclear and 0 rated as low risk. Detection bias was also found to be problematic, with no studies identified as having low risk, eight being rated as high, and 33 being rated as unclear. As can be seen from Figure 3, other domains of RoB scored better, with selection bias and reporting bias having particularly low levels of risk across studies.

**FIGURE 4:**
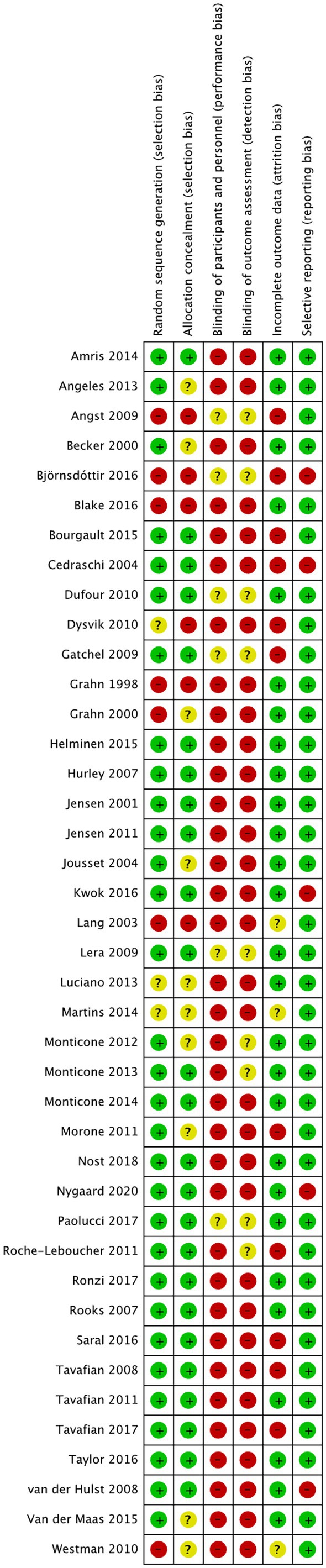
Individual summary of risk of bias for all studies included in the sample.

When considering each included study separately, some perform above average across RoB categories (Ronzi et al. 2017; Taylor et al. 2016), but 12 studies struggled to provide adequate explanation of how bias was managed across at least four of the six categories (Björnsdóttir et al. 2016; Lang et al. 2003). See Figure 4 for an overview of each study's risk of bias assessment.

### 3.4 Potential for Greater Focusing of Analyses

The authors considered whether restricting certain aspects of the study might provide greater clarity on the size or nature of effects. They explored whether limiting the analysis to a specific pain condition and a single validated measure could offer improved clinical insight and applicability.

To test this, an additional analysis was conducted. To maximise the available sample size, the authors focused on the most common pain condition in the dataset (non‐specific chronic low back pain) and the most frequently used HRQoL measure (SF‐36). This approach identified 12 relevant studies, which were then grouped based on comparisons with TAU (*n* = 5) or ACG (*n* = 7). The larger of these two groups was selected for comparative analysis and further separated by follow‐up time points (short‐term: *n* = 6, intermediate: *n* = 1, long‐term: *n* = 5). The short‐term group had a larger total participant count and was chosen for final analysis, with emotional functioning and wellbeing being selected as the domain for analysis (details of this process and the final sample are provided in Appendix S12).

To illustrate the impact of these restrictions on the analysis: in the full sample of 10 studies (SMD = 0.36; 95% CI (0.02; 0.87); *p* = 0.02; *I* ^2^ = 77%); in the restricted sample of five studies (SMD = 0.34; 95% CI (−0.60; 1.27); *p* = 0.319; *I* ^2^ = 84.51%). These findings indicate that, despite similarity in the reported effect sizes, restricting the analysis to a single pain condition and measurement tool did not reduce heterogeneity (*I* ^2^) or narrow the confidence intervals. Accordingly, further analyses based on this restrictive approach were not conducted.

## 4 Discussion and Conclusion

Interdisciplinary Multimodal Pain Treatments (IMPT) are recommended for people with CP to improve HRQoL (IASP 2024). This review is the first to use amalgamated HRQoL subscales to report all HRQoL domains for IMPT for CP. We also examined comparator group, length of Follow‐up, and intervention intensity. Findings support hypotheses 1a–d that IMPT would outperform Treatment as Usual (TAU) for Physical Functioning, Pain, General Health and Emotional Functioning (NICE 2021; Romm et al. 2021). Regarding exploratory analyses, findings revealed additional improvements in HRQoL for Pain (versus ACG at short and intermediate Follow‐up), Vitality (versus TAU at short and intermediate Follow‐up) and Social Functioning (versus both TAU and ACG); thus, there was support for H1e, H1f, H1k and H1o, respectively. Hypothesis two, that IMPT would yield more positive effects versus TAU (seven *probably* clinically relevant) than IMPT versus ACG (zero *probably* clinically relevant), was supported. However, hypothesis three was not supported: there was not a greater number of short‐term effects compared to intermediate or long effects (see Table 7). Hypothesis four was also not supported: interventions of greater duration were not associated with greater positive effects of IMPT compared with TAU or ACG. Heterogeneity was high and overall evidence quality was rated low using the GRADE criteria. These points are explored below.

The review confirms earlier evidence that IMPT offer small, but probably clinically relevant, benefits over TAU in most, but not all, domains of HRQoL. Additionally, exploratory analyses suggest improvements in a smaller number of HRQoL domains compared to ACG, which previous reviews did not report (Romm et al. 2021). The greater statistical power from combining various HRQoL subscales across more studies may explain our new findings. Findings supported hypothesis two, showing that IMPT yield more positive outcomes versus TAU than when compared to ACGs (Marin et al. 2017). ACGs are often included to simulate aspects of the intervention and tend to produce greater placebo effects as well as including some active components (e.g., exercise), which help explain these differences (Laursen et al. 2020). So, some comparisons of IMPT versus ACG may be less likely to yield positive effects compared to IMPT versus TAU; for example, comparing IMPT with ACGs, that frequently include exercise, should be less likely to reveal benefits in the vitality and physical functioning domains, which was the case. To provide greater clarity of the benefits of IMPT we advocate that future studies include both TAU and ACG, where possible.

Regarding hypothesis three, while it was expected that more significant findings would occur at shorter follow‐up points, this was only the case for two outcomes comparing IMPT versus TAU: Emotional Functioning and Wellbeing and Vitality. Conversely, for General Health comparing IMPT versus TAU and Pain comparing IMPT versus ACG there were increasing effects over time. These delayed effects may result from participants becoming more skilled at using intervention tools (Oslund et al. 2009) or reflect better intervention benefits over time (Shaikh and Hapidou 2018). Intermittent effects were found for Physical Functioning and Wellbeing for IMPT versus TAU, Social Functioning for IMPT versus TAU and Emotional Functioning and Wellbeing for IMPT versus ACG. Potential reasons for these outcomes are less clear and require further investigation but, it appears that benefits in some domains appear early and are maintained, while others show patterns of either increasing or decreasing over time. These differences may reflect the dynamic and varied nature, not only of the different components included in IMPT, but also the multidimensional nature of HRQoL and CP, and may be indicative of the holistic and dynamic processes operating within IMPT.

With respect to hypothesis four, interventions lasting 100 h or more did not lead to greater benefit than those under 30 h or those between 31 and 99 h. This contrasts with earlier findings (Romm et al. 2021). On reflection, while total hours is a commonly reported measure of intervention exposure, it does not account for other elements such as intensity, but these are reported inconsistently. Deviation between our own findings and those of Romm et al. (2021) may also be partially linked with the difference in approach taken, with Romm focusing on the number of sessions rather than just duration. Waterschoot et al. (2014) further discuss the need to consider the interaction between duration and content to gain a better idea of the nature of an intervention. Evidence indicates that the relationship between duration, content, number of sessions, to name a few factors, is complicated and non‐linear (Romm et al. 2021).

High estimates of heterogeneity (*I* ^2^ > 60%) were a feature in 23 of the 39 main analyses. A conservative approach was used to manage this and bolster confidence in our findings. As per Deeks et al. (2023), sensitivity analysis excluding outliers was applied to lower *I* ^2^. In 14 of the 23 cases, *I* ^2^ remained high; in the remaining nine cases, *I* ^2^ dropped sufficiently, and the corresponding outcome was then classified as *maybe* clinically relevant (see Appendix S7). Notably, the application of sensitivity analysis never turned an initially non‐significant main analysis into a positive one; it only supported outcomes already favouring IMPT. All sensitivity analyses that led to a *maybe* clinically relevant classification reported small or medium effect sizes consistent with their corresponding main effects, but with *I* ^2^ values within parameters and CIs not crossing 0. Some outcomes, such as Pain versus TAU, were limited by small numbers of studies; the outcomes also had low heterogeneity. In most cases (*n* = 14), this approach did not favour IMPT and underscores the conservative nature of the approach. In sum, despite higher heterogeneity, no outcomes deemed *probably* or *maybe* clinically relevant violated heterogeneity thresholds, had CIs crossing 0, or showed SMD < 0.2. Accordingly, the findings here likely offer a reliable estimate of effects.

GRADE evaluations rated evidence quality as *moderate* for only two outcomes; the remaining 37 outcomes were rated *very low*. A consistent weakness that influences GRADE evaluations was study design, especially issues with blinding participants and practitioners. Hohenschurz‐Schmidt et al. (2023) discuss this challenge in non‐pharmacological pain interventions, and our analyses support that improvements in blinding should be a priority for future research. To check if low‐quality evidence affected outcomes, the main analyses were compared with sensitivity analyses using only higher‐quality studies. Results were statistically congruent in 27 cases and incongruent in seven. In these inconsistent cases, the sensitivity analyses were less reliable; this was due to either few studies (e.g., Pain TAU Short, *n* = 2), or high heterogeneity (e.g., Pain ACG Long, *I* ^2^ = 91.99%), or confidence intervals that crossed zero (e.g., General Health TAU Short); these analyses do not provide clear indication of divergence in outcomes between the analytical approaches. Thus, there is little indication that the low quality of the evidence skewed the main effects or altered estimates of clinical relevance.

This review has two main limitations. First, heterogeneity in the findings, which likely arose from differences in the CP population and the IMPT delivery team, and variations in the intervention components. Consequently, the review restricted analysis to one specific pain group (Low Back Pain) and measure (SF‐36; see Section 3.4), but this did not improve the clarity of the results, likely due to a reduced number of studies and overall sample size. Second, generalizability is somewhat limited by an imbalanced sex ratio; the total sample comprised 6613 participants, with 4759 women. While a balanced sex ratio is often preferred in research, our sample reflects clinical practice. Women are more often referred and participate in these interventions (Samulowitz et al. 2018), and they tend to report lower quality of life (Morales‐Fernández et al. 2021). Research also suggests that women might gain more from IMPT than men (Pieh et al. 2012). Despite these limitations, the current findings remain clear and robust.

In conclusion, this review provides an evaluation of IMPT's impact on all domains of HRQoL for CP, offering comparisons with both TAU and ACG at short, intermediate and long‐term follow‐up. It presents novel findings that show improvements across several HRQoL domains, including IMPT's superiority over ACGs in reducing the impact of pain on daily life—a key goal in pain management. These outcomes are partly attributable to the review's strengths in analysis and design. This review is the first to combine all available HRQoL data, creating larger samples that boost statistical power. Despite some heterogeneity, comprehensive management of this and robust sensitivity analyses further minimised uncertainty related to lower‐quality studies (Deckert et al. 2016). For practitioners, these findings endorse the continued use of IMPT to achieve small to medium improvements in HRQoL that are likely to be clinically relevant. Researchers are encouraged to work with practitioners to provide clearer evidence of IMPT efficacy by reducing heterogeneity.

## Author Contributions

This study was primarily designed and carried out by A.T. All authors contributed to the design and application of robust sample selection and data extraction processes and the results were critically examined by all authors. A.T. had a primary role in preparing the manuscript, which was edited by D.S. and F.M. All authors have approved the final version of the manuscript and agree to be accountable for all aspects of the work.

## Funding

The authors have nothing to report.

## Conflicts of Interest

The authors declare no conflicts of interest.

## Supporting information


**Appendix S1:** ejp70176‐sup‐0001‐AppendicesS1‐S12.docx.

**Data S1:** ejp70176‐sup‐0002‐DataS1.docx.

## References

[ejp70176-bib-0001] Amris, K. , E. E. Wæhrens , R. Christensen , H. Bliddal , and B. Danneskiold‐Samsøe . 2014. “Interdisciplinary Rehabilitation of Patients With Chronic Widespread Pain: Primary Endpoint of the Randomized, Nonblinded, Parallel‐Group IMPROvE Trial.” Pain 155, no. 7: 1356–1364. 10.1016/j.pain.2014.04.012. 24727345

[ejp70176-bib-0002] Angeles, R. N. , D. Guenter , L. McCarthy , et al. 2013. “Group Interprofessional Chronic Pain Management in the Primary Care Setting: A Pilot Study of Feasibility and Effectiveness in a Family Health Team in Ontario.” Pain Research & Management 18, no. 5: 237–242. 10.1155/2013/491279. 23875181 PMC3805345

[ejp70176-bib-0003] Angst, F. , M. L. Verra , S. Lehmann , R. Brioschi , and A. Aeschlimann . 2009. “Clinical Effectiveness of an Interdisciplinary Pain Management Programme Compared With Standard Inpatient Rehabilitation in Chronic Pain: A Naturalistic, Prospective Controlled Cohort Study.” Journal of Rehabilitation Medicine 41, no. 7: 569–575. 10.2340/16501977-0381. 19543669

[ejp70176-bib-0004] Becker, N. , P. Sjøgren , P. bech , A. Kornelius Olsen , and J. Eriksen . 2000. “Treatment Outcome of Chronic Non‐Malignant Pain Patients Managed in a Danish Multidisciplinary Pain Centre Compared to General Practice: A Randomised Controlled Trial.” Pain 84, no. 2–3: 203–211. 10666525 10.1016/s0304-3959(99)00209-2

[ejp70176-bib-0005] Beckie, T. M. , and L. A. Hayduk . 1997. “Measuring Quality of Life.” Social Indicators Research 42, no. 1: 21–39. 10.1023/A:1006881931793.

[ejp70176-bib-0084] Björnsdóttir, S. V. , M. Arnljótsdóttir , G. Tómasson , J. Triebel , and U. A. Valdimarsdóttir . 2016. “Health‐Related Quality of Life Improvements Among Women with Chronic Pain: Comparison of Two Multidisciplinary Interventions.” Disability and Rehabilitation 38, no. 9: 828–836. 10.3109/09638288.2015.1061609. 26122546

[ejp70176-bib-0006] Björnsson, J. K. , K. Tomasson , S. Ingimarsson , and T. Helgason . 1997. “Health‐Related Quality of Life of Psychiatric and Other Patients in Iceland: Psychometric Properties of the IQL.” Nordic Journal of Psychiatry 51, no. 3: 183–191. 10.3109/08039489709109093.

[ejp70176-bib-0007] Blake, C. , J. Cunningham , C. K. Power , S. Horan , O. Spencer , and B. M. Fullen . 2016. “The Impact of a Cognitive Behavioral Pain Management Program on Sleep in Patients With Chronic Pain: Results of a Pilot Study.” Pain Medicine (Malden, Mass.) 17, no. 2: 360–369. 26352702 10.1111/pme.12903

[ejp70176-bib-0008] Bourgault, P. , A. Lacasse , S. Marchand , et al. 2015. “Multicomponent Interdisciplinary Group Intervention for Self‐Management of Fibromyalgia: A Mixed‐Methods Randomized Controlled Trial.” PLoS One 10, no. 5: e0126324. 10.1371/journal.pone.0126324. 25978402 PMC4433106

[ejp70176-bib-0009] Bowling, A. 2017. Measuring Health. McGraw‐Hill Education.

[ejp70176-bib-0010] Cashin, A. G. , B. M. Furlong , S. J. Kamper , et al. 2025. “Analgesic Effects of Non‐Surgical and Non‐Interventional Treatments for Low Back Pain: A Systematic Review and Meta‐Analysis of Placebo‐Controlled Randomised Trials.” BMJ Evidence‐Based Medicine 30: 222–232. 10.1136/bmjebm-2024-112974. 40101974

[ejp70176-bib-0011] Cedraschi, C. , J. Desmeules , E. Rapiti , et al. 2004. “Fibromyalgia: A Randomised, Controlled Trial of a Treatment Programme Based on Self Management.” Annals of the Rheumatic Diseases 63, no. 3: 290–296. 10.1136/ard.2002.004945. 14962965 PMC1754921

[ejp70176-bib-0012] Chipchase, L. , D. Sheffield , and P. Hill . 2013. “The Long‐Term Effectiveness of Pain Management Programs.” In Pain: International Research in Pain Management, 175–192. Nova Science Publishers, Inc.

[ejp70176-bib-0013] Cohen, J. 1988. J. Statistical Power Analysis in the Behavioral Sciences. 2nd ed. Lawrence Erlbaum Associates.

[ejp70176-bib-0014] Cohen, S. P. , L. Vase , and W. M. Hooten . 2021. “Chronic Pain: An Update on Burden, Best Practices, and New Advances.” Lancet (London, England) 397, no. 10289: 2082–2097. 10.1016/S0140-6736(21)00393-7. 34062143

[ejp70176-bib-0015] Deckert, S. , U. Kaiser , C. Kopkow , F. Trautmann , R. Sabatowski , and J. Schmitt . 2016. “A Systematic Review of the Outcomes Reported in Multimodal Pain Therapy for Chronic Pain.” European Journal of Pain 20: 51–63. 10.1002/ejp.721. 26031689

[ejp70176-bib-0016] Deeks, J. , J. Higgins , and D. Altman . 2023. “Chapter 10: Analysing Data and Undertaking Meta‐Analyses: 10.10.3 Strategies for Addressing Heterogeneity.” In Cochrane Handbook for Systematic Reviews of Interventions (6.4), edited by J. P. T. Higgins and J. Thomas . Cochrane Collaborations. https://training.cochrane.org/handbook/current/chapter‐10.

[ejp70176-bib-0017] Dong, H.‐J. , B. Gerdle , and E. Dragioti . 2022. “Reported Outcomes in Interdisciplinary Pain Treatment: An Overview of Systematic Reviews and Meta‐Analyses of Randomised Controlled Trials.” Journal of Pain Research 15: 2557–2576. 10.2147/JPR.S362913. 36065439 PMC9440697

[ejp70176-bib-0018] Dufour, N. , G. Thamsborg , A. Oefeldt , C. Lundsgaard , and S. Stender . 2010. “Treatment of Chronic Low Back Pain: A Randomized, Clinical Trial Comparing Group‐Based Multidisciplinary Biopsychosocial Rehabilitation and Intensive Individual Therapist‐Assisted Back Muscle Strengthening Exercises.” Spine 35, no. 5: 469–476. 10.1097/BRS.0b013e3181b8db2e. 20147878

[ejp70176-bib-0019] Dysvik, E. , J. T. Kvaløy , R. Stokkeland , and G. K. Natvig . 2010. “The Effectiveness of a Multidisciplinary Pain Management Programme Managing Chronic Pain on Pain Perceptions, Health‐Related Quality of Life and Stages of Change—A Non‐Randomized Controlled Study.” International Journal of Nursing Studies 47, no. 7: 826–835. 10.1016/j.ijnurstu.2009.12.001. 20036362

[ejp70176-bib-0083] Elbers, S. , H. Wittink , S. Konings , et al. 2022. “Longitudinal Outcome Evaluations of Interdisciplinary Multimodal Pain Treatment Programmes for Patients with Chronic Primary Musculoskeletal Pain: A Systematic Review and Meta‐Analysis.” European Journal of Pain 26, no. 2: 310–335. 10.1002/ejp.1875. 34624159 PMC9297911

[ejp70176-bib-0020] Gatchel, R. J. , D. D. McGeary , C. A. McGeary , and B. Lippe . 2014. “Interdisciplinary Chronic Pain Management: Past, Present, and Future.” American Psychologist 69, no. 2: 119–130. 10.1037/a0035514. 24547798

[ejp70176-bib-0021] Gatchel, R. J. , D. D. McGeary , A. Peterson , et al. 2009. “Preliminary Findings of a Randomized Controlled Trial of an Interdisciplinary Military Pain Program.” Military Medicine 174, no. 3: 270–277. 10.7205/MILMED-D-03-1607. 19354091

[ejp70176-bib-0022] Grahn, B. , C. Ekdahl , and L. Borgquist . 1998. “Effects of a Multidisciplinary Rehabilitation Programme on Health‐Related Quality of Life in Patients With Prolonged Musculoskeletal Disorders: A 6‐Month Follow‐Up of a Prospective Controlled Study.” Disability and Rehabilitation 20, no. 8: 285–297. 10.3109/09638289809166084. 9651687

[ejp70176-bib-0023] Grahn, B. , C. Ekdahl , and L. Borgquist . 2000. “Motivation as a Predictor of Changes in Quality of Life and Working Ability in Multidisciplinary Rehabilitation: A Two‐Year Follow‐Up of a Prospective Controlled Study in Patients With Prolonged Musculoskeletal Disorders.” Disability and Rehabilitation 22, no. 15: 639–654. 10.1080/096382800445443. 11087060

[ejp70176-bib-0024] Hadi, M. A. , G. A. McHugh , and S. J. Closs . 2019. “Impact of Chronic Pain on Patients' Quality of Life: A Comparative Mixed‐Methods Study.” Journal of Patient Experience 6, no. 2: 133–141. 10.1177/2374373518786013. 31218259 PMC6558939

[ejp70176-bib-0025] Helminen, E.‐E. , S. H. Sinikallio , A. L. Valjakka , R. H. Väisänen‐Rouvali , and J. P. Arokoski . 2015. “Effectiveness of a Cognitive‐Behavioural Group Intervention for Knee Osteoarthritis Pain: A Randomized Controlled Trial.” Clinical Rehabilitation 29, no. 9: 868–881. 10.1177/0269215514558567. 25413168

[ejp70176-bib-0026] Higgins, J. P. T. , D. G. Altman , P. C. Gotzsche , et al. 2011. “The Cochrane Collaboration's Tool for Assessing Risk of Bias in Randomised Trials.” BMJ (Clinical Research Ed.) 343: d5928. 10.1136/bmj.d5928. PMC3196245 22008217

[ejp70176-bib-0027] Higgins, J. P. T. , T. Li , and J. Deeks . 2019. Chapter 6: Choosing Effect Measures and Computing Estimates of Effect. Wiley. https://training.cochrane.org/handbook/current/chapter‐06.

[ejp70176-bib-0028] Hochheim, M. , P. Ramm , and V. Amelung . 2023. “The Effectiveness of Low‐Dosed Outpatient Biopsychosocial Interventions Compared to Active Physical Interventions on Pain and Disability in Adults With Nonspecific Chronic Low Back Pain: A Systematic Review With Meta‐Analysis.” Pain Practice 23, no. 4: 409–436. 10.1111/papr.13198. 36565010

[ejp70176-bib-0029] Hohenschurz‐Schmidt, D. , J. Draper‐Rodi , L. Vase , et al. 2023. “Blinding and Sham Control Methods in Trials of Physical, Psychological, and Self‐Management Interventions for Pain (Article II): A Meta‐Analysis Relating Methods to Trial Results.” Pain 164, no. 3: 509–533. 10.1097/j.pain.0000000000002730. 36271798 PMC9916063

[ejp70176-bib-0030] Hurley, M. V. , N. E. Walsh , H. L. Mitchell , et al. 2007. “Clinical Effectiveness of a Rehabilitation Program Integrating Exercise, Self‐Management, and Active Coping Strategies for Chronic Knee Pain: A Cluster Randomized Trial.” Arthritis & Rheumatism 57, no. 7: 1211–1219. 10.1002/art.22995. 17907147 PMC2673355

[ejp70176-bib-0031] IASP . 2024. “Pain Management Center—Chapter 1 International Association for the Study of Pain (IASP).” https://www.iasp‐pain.org/resources/toolkits/pain‐management‐center/chapter1/.

[ejp70176-bib-0032] IASP . 2025. “Pain Terminology.” https://www.iasp‐pain.org/resources/terminology/.

[ejp70176-bib-0033] Jayadevappa, R. , R. Cook , and S. Chhatre . 2017. “Minimal Important Difference to Infer Changes in Health‐Related Quality of Life—A Systematic Review.” Journal of Clinical Epidemiology 89: 188–198. 10.1016/j.jclinepi.2017.06.009. 28676426

[ejp70176-bib-0034] Jensen, C. , O. K. Jensen , D. H. Christiansen , and C. V. Nielsen . 2011. “One‐Year Follow‐Up in Employees Sick‐Listed Because of Low Back Pain: Randomized Clinical Trial Comparing Multidisciplinary and Brief Intervention.” Spine 36, no. 15: 1180–1189. 10.1097/BRS.0b013e3181eba711. 21217456

[ejp70176-bib-0035] Jensen, I. B. , G. Bergström , T. Ljungquist , L. Bodin , and A. L. Nygren . 2001. “A Randomized Controlled Component Analysis of a Behavioral Medicine Rehabilitation Program for Chronic Spinal Pain: Are the Effects Dependent on Gender?” Pain 91, no. 1–2: 65–78. 11240079 10.1016/s0304-3959(00)00420-6

[ejp70176-bib-0036] Jones, I. , L. Williams , P. Wilkinson , et al. 2021. Guidelines for Pain Management Programmes for Adults. British Pain Society. https://www.britishpainsociety.org/static/uploads/resources/files/Guidelines_for_PMP_01082019_xc33xiN.pdf.

[ejp70176-bib-0037] Jousset, N. , S. Fanello , L. Bontoux , et al. 2004. “Effects of Functional Restoration Versus 3 Hours Per Week Physical Therapy: A Randomized Controlled Study.” Spine 29, no. 5: 487–493. 10.1097/01.BRS.0000102320.35490.43. 15129059

[ejp70176-bib-0038] Kaiser, U. , C. Kopkow , S. Deckert , et al. 2018. “Developing a Core Outcome Domain Set to Assessing Effectiveness of Interdisciplinary Multimodal Pain Therapy: The VAPAIN Consensus Statement on Core Outcome Domains.” Pain 159, no. 4: 673–683. 10.1097/j.pain.0000000000001129. 29300277

[ejp70176-bib-0039] Karimi, M. , and J. Brazier . 2016. “Health, Health‐Related Quality of Life, and Quality of Life: What Is the Difference?” PharmacoEconomics 34, no. 7: 645–649. 10.1007/s40273-016-0389-9. 26892973

[ejp70176-bib-0040] Kwok, E. Y. T. , R. K. C. Au , and C. W. P. Li‐Tsang . 2016. “The Effect of a Self‐Management Program on the Quality‐Of‐Life of Community‐Dwelling Older Adults With Chronic Musculoskeletal Knee Pain: A Pilot Randomized Controlled Trial.” Clinical Gerontologist 39, no. 5: 428–448. 10.1080/07317115.2016.1171818. 29471771

[ejp70176-bib-0041] Lang, E. , K. Liebig , S. Kastner , B. Neundörfer , and P. Heuschmann . 2003. “Multidisciplinary Rehabilitation Versus Usual Care for Chronic Low Back Pain in the Community: Effects on Quality of Life.” Spine Journal 3, no. 4: 270–276. 10.1016/S1529-9430(03)00028-7. 14589185

[ejp70176-bib-0042] Laursen, D. R. , C. Hansen , A. S. Paludan‐Müller , and A. Hróbjartsson . 2020. “Active Placebo Versus Standard Placebo Control Interventions in Pharmacological Randomised Trials.” Cochrane Database of Systematic Reviews 2020, no. 7: MR000055. 10.1002/14651858.MR000055. PMC9989326 36877132

[ejp70176-bib-0043] Lawlis, G. F. , R. Cuencas , D. Selby , and C. E. McCoy . 1989. “The Development of the Dallas Pain Questionnaire. An Assessment of the Impact of Spinal Pain on Behavior.” Spine 14, no. 5: 511–516. 10.1097/00007632-198905000-00007. 2524890

[ejp70176-bib-0044] Lera, S. , S. M. Gelman , M. J. Lopez , et al. 2009. “Multidisciplinary Treatment of Fibromyalgia: Does Cognitive Behavior Therapy Increase the Response to Treatment?” Journal of Psychosomatic Research 67, no. 5: 433–441. 10.1016/j.jpsychores.2009.01.012. 19837206

[ejp70176-bib-0045] Lins, L. , and F. M. Carvalho . 2016. “SF‐36 Total Score as a Single Measure of Health‐Related Quality of Life: Scoping Review.” SAGE Open Medicine 4: 2050312116671725. 10.1177/2050312116671725. 27757230 PMC5052926

[ejp70176-bib-0046] Luciano, J. V. , R. Sabes‐Figuera , E. Cardeñosa , and T. Peñarrubia‐María . 2013. “Cost‐Utility of a Psychoeducational Intervention in Fibromyalgia Patients Compared With Usual Care: An Economic Evaluation Alongside a 12‐Month Randomized Controlled Trial.” Clinical Journal of Pain 29, no. 8: 702–711. 10.1097/AJP.0b013e318270f99a. 23328339

[ejp70176-bib-0047] Marin, T. J. , D. Van Eerd , E. Irvin , et al. 2017. “Multidisciplinary Biopsychosocial Rehabilitation for Subacute Low Back Pain.” Cochrane Database of Systematic Reviews 6: CD002193. 10.1002/14651858.CD002193.pub2. 28656659 PMC6481490

[ejp70176-bib-0048] Martins, M. R. I. , C. C. Gritti , R. d. S. Junior , et al. 2014. “Randomized Controlled Trial of a Therapeutic Intervention Group in Patients With Fibromyalgia Syndrome.” Revista Brasileira de Reumatologia (English Edition) 54, no. 3: 179–184. 10.1016/j.rbre.2013.10.002. 25054594

[ejp70176-bib-0049] Mishra, S. I. , R. W. Scherer , P. M. Geigle , et al. 2012. “Exercise Interventions on Health‐Related Quality of Life for Cancer Survivors.” Cochrane Database of Systematic Reviews 8: CD007566. 10.1002/14651858.CD007566.pub2. PMC7387117 22895961

[ejp70176-bib-0050] Monticone, M. , E. Ambrosini , B. Rocca , S. Magni , F. Brivio , and S. Ferrante . 2014. “A Multidisciplinary Rehabilitation Programme Improves Disability, Kinesiophobia and Walking Ability in Subjects With Chronic Low Back Pain: Results of a Randomised Controlled Pilot Study.” European Spine Journal 23, no. 10: 2105–2113. 10.1007/s00586-014-3478-5. 25064093

[ejp70176-bib-0051] Monticone, M. , P. Baiardi , C. Vanti , et al. 2012. “Chronic Neck Pain and Treatment of Cognitive and Behavioural Factors: Results of a Randomised Controlled Clinical Trial.” European Spine Journal 21, no. 8: 1558–1566. 22466021 10.1007/s00586-012-2287-y PMC3535234

[ejp70176-bib-0052] Monticone, M. , S. Ferrante , B. Rocca , P. Baiardi , F. D. Farra , and C. Foti . 2013. “Effect of a Long‐Lasting Multidisciplinary Program on Disability and Fear‐Avoidance Behaviors in Patients With Chronic Low Back Pain: Results of a Randomized Controlled Trial.” Clinical Journal of Pain 29, no. 11: 929–938. 10.1097/AJP.0b013e31827fef7e. 23328343

[ejp70176-bib-0053] Morales‐Fernández, Á. , J. M. Jiménez Martín , M. Vergara‐Romero , et al. 2021. “Gender Differences in Perceived Pain and Health‐Related Quality of Life in People With Chronic Non‐Malignant Pain: A Cross‐Sectional Study.” Contemporary Nurse 57, no. 3–4: 280–289. 10.1080/10376178.2021.1999836. 34709980

[ejp70176-bib-0054] Morone, G. , T. Paolucci , M. R. Alcuri , et al. 2011. “Quality of Life Improved by Multidisciplinary Back School Program in Patıents With Chronic Non‐Specific Low Back Pain: A Single Blind Randomized Controlled Trial.” European Journal of Physical and Rehabilitation Medicine 47, no. 4: 533–541. 21508915

[ejp70176-bib-0055] Mouelhi, Y. , E. Jouve , C. Castelli , and S. Gentile . 2020. “How Is the Minimal Clinically Important Difference Established in Health‐Related Quality of Life Instruments? Review of Anchors and Methods.” Health and Quality of Life Outcomes 18, no. 1: 136. 10.1186/s12955-020-01344-w. 32398083 PMC7218583

[ejp70176-bib-0056] NICE . 2021. “Chronic Pain (Primary and Secondary) in Over 16s: Assessment of All Chronic Pain and Management of Chronic Primary Pain. NICE Guideline, NG193.” https://www.nice.org.uk/guidance/ng193/resources/chronic‐pain‐primary‐and‐secondary‐in‐over‐16s‐assessment‐of‐all‐chronic‐pain‐and‐management‐of‐chronic‐primary‐pain‐pdf‐66142080468421. 33939353

[ejp70176-bib-0057] Nøst, T. H. , A. Steinsbekk , O. Bratås , and K. Grønning . 2018. “Short‐Term Effect of a Chronic Pain Self‐Management Intervention Delivered by an Easily Accessible Primary Healthcare Service: A Randomised Controlled Trial.” BMJ Open 8, no. 12: e023017. 10.1136/bmjopen-2018-023017. PMC6303596 30530580

[ejp70176-bib-0058] Nygaard, A. S. , M. B. Rydningen , M. Stedenfeldt , et al. 2020. “Group‐Based Multimodal Physical Therapy in Women With Chronic Pelvic Pain: A Randomized Controlled Trial.” Acta Obstetricia et Gynecologica Scandinavica 99, no. 10: 1320–1329. 10.1111/aogs.13896. 32386466

[ejp70176-bib-0059] Oslund, S. , R. C. Robinson , T. C. Clark , et al. 2009. “Long‐Term Effectiveness of a Comprehensive Pain Management Program: Strengthening the Case for Interdisciplinary Care.” Proceedings (Baylor University Medical Center) 22, no. 3: 211–214. 19633738 10.1080/08998280.2009.11928516 PMC2709080

[ejp70176-bib-0060] Paolucci, T. , F. Zangrando , M. Iosa , et al. 2017. “Improved Interoceptive Awareness in Chronic Low Back Pain: A Comparison of Back School Versus Feldenkrais Method.” Disability and Rehabilitation 39, no. 10: 994–1001. 10.1080/09638288.2016.1175035. 27215948

[ejp70176-bib-0061] Pieh, C. , J. Altmeppen , S. Neumeier , T. Loew , M. Angerer , and C. Lahmann . 2012. “Gender Differences in Outcomes of a Multimodal Pain Management Program.” Pain 153, no. 1: 197–202. 10.1016/j.pain.2011.10.016. 22100358

[ejp70176-bib-0062] Roche‐Leboucher, G. , A. Petit‐Lemanac'h , L. Bontoux , et al. 2011. “Multidisciplinary Intensive Functional Restoration Versus Outpatient Active Physiotherapy in Chronic Low Back Pain: A Randomized Controlled Trial.” Spine 36, no. 26: 2235–2242. 10.1097/BRS.0b013e3182191e13. 21415807

[ejp70176-bib-0063] Romm, M. J. , S. Ahn , I. Fiebert , and L. P. Cahalin . 2021. “Rehabilitation & Regenerative Medicine Section: A Meta‐Analysis of Group‐Based Pain Management Programs: Overall Effect on Quality of Life and Other Chronic Pain Outcome Measures, With an Exploration Into Moderator Variables That Influence the Efficacy of Such Interventions.” Pain Medicine 22, no. 2: 407–430. 10.1093/pm/pnaa376. 33582811

[ejp70176-bib-0064] Ronzi, Y. , G. Roche‐Leboucher , C. Bègue , et al. 2017. “Efficiency of Three Treatment Strategies on Occupational and Quality of Life Impairments for Chronic Low Back Pain Patients: Is the Multidisciplinary Approach the Key Feature to Success?” Clinical Rehabilitation 31, no. 10: 1364–1373. 10.1177/0269215517691086. 28592147

[ejp70176-bib-0065] Rooks, D. S. 2007. “Group Exercise, Education, and Combination Self‐Management in Women With FibromyalgiaA Randomized Trial.” Archives of Internal Medicine 167, no. 20: 2192. 10.1001/archinte.167.20.2192. 17998491

[ejp70176-bib-0066] Samulowitz, A. , I. Gremyr , E. Eriksson , and G. Hensing . 2018. ““Brave Men” and “Emotional Women”: A Theory‐Guided Literature Review on Gender Bias in Health Care and Gendered Norms Towards Patients With Chronic Pain.” Pain Research & Management 2018: 6358624. 10.1155/2018/6358624. 29682130 PMC5845507

[ejp70176-bib-0067] Saral, I. , D. Sindel , S. Esmaeilzadeh , A. Oral , and H. O. Sertel‐Berk . 2016. “The Effects of Long‐ and Short‐Term Interdisciplinary Treatment Approaches in Women With Fibromyalgia: A Randomized Controlled Trial.” Rheumatology International 36, no. 10: 1379–1389. 27055444 10.1007/s00296-016-3473-8

[ejp70176-bib-0068] Schünemann, H. , J. Brożek , G. Guyatt , and A. Oxman . 2013. GRADE Handbook. GRADE Working Group. https://gdt.gradepro.org/app/handbook/handbook.html.

[ejp70176-bib-0069] Schünemann, H. , G. Vist , J. Higgins , et al. 2023. “Chapter 15: Interpreting Results and Drawing Conclusions −15.5.3.1 Presenting and Interpreting SMDs Using Generic Effect Size Estimates.” In Cochrane Handbook for Systematic Reviews of Interventions. John Wiley & Sons Ltd. https://training.cochrane.org/handbook/current/chapter‐15.

[ejp70176-bib-0070] Shaikh, M. , and E. G. Hapidou . 2018. “Factors Involved in Patients' Perceptions of Self‐Improvement After Chronic Pain Treatment.” Canadian Journal of Pain 2, no. 1: 145–157. 10.1080/24740527.2018.1476821. 35005374 PMC8730591

[ejp70176-bib-0071] Suurmond, R. , H. Van Rhee , and T. Hak . 2017. “Introduction, Comparison, and Validation of Meta‐Essentials: A Free and Simple Tool for Meta‐Analysis.” Research Synthesis Methods 8, no. 4: 537–553. 10.1002/jrsm.1260. 28801932 PMC5725669

[ejp70176-bib-0072] Tavafian, S. S. , A. R. Jamshidi , and K. Mohammad . 2011. “Treatment of Chronic Low Back Pain: A Randomized Clinical Trial Comparing Multidisciplinary Group‐Based Rehabilitation Program and Oral Drug Treatment With Oral Drug Treatment Alone.” Clinical Journal of Pain 27, no. 9: 811–818. 10.1097/AJP.0b013e31821e7930. 21642845

[ejp70176-bib-0073] Tavafian, S. S. , A. R. Jamshidi , and K. Mohammad . 2017. “Treatment of Low Back Pain: Second Extended Follow Up of an Original Trial (NCT00600197) Comparing a Multidisciplinary Group‐Based Rehabilitation Program With Oral Drug Treatment Alone Up to 30 Months.” International Journal of Rheumatic Diseases 20, no. 12: 1910–1916. 10.1111/1756-185X.12540. 25546488

[ejp70176-bib-0074] Tavafian, S. S. , A. R. Jamshidi , and A. Montazeri . 2008. “A Randomized Study of Back School in Women With Chronic Low Back Pain: Quality of Life at Three, Six, and Twelve Months Follow‐Up.” Spine 33, no. 15: 1617–1621. 10.1097/BRS.0b013e31817bd31c. 18580739

[ejp70176-bib-0075] Taylor, S. J. C. , D. Carnes , K. Homer , et al. 2016. “Novel Three‐Day, Community‐Based, Nonpharmacological Group Intervention for Chronic Musculoskeletal Pain (COPERS): A Randomised Clinical Trial.” PLoS Medicine 13, no. 6: e1002040. 10.1371/journal.pmed.1002040. 27299859 PMC4907437

[ejp70176-bib-0076] Treede, R.‐D. , W. Rief , A. Barke , et al. 2019. “Chronic Pain as a Symptom or a Disease: The IASP Classification of Chronic Pain for the International Classification of Diseases (ICD‐11).” Pain 160, no. 1: 19. 10.1097/j.pain.0000000000001384. 30586067

[ejp70176-bib-0077] van der Hulst, M. , M. M. R. Vollenbroek‐Hutten , K. G. M. Groothuis‐Oudshoorn , and H. J. Hermens . 2008. “Multidisciplinary Rehabilitation Treatment of Patients With Chronic Low Back Pain: A Prognostic Model for Its Outcome.” Clinical Journal of Pain 24, no. 5: 421–430. 10.1097/AJP.0b013e31816719f5. 18496307

[ejp70176-bib-0078] Van der Maas, L. C. C. , A. Köke , M. Bosscher , et al. 2016. “Improving the Multidisciplinary Treatment of Chronic Pain by Stimulating Body Awareness: A Cluster‐Randomized Trial.” Clinical Journal of Pain 31, no. 7: 660–669. 10.1097/AJP.0000000000000138. 25119509

[ejp70176-bib-0079] Waterschoot, F. P. C. , P. U. Dijkstra , N. Hollak , H. J. de Vries , J. H. B. Geertzen , and M. F. Reneman . 2014. “Dose or Content? Effectiveness of Pain Rehabilitation Programs for Patients With Chronic Low Back Pain: A Systematic Review.” Pain 155, no. 1: 179–189. 10.1016/j.pain.2013.10.006. 24135435

[ejp70176-bib-0080] Westman, A. , S. J. Linton , J. Öhrvik , P. Wahlén , T. Theorell , and J. Leppert . 2010. “Controlled 3‐Year Follow‐Up of a Multidisciplinary Pain Rehabilitation Program in Primary Health Care.” Disability and Rehabilitation 32, no. 4: 307–316. 10.3109/09638280903095924. 20055569

[ejp70176-bib-0081] Wiklund, I. 1990. “The Nottingham Health Profile—A Measure of Health‐Related Quality of Life.” Scandinavian Journal of Primary Health Care. Supplement 1: 15–18. 2100359

[ejp70176-bib-0082] Williams, A. C. d. C. , C. Eccleston , and S. Morley . 2012. “Psychological Therapies for the Management of Chronic Pain (Excluding Headache) in Adults.” Cochrane Database of Systematic Reviews 11, no. 11: CD007407. 10.1002/14651858.CD007407.pub3. 23152245 PMC6483325

