# Supplementary material for: Assessing the Impact of Interdisciplinary Multimodal Pain Treatment on Health‐Related Quality of Life in Chronic Pain: A Systematic Review and Meta‐Analyses
Source: Eur J Pain. 2025 Dec 2;30(1):e70176. doi: 10.1002/ejp.70176 (PMC12670346; doi:10.1002/ejp.70176)
Supplement: Supplementary file 1 — Appendix S1: ejp70176‐sup‐0001‐AppendicesS1‐S12.docx. [file EJP-30-0-s002.docx]

**_Appendices_**_:_

[AppendixS1: Intervention inclusion criterion and comparison group details 2](#_Toc207178148)

[AppendixS2: Databases included in search 3](#_Toc207178149)

[AppendixS3: Search strategies used for different databases: 4](#_Toc207178150)

[Strategy: Applied to University of Derby EBSCOhost, Web of Science, ASSIA, Dimensions and CORE databases. 4](#_Toc207178151)

[Strategy 2: Google Scholar 4](#_Toc207178152)

[Strategy 3: Prospero 5](#_Toc207178153)

[Strategy 4: Clinicaltrials.gov 5](#_Toc207178154)

[Strategy 5: UK clinical trials gateway 5](#_Toc207178155)

[Strategy 6: Dimensions 5](#_Toc207178156)

[Strategy 7: NIHR 5](#_Toc207178157)

[Strategy 8: EBSCO 5](#_Toc207178158)

[AppendixS4: Risk of Bias Assessment 5](#_Toc207178159)

[AppendixS5: GRADE Approach to Evidence Synthesis 7](#_Toc207178160)

[AppendixS6: Subscales group by domain: 8](#_Toc207178161)

[AppendixS7**:** Estimating Clinical Relevance 12](#_Toc207178162)

[AppendixS8: Characteristics of included and excluded studies. 13](#_Toc207178163)

[AppendixS9: Overview of all measures and subscales included in the analyses. 99](#_Toc207178164)

[AppendixS10: Supporting passage for the reading of table 7 101](#_Toc207178165)

[AppendixS11: Example of subgroup analysis by intervention duration 101](#_Toc207178166)

[AppendixS12: Description and sample for potential/trailed narrowing of analyses 102](#_Toc207178167)

#

# AppendixS1: Intervention inclusion criterion and comparison group details

Studies including an IMPT were admissible for inclusion in this review. Criteria for IMPT classification entailed interventions which included a physical intervention and adopted at least one other modality from a biopsychosocial approach to treatment (i.e. psychological, social), and which was facilitated by professionals from at least 2 different professional domains (e.g. physician, physiotherapist, occupational therapist, social workers, psychologist). The intervention could either be multidisciplinary, or interdisciplinary but should be part of a single unified approach and ideally should include an aspect of group treatment in at least one stage or component of the intervention. These criteria are reflective of earlier reviews considering the impact on IMPT on other conditions or outcomes (Marin et al., 2017). Studies which included 2 IMPT groups (and no other control groups) were eligible for inclusion, providing the groups were of different intensities, with the lower duration version serving as an active control condition for the higher duration group.

**Physical component**

Appropriate physical components of the IMPT could include treatments such as functional restoration, massage, back school, manual therapy, physiotherapy, exercise therapy. Surgical intervention and pharmacological interventions were excluded as suitable modalities, this reflects approaches within earlier reviews of a similar nature (Marin et al., 2017). Further to this, as noted in Higgins (2011) when making comparisons between contrastingly different interventions such as surgery vs. multimodal treatment, is it inherently impossible to blind participants to their allocated group, essentially guaranteeing a high risk of performance bias within these comparisons.

**Psychological Component**

Modalities which were admissible under the psychological component included approaches such as CBT based interventions, pain-education, counselling directed at addressing personal factors such as fears, beliefs, or emotions.

**Social Component**

Approaches which included consideration or intervention directly related to aspects of occupational vocational or social dimensions were sought for this aspect. Ideally facilitated by an appropriate professional, this included things such as working or family environments or roles, engagement with wider social groups, and/or personal/professional networks.

Marin, T. J., Van Eerd, D., Irvin, E., Couban, R., Koes, B. W., Malmivaara, A., van Tulder, M. W., & Kamper, S. J. (2017). Multidisciplinary biopsychosocial rehabilitation for subacute low back pain. *The Cochrane Database of Systematic Reviews*, *6*, CD002193. <https://doi.org/10.1002/14651858.CD002193.pub2>

**Comparison groups:**

Active Control Group (ACG): A comparison group receiving an alternative intervention for chronic pain, which may or may not follow a biopsychosocial approach.

Waiting List Control (WLC): A group of participants scheduled to receive the same intervention as the IMPT group at a later time.

Treatment as Usual (TAU): A group receiving only standard care for their pain condition from their healthcare provider. They are not awaiting the IMPT intervention and do not receive any significant additional therapeutic intervention.

# AppendixS2: Databases included in search

- “*Library Plus*” (University of Derby, EBSCO host)

MEDLINE, Science Citation Index, PsycINFO, Academic Search Index, Social Sciences Citation Index, CINAHL Plus, Complementary Index, SPORTDiscus with Full Text, ScienceDirect, Academic OneFile, Supplemental Index, Directory of Open Access Journals, AMED - The Allied and Complementary Medicine Database, Cochrane Database of Systematic Reviews, Business Source Complete, British Library EThOS, General OneFile, British Education Index, IEEE Xplore Digital Library.

- *“Web of Science”*

MEDLINE, BIOSIS citation index, SciELO citation index.

- Prospero
- Zetoc
- Clinical trials.gov
- CORE
- Google Scholar
- Dimensions
- NIHR (National Institute of Health Research)
- ASSIA (Applied Social Science Index and Abstracts)
- EBSCO

# AppendixS3: Search strategies used for different databases:

## Strategy: Applied to University of Derby EBSCOhost, Web of Science, ASSIA, Dimensions and CORE databases.

Condition:

*(“chronic* pain*” OR “Long-term pain*” OR “Long-term pain” OR “Persistent pain*” OR “Chronic* Neuropathic pain” OR “Chronic* Musculoskeletal Pain” OR “Chronic* headache” OR “Chronic* migrane” OR “chronic* discomfort” OR “chronic* ache*” OR “chronic* fibromyalgia” OR “chronic* neuralgi*” OR “chronic* dysmenorrh*”)*

Intervention:

(“multidisciplinary” OR “interdisciplinary” OR “multiprofessional” OR “multi-professional” OR “multimodal” OR “multi-modal” OR “functional restoration” OR “biopsychosocial” OR “Pain* Manage*” OR “PMP”)

Outcome:

(“activities of daily living” OR “Life Quality” OR “QOL” OR “wellness” OR “well being” OR “functional ability” OR “Short Form 36” OR “Short-Form 36” OR “Short Form 12” OR “Short-Form 12” OR “EQ-5D” OR “15D” OR *“Quality of life” OR QoL OR “SF-36” OR “SF-12” OR “*EuroQoL”)

Design:

(“Longitudinal” OR “Prospective” OR “Follow-up” OR “Follow-up”)

AND

(“randomized controlled trial” OR “randomised controlled trial” OR “RCT” OR “controlled clinical trial” OR “CCT” OR “random*” OR “placebo” OR “randomly” OR “trial” OR “groups” OR “single blind” OR “double blind” OR “triple blind”)

*Limiters*: Adult population, papers in English language

(“Protocol”)

OR

("children" OR "adolescent" OR "youth" OR "child" OR "teenager" OR "teen")

OR

("internet" OR "web" OR "online")

## Strategy 2: Google Scholar

Full search string adapted to satisfy the 256-character limit of Google scholar entry.

*("chronic* pain*" OR "Long-term pain*" OR "Persistent pain*") AND ("multidisciplinary" OR "multimodal") AND (“Quality of life" OR "QOL" OR "wellness" OR "wellbeing") AND ("random* controlled trial" OR "RCT" OR "controlled clinical trial" OR "CCT")*

## Strategy 3: Prospero

“*chronic pain*”

## Strategy 4: Clinicaltrials.gov

*‘Chronic pain’* AND ‘*health related quality of life’*

Limiters: ‘completed studies’ and ‘Adult’ + ‘older adult’.

## Strategy 5: UK clinical trials gateway

‘*Pain management’*,

Limiters: ‘completed studies’ and ‘Adult’

## Strategy 6: Dimensions

## Strategy 7: NIHR

*"chronic pain"* AND *"Quality of life"* AND *"Pain management"*

Limiters: published findings

## Strategy 8: EBSCO

('chronic pain'/exp OR 'chronic pain') AND ('quality of life'/exp OR 'quality of life')

Limiters: Adults, RCTs, Articles, Chronic Pain

# AppendixS4: Risk of Bias Assessment

Higgins, J. P., Altman, D. G., Gøtzsche, P. C., Jüni, P., Moher, D., Oxman, A. D., ... & Sterne, J. A. (2011). The Cochrane Collaboration’s tool for assessing risk of bias in randomised trials. *bmj*, *343*.

As outlined by(Higgins et al., 2011) - Studies selected for inclusion will be assessed for risk of bias across the 5 categories outlined below. Information will be drawn from within published articles, study protocols, and where possible directly from study authors upon request. In line with Cochrane guidance “Studies are assessed as at unclear risk of bias when too few details are available to make a judgement of ‘high’ or ‘low’ risk”.

Selection bias

This will aim to establish how participants were allocated to groups, and whether a method of randomisation (either by physical or digital means), or quasi randomisation (such as allocating based on patient number or date of birth) was applied. In studies where an explanation of how either of these methods was applied is provided, a rating low will be given. Where a source reports a process which grouped participants in a way that is not randomised or quasi-randomised (such as grouping by age, or symptom severity) the source will be rated as having high risk of bias. In cases where it is not possible to discern either way the source will be rated as ‘unclear’.

Performance bias

This domain will seek to establish if either the practitioner or participant aware of their group allocation during the intervention. The biopsychosocial, collaborative, and on-going nature of the intervention considered within this report, makes the successful blinding of both parties extremely difficult. To be rated as having a ‘low’ risk of bias, studies will need to clearly outline how this was achieved. In cases where blinding was impossible due to differences between group conditions (for example active intervention vs treatment as usual), a rating of ‘high’ will be given. In cases where no process is described, bit it is reasonable to assume that its likely blinding could have taken place due to the design or description of the study, a rating of ‘unclear’ may be awarded.

Detection bias

This category will seek to establish whether the person assessing the outcome blinded to group allocation. It should be noted that in all studies included within the present analysis adopted self-report measures, meaning it is the participant as well as the person carrying out the analysis who must be blinded. In cases where a study has been rated as having a high risk of bias in the performance bias category due to non-blinded participants, use of self-report measures necessitate that the category of detection bias will also be considered as having a high risk of bias.

Attrition bias

This category will seek to establish whether a study has explained a strategy for managing missing data and participant drop out. Studies using an Intention to Treat approach, as well as other appropriate missing data strategies will receive a rating of ‘low’. Studies adopting a Per Protocol, or similar approach which doesn’t account for bias created by missing data will be rated as ‘high’. In cases where no missing data strategy is described and findings report outcomes from analysis carried out with the impaired data, the study will be rated as ‘high’.

Reporting bias

In cases where a study protocol is available, comparison will be made between the health-related quality of life measures which were intended for inclusion, with the measures included in the final report. In cases where no protocol is available comparison will be made within papers between the measures outlined in the methods section and the findings reported in the results. In both of the cases just described; where there is accordance between the two parts, papers will be rated as having a ‘low’ risk of bias. This is in line with the approach taken in earlier similar reviews considering IMPT interventions (Martin et al., 2017).

# AppendixS5: GRADE Approach to Evidence Synthesis

The quality of the body of evidence for each outcome will be graded as either; ‘*High’*, ‘*Moderate’* ‘*Low’*, or ‘*Very Low’,* in line with the GRADE process. A rating of high indicates that there is a high degree of reliability in the data, and it is expected that future studies are unlikely to change the outcome presented here. Moderate indicates that there is a reasonable potential for future studies to influence confidence in the effect. Low indicates that there is a high likelihood that future studies will impact the confidence in the main effect. Very Low indicates a very low level of certainty in the current estimates provided.

Quality of evidence was evaluated in the follow way:

1. Study design

Studies which were RCTs and quasi-RCTs, evidence will begin from a rating of ‘high’ and be downgraded based on the criterion outlined below.

1. Risk of Bias

Studies will be assessed according to the procedures outlined in the section describing the RoB process preceding this one. Evidence will be downgraded by 1 point when there are three or fewer categories in each study included within the sample is rated to have bias or unclear bias. In cases where four or more categories are judged to have bias or unclear bias a deduction of 2 points will be made.

1. Inconsistency

Consideration will be given to estimates of heterogeneity within the data for all outcomes. In cases where I^2^ > 60%, data will be downgraded by 1 point.

1. Indirectness

Potential confounds exist within ‘indirectness’ when there is a difference between intended and actual focus with the studies included in the analysis, including: A; different follow-up time points, B; the use of surrogate or indirect measures, and C; when there is not equality of comparison between the groups in different studies. Addressing each of these:

1. The method in the present review will categorise and analyse different follow-up time points separately as outlined in the methods section.
2. A selection criterion for studies to be included in the review is that they include a specific, self-report tool which directly h measures self-reported quality of life (see eligibility criterion in methods section)
3. Analysis will be separated out between treatment as usual and active control groups to reduce risk in this area. Subgroup analysis will also be carried out looking at any differences that may exist between differing intensities of intervention (see ‘Quantitative analyses and main outcomes’ in methods section)

Despite these measures to reduce risk in this area, consideration will still be given to the overall directness within the included sources in line with these areas.

1. Imprecision

The present review will include continuous outcomes, and so imprecision will be considered with respect to the size of the sample of studies, and the overall population size.

In cases where there was n<2 for number of sources, or the total population was n<400, evidence was downgraded by one point. In cases where there was no significant effect and confidence interval cross an effect size of 0.5 in either direction, evidence was downgrade by 1 point.

# AppendixS6: Subscales group by domain:

Items recovered during the search process included a number of different HRQoL measures. Scales and subscales included in the analysis are listed below in section 1. In section 2, the combined constructs are presented, detailing which subscales were allocated to each construct.

*This approach was adapted from:*

Mishra, S. I., Scherer, R. W., Snyder, C., Geigle, P. M., Berlanstein, D. R., & Topaloglu, O. (2012). Exercise interventions on health‐related quality of life for people with cancer during active treatment. *Cochrane Database of Systematic Reviews*, (8).

**Section 1 – overview of all HRQoL measures included within the Meta Analysis sample:**

Items below which are ‘struck-though’ were not selected for inclusion in the combined subscales due to not being a clear enough fit, or not being relevant to any of the planned constructs.

**Short Form 36 (SF-36) (Ware 1999)**

**&**

**Short Form 12 (SF-12)**

Subscales:

Physical functioning

Role physical

pain

general health

vitality

social functioning

role emotional

Mental health

Physical component score

Mental component score

**Nottingham Health Profile (NHP)** (Wiklund, 1990)

Subscales:

Energy

Pain

Emotional reactions

Social isolation

Sleep disruption

Physical mobility

Total score

**Icelandic Quality of Life scale (IQL)** (Björnsson et al., 1997)

Subscales:

general health

~~depression~~

social functioning

~~financial status~~

energy

~~anxiety~~

physical health

Pain

~~self-control~~

sleep

general wellbeing

**Dallas pain questionnaire (DPQ)** (Lawlis et al., 1989)

Subscales:

Activities of Daily Living (After thought, decided not to include this in part of the physical functioning combined construct as the questions include things such as amount of pain, and interference with sleep).

Work/Leisure

Anxiety/Depression (while these factors were excluded in the IQL, assessment of the items in the DPQ were judged to align with

Social Interest

**EQ-5D-3L & Euroqol 5d-5L (**Rabin,and Charro (2001).

mobility,

self-care,

usual activities,

pain/discomfort

anxiety/depression.

VAS scale

**Section 2 – Overview of the amalgamated constructs used within the current Meta Analysis, and detail of the different subscales which were included within these:**

**Physical functioning and wellbeing**

SF12 + 36 Physical functioning

SF12 + 36 Role Physical

SF12 + 36 PCS

NHP physical mobility

IQL physical health

**Pain**

SF12 + 36 Pain

NHP pain

IQOL Pain

**General health**

SF12 + 36 general health

IQL general health

**Vitality/Energy**

SF12 + 36 Vitality

NHP energy

IQL energy

**Social functioning**

SF12 + 36 social functioning

IQL - Social functioning

NHP - social isolation

DPQ- social interest

**emotional function / Mental Health**

SF12+36 Mental Health

SF12+36 MCS

NHP Emotional reactions

SF12+36 role emotional

**Sleep**

IQL Sleep

NHP sleep disruption

**Overall HRQoL**

NHP total score

EQ-5D-3L

EuroQol

Euroqol 5d

lisat-9

# AppendixS7**:** Estimating Clinical Relevance

When considering an approach to estimating clinical relevance, it is generally agreed that for patient reported outcomes, patient evaluations of what constitutes meaningful change should be the more valued factor than those clinicians’ evaluation of meaningful change (Revicki et al., 2008).

In a recent review(Mouelhi et al., 2020) , the approaches adopted to estimate MIDs in measures of HRQoL varied greatly, even differing between researchers adopting the same measure in different studies. There is no single best approach or estimate and guidelines recommend adopting a blend of anchored and distribution-based methods for best practice. Anchor methods allow for the establishment of minimally important differences (MIDs) in one measure via benchmark comparisons with the changes seen in another measure is gathered at the same time (Mouelhi et al., 2020).

In some cases where MIDs have been established in an earlier study, these can be used in, however values such as these are highly specific to the measure, population, disease, and intervention. As such, unless there is high congruence values such as these are often not transferable (Jayadevappa et al., 2017). Further, (Schünemann et al., 2023);

*“The approach, however, relies on having well-established MIDs. The approach is also risky in that a difference less than the MID may be interpreted as trivial when a substantial proportion of patients may have achieved an important benefit”.*

In the present study estimating minimal clinical difference (MID) as an approach was considered but rejected, due to lack of a suitable anchor measure and the use of established MIDs not being possible owning to the design of HRQoL measurement in the current study (combining SMDs rather than using a single established psychometric scale) and earlier studies not having applied this approach.

Due to this, a purely distributional approach must be adopted here, several possible approaches to this are discussed (Mouelhi et al., 2020). An approach based on SMD and Cohen’s established effect sizes (0.2=small; 0.5= medium; 0.8= large) (Cohen, 1988) was selected as the most practical and reliable method for the current review (Schünemann et al., 2023). For chronic pain conditions known to be highly intransigent to treatment, and which associated with ongoing decline in quality of life over time. In the current study, even small positive effects in favour of the intervention will be considered as potentially clinically relevant. Outcomes are categorised as either ‘*probably clinically relevant’* ‘*maybe clinically relevant’* and ‘*probably not clinically relevant*’ (terms used in earlier relevant Cochrane reviews (Kamper et al., 2014) (Marin et al., 2017), the categorisation will be based on the effect size, heterogeneity estimates, width of CI and if they cross 0, outlier, risk of bias, and trim and fill sensitivity analysis as per the criterion presented below:

Clinical significance categories:

1./ If a main outcome reports a small, medium, or large effect size in favour of the intervention, which is within heterogeneity parameters and has confidence intervals that do not cross zero this will be considered ‘probably clinically relevant’.

2./ If a main outcome reports a small, medium, or large effect size but has heterogeneity estimates out with the set parameters and/or confidence interval that cross zeros which are all later resolved with analysis excluding outliers this will be considered ‘maybe clinically relevant’.

3./ If a main outcome reports a small, medium, or large effect size but has heterogeneity estimates out with the set parameters and/or confidence interval that cross zeros which are all later resolved with analysis on higher quality studies only, this will be considered ‘maybe clinically relevant’.

4./ In cases of category 1 (above), but where subsequent trim and fill analysis has reported an adjusted model and the number of sources in sample is 10 or greater (Page et al., 2019), this adjusted effect size will be considered in place of the main effect.

- In cases where the adjusted SMD is equal to or greater than .2, and CIs don’t cross 0, this will be considered ‘*probably clinically relevant’*.
- In cases where the adjusted SMD is equal to or greater than .2, and CIs do cross 0, this will be considered ‘*probably not clinically relevant’*.
- In cases where SMD<.2, this will be considered ‘*probably not clinically relevant*’.

Nb: In cases of category 1 (above) where subsequent trim and fill analysis has reported an adjusted model but the number of sources in sample is less than 10, this adjusted effect size and indicted asymmetry in the sample will be disregarded as per (Page et al., 2019).

5./ If a main outcome doesn’t report a SMD >.2, or has heterogeneity estimates out with the set parameters and/or confidence interval that cross zeros, and these factors are not resolved with the application of either risk of bias or analysis excluding outliers, then this outcome will be considered ‘probably not clinically’ significant.

# AppendixS8: Characteristics of included and excluded studies.

Amris 2014

| **Methods** | **Title**: Interdisciplinary rehabilitation of patients with chronic widespread pain: Primary endpoint of the randomized, nonblinded, parallel-group IMPROvE trial  Randomized, controlled, open-label, single-centre trial with 2 arms.  **Location**: Denmark. |
| --- | --- |
| **Participants** | n=191 female patients aged 18+ diagnosed with Chronic Widespread Pain.  **Intervention group**  (Baseline: n = 96) mean age (SD) = 44.4 (10.9) . Eight (8%) withdrew from the intervention group before intervention, assessment at the primary time point  (6-month follow-up); n= 84 (87%)  **Waiting list control group**  (Baseline: n = 95) mean age (SD) =44.2 (10.8). (9%) withdrew from the control group during the 6-month intervention period.  (6-month follow-up) n= 86 (90%) in control group |
| **Interventions** | **Comparison:** Intervention vs Waiting list control  I**npatient or Outpatient Setting**: Out  **Intervention Duration**: Medium  **Group or individual intervention**: Group based  **MBI intervention details:**  a 2-week non residential, group-based, multi-component treatment course conducted by a multidisciplinary team consisting of a rheumatologist, a psychologist, a nurse, and occupational and physical therapists. The treatment course had a scheduled program each day, with a daily time schedule between 3 and 5 hours; in total 35 hours. The treatment course was based on an interactive, participatory approach and comprised a combination of presentations and group discussions, as well as instructions during physical exercise and performance of ADL tasks. A 2-hour session with the participants and their relatives conducted by the psychologist and the rheumatologist, and a 30-minute individual consultation with the rheumatologist at the end of the course, were included. Further, participants were offered a supplementary 3-hour session with the Danish Association for Fibromyalgia Patients focusing on patient perspective and social legislation counselling. |
| **Outcomes** | **Outcomes**:  Motor and Process Skills (AMPS)  SF-36 Mental Composite Score (MCS) and SF-36 PCS and  SF-36 'physical function' sub-scale  **Follow-up time points**:  evaluated at baseline and 6-month follow-up. |
| **Notes** | Supported by grants from The Oak Foundation, Schioldanns Fond, and The Danish Rheumatism Association  Average age of participant relatively low compared to other studies in the review.  whole sample was female |

Risk of bias table

| **Bias** | **Authors' judgement** | **Support for judgement** |
| --- | --- | --- |
| Random sequence generation (selection bias) | Low risk | Quote: *"participants were randomly assigned to either the 2-week multicomponent treatment course or control group with a 1:1 allocation, per a computer-generated randomization schedule stratifying patients using minimization by baseline AMPS-motor (<1.0 vs ‘‘1.0 to <1.5’’ vsP1.5), AMPS-process (<1.0 vs P1.0) and a computer-generated list of random numbers."* |
| Allocation concealment (selection bias) | Low risk | *"The random allocation sequence was concealed until interventions were assigned"* |
| Blinding of participants and personnel (performance bias) | High risk | Waiting list control used, title of the paper includes 'non-blinded' |
| Blinding of outcome assessment (detection bias) | High risk | While AMPS trained research assistant blinded for group allocation performed all AMPS evaluations at the 6-month follow-up. However this was not the case for all psychometric measures, in cases of self report the participant was the assessor and was no blinded. |
| Incomplete outcome data (attrition bias) | Low risk | Intention to treat |
| Selective reporting (reporting bias) | Low risk | No protocol, however, all measures included in methods listed in results |

Angeles 2013

| **Methods** | **Title**: Group interprofessional chronic pain management in the primary care setting: A pilot study of feasibility and effectiveness in a family health team in Ontario Randomised control trial with 2 groups- WLC  **Location**: Canada |
| --- | --- |
| **Participants** | **Intervention group**  n= 29 58% female Mean age: 56.88; SD:14.34  **Comparison group;**  n= 34 66.7% female Mean age: 53.12; SD:13.46 |
| **Interventions** | **In/out:** Out  **group**/**individual**: group  **Intensities**: Low  **Exp**:  The program consisted of 2 h group sessions once per week for eight weeks. Group sessions included the following: education regarding the nature of chronic pain, pacing and goal setting, and medication management; practice in mindfulness relaxation techniques; cognitive reflection on beliefs, impulses and obsessional thoughts about pain; and practice of physical activation techniques (modified fitness and practice of modified postures or positions for various activities of daily living, including self-care and housekeeping tasks).  **Waiting list control**:  Served as control group then were transferred to treatment group after 8 months. |
| **Outcomes** | **Outcomes:**  SF36  Health care utilization  **Follow up Timepoints:** Short, Med  Pre, Post, 6 month FU |
| **Notes** | Small sample. Study reports 240 recruited but only 63 agreed to take part (63% female) |

Risk of bias table

| **Bias** | **Authors' judgement** | **Support for judgement** |
| --- | --- | --- |
| Random sequence generation (selection bias) | Low risk | Patients who consented and completed the baseline study questionnaires were randomly assigned to either EI or DI groups using the online Research Randomizer. |
| Allocation concealment (selection bias) | Unclear risk | information not included |
| Blinding of participants and personnel (performance bias) | High risk | Significant differences in the timing between groups (8 months), with the control group on a waiting list.  "*At the outset, all participants were informed of the process and interventions they would receive but not informed about the group to which they were assigned. Individuals in the DI group were informed that they were wait-listed and that the intervention would be delivered based on a schedule. All participants were followed-up for six months after the end of the intervention*"  It is reasonable to assume if participants are informed that there will be one group which will have a delayed start, a portion of participants in this group would guess this was their allocation and so would not be blinded. |
| Blinding of outcome assessment (detection bias) | High risk | Information not included. However, as a self report measure is used and blinding of participants from group allocation was not clearly possible there is a high risk of bias in this domain. |
| Incomplete outcome data (attrition bias) | Low risk | Intention to Treat applied |
| Selective reporting (reporting bias) | Low risk | No protocol cited, all measures from methods included in results section |

Angst 2009

| **Methods** | **Title**: Clinical effectiveness of an interdisciplinary pain management programme compared with standard inpatient rehabilitation in chronic pain  non-randomised clinical trial comparing a high and medium duration IMPT.  **Location**: Zurzach, Bad Zurzach, Switzerland |
| --- | --- |
| **Participants** | **Eligibility/recruitment**  cohort consisted of n=331 chronic pain patients who were included between 2002 and 2007.  *"All subjects included in the study: (i) had either chronic non-specific back pain (i.e. lumbar, thoracic, cervical, or panvertebral pain syndrome), or fibromyalgia according to the definition of the American College of Rheumatology (ACR), or chronic widespread pain, i.e. generalized musculoskeletal pain syndrome that did not meet the definition criteria of fibromyalgia. Further inclusion criteria were: (ii) ability to complete self-assessment questionnaires, especially sufficient German language, and psycho-intellectual abilities; and (iii) written informed consent."*  *"For the interdisciplinary pain management programme, each candidate was assessed by means of a standardized telephone interview regarding motivation, realistic aims, and cognitive abilities (in order to intellectually understand the contents of cognitive behavioural therapy) by the head of the programme (RB). This procedure ensured standardized and valid criteria for inclusion into the programme and patients’ potential of rehabilitation. Additionally, for each participant, special permission for the costs had to be obtained from the relevant health insurance company."*  **intervention**:  baseline n=171 (79% female )mean age: 45.3 (10.6)  graduated n=164  6 month follow-up n=107  **control**:  baseline n=160 (78% female) Mean age: 53.4 (12.4)  graduated n=143  6 month follow-up n=103  The drop-out rates at discharge were 4.1% for the intervention group vs 10.6% for the control group and 34.7% vs 28.0% at the 6 month follow-up. |
| **Interventions** | **In/out**: In  **Duration**: High and med  **group/individual**: mix  Comparison of two IMPT groups, one high and one med duration: physiotherapy, aerobic endurance training, Qigong/tai chi exercises, individual psychotherapy including cognitive behavioural therapy, participation in a pain coping group, relaxation therapy, humour therapy, information and education about pathophysiology of pain mechanisms and management of chronic disabling pain.  **Intervention**; 104 hours (approx.)  **Control:** 68 hours |
| **Outcomes** | **Outcomes**  SF-36,  The West Haven-Yale Multidimensional Pain Inventory (WHYMPI),  Coping Strategies Questionnaire (CSQ),  Hospital Anxiety and Depression Scale (HADS).  MPI to assess pain, the SF-36 physical functioning to assess physical function (in general, mainly ambulation), the SF-36 social functioning to asses the ability to participate socially, both HADS depression and anxiety scales to assess affective health specifically and comprehensively, the MPI life control to assess a salutogenic attitude, the CSQ catastrophizing and ability to decrease pain to assess 2 of the most important predictors for successful coping with pain. The main outcome was pain (MPI pain severity) and the other 7 scales were regarded as secondary outcomes.  **Timepoints**: Short, medium  Pre, post (1m FU), 6 month follow-up |
| **Notes** | note the differences in this study: In patient aspect, also includes a blend of group and individual approaches, plus a large gap in number of hours between groups. Also that this was direct comparison between two IMPTs. |

Risk of bias table

| **Bias** | **Authors' judgement** | **Support for judgement** |
| --- | --- | --- |
| Random sequence generation (selection bias) | High risk | "*Patients were consecutively referred by general practitioners, rheumatologists, psychiatrists, and hospitals (mainly departments of rheumatology), either to the interdisciplinary, in patient pain management programme or to regular, standardized inpatient rehabilitation."*  *"This allocation was left to the discretion of the health professionals and was not the result of randomization or any other mechanism*." |
| Allocation concealment (selection bias) | High risk | No, see above. |
| Blinding of participants and personnel (performance bias) | Unclear risk | information not included, however as both groups are IMPT there is possible that participants were not fully aware of their group in relation to others. Theoretically both groups are IMPT and only differ in duration, likelihood of participant knowing comparative intensities is low. However blinding is not |
| Blinding of outcome assessment (detection bias) | Unclear risk | information not included, however, despite the use of self report measures, as there is was potential for participants to be blind to their group allocation (see above) the risk posed here is unclear rather than high. |
| Incomplete outcome data (attrition bias) | High risk | no strategy to compensate for attrition bias detailed, drop out data excluded. |
| Selective reporting (reporting bias) | Low risk | protocol mentioned but not cited clearly. measures reported in methods are present in results. |

Becker 2000

| **Methods** | **Title**: Treatment outcome of chronic non-malignant pain patients managed in a Danish multidisciplinary pain centre compared to general practice: a randomised controlled trial  RCT (august 1994 - October 1996)  **Location**: Copenhagen, Denmark |
| --- | --- |
| **Participants** | **Eligibility**: chronic non-malignant pain conditions who were referred to the Multidisciplinary pain centre from the Copenhagen area. above 18 years, no psychiatric diseases present, no illegal use of opioids.  "*Out of 391 patients consecutively referred to the Pain Centre, 100 patients did not fulfil the inclusion criteria. Consent from the referring GP was not obtained in 97 patients. Five patients did not want to participate in the investigation. Consequently, 189 patient were scheduled for inclusion in the study and randomised. However, 22 patients (11.6%) had to be excluded at the initial consultation. The reasons for exclusion did not differ significantly between the three groups. Six patients withdrew their consent, six patients were unable to fill in questionnaires because of age, impaired cognition or inadequate mastering of the Danish language, ®ve patients had symptoms indicating major psychiatric disease, four patients had illegal use of opioids, and one patient did not need treatment. This left* ***167 patients*** *who were included in the study"*  **MPT-group,**  Baseline n= 56, 60.7% female, mean age: 57.7 (15.8)  6m follow-up n=49  **GP-group**,  Baseline n= 58, 69% female mean age; 55.1 (14.6)  6m follow-up n=42  **WL-group**,  Baseline n= 53, 64% female, mean age; 57.2 (15.5)  6m follow-up n=43  **Attrition**:  "*Thirty-three of the 167 patients (19.8%) were excluded or dropped out after the initial consultation and before the 6 months evaluation: Two patients in the MPT-group were excluded because of manic-depressive disorders, and five patients did not return questionnaires. In the GP-group four patients were excluded for ethical reasons and given MPT, and 12 patients did not return questionnaires. In the WL-group one patient was excluded for ethical reasons and given MPT, and nine patients did not return questionnaires. Patients who were excluded or dropped out of the study did not differ from patients included in the study with respect to demographic data, pain epidemiology or HRQL at referral."* |
| **Interventions** | **In/out:** Out  **group**/**individual**: Individual  **Intensities**: Estimated that it falls into the med cat for Exp group. the other 2 are TAU with GP and wlc with TAU with WLC controls with no duration  **Exp group**:  **MPT-group** received outpatient treatment at the Multidisciplinary Pain Centre. The treatment programme was carried out on an individual basis and when considered necessary planned and initiated after a multidisciplinary evaluation. treatment was of primarily cognitive-behavioural nature and included one or several of the following components: (1) education on the physiology and psychology of pain; (2) teaching of pain management strategies (e.g. relaxation training); (3) analgesic treatment; (4) socio-economic counselling and (5) physiotherapy.  **Comparison group 1**  **GP-group** the intervention consisted of a single initial consultation during which the pain specialist evaluated the patient together with the GP in his/her consultation room. The pain specialist made a medical record and a pain analysis. Based on this the pain specialist and the GP established a treatment plan. Treatment consisted primarily of analgesic tailoring, however, during the consultation the importance of education on the physiology and psychology of pain and behavioural pain management strategies were also emphasized.  **Comparison group 2**  **The WL-group** continued to be managed as usual by their GPs for 6 months. They received no specific pain treatment except for continuation of already ongoing pain medication. After 6 months they began the intervention given to the exp group above. |
| **Outcomes** | **Outcomes**  Measurement of Pain (VAS).the Hospital Anxiety and Depression Scale (HAD) (Zigmond and Snaith, 1983), the Psychological General Well-Being Scale (PGWB) (Dupuy, 1984), and the MOS 36-Item Short Form Health Survey (SF-36)  **Subscales reported:**  SF-36:  RP, PF, P, GH, e/f, SF, RE, ewb.  **Timepoints: *(short, Medium)***  Baseline, 3 months, 6 months. |
| **Notes** | Treatment is provided on an individual basis  Small sample size |

Risk of bias table

| **Bias** | **Authors' judgement** | **Support for judgement** |
| --- | --- | --- |
| Random sequence generation (selection bias) | Low risk | *"Randomisation was performed in blocks of nine. Randomisation within each block was performed using the sealed envelope method.*" |
| Allocation concealment (selection bias) | Unclear risk | information not explicitly provided |
| Blinding of participants and personnel (performance bias) | High risk | not possible due to distinct differences between group conditions |
| Blinding of outcome assessment (detection bias) | High risk | Information not clearly provided, however, as a self report measure is used, and as no adequate blinding of participants from group allocation (see '*performance bias*' category above) was possible, there was estimated to be a high risk of bias in this domain. |
| Incomplete outcome data (attrition bias) | Low risk | Intention to treat approach taken |
| Selective reporting (reporting bias) | Low risk | No protocol. however all measures reported in methods included in results |

Björnsdóttir 2016

| **Methods** | **Title**: Health-related quality of life improvements among women with chronic pain: comparison of two multidisciplinary interventions  CCT. traditional multidisciplinary pain management program (**TMP**) versus neuroscience education and mindfulness-based cognitive therapy (**NEM**) and **WLC**.  **Location**: Iceland |
| --- | --- |
| **Participants** | **Eligible**:  "*Participants in this study were females with chronic pain [25] for at least 6 months, willing to participate in an in-patient group program for 1 month and abstain from other treatment modalities. Reference subjects were recruited from female individuals on a waiting list for an admission to the rehabilitation clinic in 2008. The eligibility criteria included any diagnosis related to chronic musculoskeletal condition and age 18–70 years. Every fifth eligible subject applying to the clinic was contacted by mail and received an information letter and invitation to participate.*"  **Note** (data retrospectively analysed for intervention groups, and WLC 'reference' group recruited for the present study:  "*In this study, we thus compare potential improvements among three cohorts: NEM intervention participants receiving treatment from 2006 to 2009; TMP participants receiving treatment from 2001 to 2005 and reference cohort of subjects on waiting list for admission to that institution."*  n=234 enrolled in the program, 212 women from 23 to 67 years old (mean 49.6 years) participated  n= 122 TMP intervention. Mean age (SD), 48.8(9.2)  n= 90 NEM intervention. Mean age (SD), 50.6 (7.2)  n=57 WLC group. Mean age (SD), 54.7 (10.0) *"Reference subjects were recruited from female individuals on a waiting list for an admission to the rehabilitation clinic in 2008*." |
| **Interventions** | **Inpatient or outpatient**: Inpatient  **Intervention intensities**: High; 100 hours over 4 weeks for both groups (detailed breakdown included in table 1 of original source)  **Group or individual:** Mixed  **Group intervention information**:  Both led by a specialized inter-professional pain management team; physical therapists, a psychologist, a psychiatric nurse, a sport therapist, a rehabilitation physician, a medical massage therapist and access to a nutritionist for specific counselling. both intervention groups participated in body awareness sessions, the physical training for the  **TMP** group emphasized traditional endurance, strength and flexibility training. In the  **NEM** group there was put emphasis on motor control training in conjunction with neuroscience patient education and mindfulness based cognitive therapy (MBCT). |
| **Outcomes** | **Outcomes**:  Information on employment status, sick leave, disability status and educational level was obtained through questionnaires. Information on number of diagnoses and analgesic medication was obtained through patient records at admission. Measurements of height (in cm) and weight (in kg) were also obtained from patient records and body mass index (BMI) calculated as height2/weight. + Pain scores on a 100mm VAS  Icelandic Quality of Life (IQL) scal**e**.  12 domains; general health, concentration, depression, social functioning, financial status, energy, anxiety, physical health, pain, self-control, sleep and general well-being. In addition, the instrument offers a summary score for HRQL.  **Timepoints**: short  baseline, end of programme (31 days). |
| **Notes** | All female sample  Inpatient setting  Retrospective analysis of data from previous years interventions |

Risk of bias table

| **Bias** | **Authors' judgement** | **Support for judgement** |
| --- | --- | --- |
| Random sequence generation (selection bias) | High risk | No. "*Participants in this study were not randomly assigned to the two intervention groups"*  "*In this study, we thus compare potential improvements among three cohorts: NEM intervention participants receiving treatment from 2006 to 2009; TMP participants receiving treatment from 2001 to 2005 and reference cohort of subjects on waiting list for admission to that institution.*" |
| Allocation concealment (selection bias) | High risk | See above |
| Blinding of participants and personnel (performance bias) | Unclear risk | Information not provided |
| Blinding of outcome assessment (detection bias) | Unclear risk | Information not provided |
| Incomplete outcome data (attrition bias) | High risk | no missing data/attrition protocol included |
| Selective reporting (reporting bias) | High risk | No protocol, however all measures reported in methods included in results. |

Blake 2016

| **Methods** | **Title**: The Impact of a Cognitive Behavioural Pain Management Program on Sleep in Patients with Chronic Pain: Results of a Pilot Study  non-randomised pilot trial. IMPT vs WLC  **Location**: Dublin, Ireland |
| --- | --- |
| **Participants** | "*All individuals currently on the waiting list for the CBT- PMP were eligible for inclusion in our study. Potential participants were given written and verbal information regarding the study and invited back to the pain clinic 1 week later where, after all questions had been answered, written consent was obtained. Participants then completed a PSQI, and individuals with a PSQI score of more than five were assigned to either the treatment group or the waiting list control group based upon their position on the waiting list. Individuals having to wait for more than 6 months were used in the control condition, while individuals having to wait less than 6 months were used in the treatment condition*"  **CBT-PMP group**  n=24 male (n=11), females (n=13). mean age (sd) 47.7 (11.5) years,  **WLC group**  n=22 male (n=6), female (n=16). mean age (sd) 46.8 (13.2) years |
| **Interventions** | I**npatient vs outpatient:** Outpatient  **Intervention intensities**: Medium, 72 hours total  **Group or individual approach**: Mixed  **Team:** multidisciplinary team includes a pain management physician, a senior occupational therapist, a senior physiotherapist and a senior clinical psychologist.  **Intervention/experimental group**  "*program runs 3 days a week (6 hours per day) for 4 weeks (six to eight patients per group) with review sessions held 2 and 6 months postprogram completion. daily physiotherapy sessions (one and a half hours): two sessions are gym-based (patient-driven quota-based program), and one session in the hydrotherapy pool (progressive strengthening program). Occupational therapy sessions (one and a half hours daily) focuses on promoting energy conservation techniques and developing pacing strategies for activities of daily living. Daily group psychology sessions focus on CBT principles, and identifying and changing maladaptive behaviours. A number of sleep hygiene and behavioural strategies are also recommended. Pain medicine consultants give weekly 1-hour lectures on a range of topics including explanations of the pain gate control theory; they reinforce CBT-PMP principles and discuss medication reduction strategies."*  **Waiting list control**  "*Patients in the WLC group underwent the same recruitment process as the study participants in terms of giv- ing consent, completing the baseline outcome measures and wearing the Actiwatch. The WLC group did not repeat the battery of questionnaires after 4 weeks, but did so after 12 weeks of usual care."* |
| **Outcomes** | **Outcomes**  Pain [numerical rating scale, (NRS)],  physical function (Simmond’s functional tests),  emotional function [Hospital Anxiety and Depression Scale (HAD-A and HAD-D), Tampa scale of Kinesophobia, (TSK)],  QOL [Short Form 36 (SF-36)],  participant global impression of change and satisfaction, and adverse events and compliance (attendance at follow-ups).  Pittsburgh sleep quality index (PSQI)  **Timepoints**: (short)  Baseline - 2 month follow-up |
| **Notes** | Potential bias of using a WLC for comparison.  small sample size |

Risk of bias table

| **Bias** | **Authors' judgement** | **Support for judgement** |
| --- | --- | --- |
| Random sequence generation (selection bias) | High risk | "*Participants then completed a PSQI, and individuals with a PSQI score of more than five were assigned to either the treatment group or the waiting list control group based upon their position on the waiting list. Individuals having to wait for more than 6 months were used in the control condition, while individuals having to wait less than 6 months were used in the treatment condition*."  clear risk of bias due to potential impact to/homogeneity of people on the waiting list |
| Allocation concealment (selection bias) | High risk | See above. |
| Blinding of participants and personnel (performance bias) | High risk | Not clearly reported. However assuming full consent was given to participants prior, it is reasonable to assume that due to the distinct difference between the groups participants would be aware if they were receiving active intervention or passively waiting. No clear explanation is given for the process or timing whereby people migrated from the waiting list control to the experimental group. |
| Blinding of outcome assessment (detection bias) | High risk | "Group membership was concealed from the principal investigator who completed all the assessments."  However as a self report measure is used and no clear blinding of participants from group allocation has been described, there is a clear risk of bias in this domain. |
| Incomplete outcome data (attrition bias) | Low risk | Very low attrition. 1 ppt in intervention group didn't respond at 12 weeks, all WLC did. |
| Selective reporting (reporting bias) | Low risk | no protocol, however all measures reported in methods included in results |

Bourgault 2015

| **Methods** | **Title**: Multicomponent Interdisciplinary Group Intervention for Self-Management of Fibromyalgia: A Mixed-Methods Randomized Controlled Trial  Randomised controlled trial - Waiting list control  **Location**: Canada |
| --- | --- |
| **Participants** | **"*Eligibility, Recruitment, and Randomization***  *Subjects were eligible for participation in the study if they: a) were aged 18 years or older, b) were able to read, understand, and complete questionnaires in French, c) had a medical*  *diagnosis of FMS based on the American College of Rheumatology (ACR) classification criteria [23] for at least 6 months, d) reported FMS pain of at least moderate duration (! 4/10) in the seven days prior to enrolment, the FMS pain being the chief complaint if the patient suffered from another chronic pain syndrome, e) were motivated to attend all group sessions and to integrate the proposed self-management strategies, and f) agreed to not introduce new pain medications or other new pain treatment modalities during the 11 weeks of the intervention. Exclusion criteria were the following: a) pregnant or lactating women, b) presence of an active cancer, uncontrolled metabolic disease and other major physical or psychiatric disorder that could compromise patient participation in the study, and d) outstanding litigation regarding patient’s claim for disability payments. Recruitment was conducted through announcements in local newspapers in both study sites between September 2009 and October 2009. Interested subjects were invited to call the re- search coordinator who explained the study, reviewed some of the eligibility criteria, and fixed a first appointment with the potential participants one month prior to the beginning of the intervention. At the time of the first appointment, a pain physician established the FMS diagnosis using the ACR criteria [23], and a physical/psychological evaluation was carried out to ensure the subjects met all the eligibility criteria including proper motivation to partake in the intervention. Written informed consents were obtained from all participants who were then randomly assigned to the Intervention (INT) Group (PASSAGE Program) or the Waitlist (WL) Group. Randomization was stratified by study site and gender, and was done by an independent third party using the Random Allocation Software—Version 1.0.0 (Isfahan, Iran)"*  **Intervention/experimental group**  n=28 (mean age (sd) = 50 (9.23) 93 % female)  **WLC control**  n= 28 (mean age (sd) =48 (11.42) 93% female) |
| **Interventions** | **In/out;** Out  **Intensities**: low  **Group/individual**: Group  I**ntervention/experimental group**  *"the PASSAGE Program is a structured multicomponent interdisciplinary group intervention aimed at reducing FMS symptoms and maintaining optimal function through the use of self-management strategies and patient education. The intervention consists of 9 group sessions with 8 participants lasting 2.5 hours each. As shown in Table 1, each session involved 3 major components—1) psycho-educational tools, 2) CBT-related techniques, and 3) patient-tailored exercise activities. Self-management of the main symptoms of FMS including pain, fatigue, poor sleep quality, and mood fluctuations were targeted during the course of the sessions as well as issues relating to stress management. An additional session was devoted to the pharmacological and non-pharmacological treatments of FMS. The first 8 sessions were held over a period of 11 weeks while the 9th final session was carried out 6 months later to review progress and gain maintenance. The first two sessions were partly devoted to the establishment of a contract with the patient where she/he: 1) fixed three personal outcome goals to be met by the end of the intervention program, 2) determined the minimally acceptable changes to be expected, and 3) agreed to participate in all group sessions and to devote time during the week to the tasks prescribed at the end of each session—i.e., about 45 minutes/day, 6 times/week. Patients were informed that they will be excluded from the program if they missed 2 sessions. The sessions were always conducted in a well-equipped exercise room with mattresses, pil- lows, exercise balls, mirrors, sound system and computer equipment for Power Point presentations. These sessions were interactive and led by two health care professionals who both acted as facilitators, one being mainly responsible for the psychological aspect of the intervention and the other for its physical aspect. Patients were viewed as the “experts” of their condition, and were given a role of active partner in the management of their FMS."*  **Waiting list control**  *"Waitlist (WL) group. Participants randomized to the WL Group were instructed to continue their treatment(s) as usual until they could take part in the PASSAGE Program—i.e., 3 months after the INT Group had completed the program. Changes in pharmacological or non- pharmacological treatments were allowed during this period in the WL Group (usual care*)" |
| **Outcomes** | **Outcomes**:  Pain duration  Fibromyalgia impact questionnaire  Brief pain inventory  Chronic pain sleep inventory  coping strategies questionnaire  beck depression inventory  SF12  Patients global perception of change for: pain, function, quality of life.  **Follow-up time points:** short  pre, post 3 months |
| **Notes** |  |

Risk of bias table

| **Bias** | **Authors' judgement** | **Support for judgement** |
| --- | --- | --- |
| Random sequence generation (selection bias) | Low risk | *"Randomization was stratified by study site and gender, and was done by an independent third party using the Random Allocation Software—Version 1.0.0 (Isfahan, Iran)."* |
| Allocation concealment (selection bias) | Low risk | see comment above. |
| Blinding of participants and personnel (performance bias) | High risk | Not possible due to differences between groups. |
| Blinding of outcome assessment (detection bias) | High risk | The investigator in charge of the statistical analyses (A.L.) was blinded to group assignment. However, as a self report measure is used and no clear blinding of participants from group allocation was possible, there is a high risk of bias in this domain. |
| Incomplete outcome data (attrition bias) | High risk | handling of missing data is mentioned, however no clear explanation given as to what approach was taken. It seems as if the approach was to call ppts if there was missing data on their response sheets. But if patients dropped out of the study or missed a few sessions: *"Patients were informed that they will be excluded from the program if they missed 2 sessions."* |
| Selective reporting (reporting bias) | Low risk | protocol cited and any changes made are justified in publication. |

Cedraschi 2004

| **Methods** | **Title**: Fibromyalgia: a randomised, controlled trial of a treatment programme based on self management  **Location**: *Geneva, Switzerland* |
| --- | --- |
| **Participants** | **Eligible**: *"Participants were volunteers from among patients with FM referred to the divisions of rheumatology and re-education at the Geneva University Hospital. Participation was proposed to 176 consecutive outpatients diagnosed with FM and living in the Geneva area. Recruitment was from November 1998 to September 2000 and follow-up from June 1999 to April 2001. The major inclusion criteria were the American College of Rheumatology criteria for FM1 and sufficient fluency in French to participate in group sessions. Exclusion criteria were the presence of specific medical disorders which required immediate treatment (for example, fractures, infectious diseases), prevented physical activity (for example, cardiovascular problems) or participation in swimming pool sessions (for example, skin diseases, allergy to chlorine). The protocol was approved by the local ethics committee and written informed consent was obtained from all participants."*  total n = 164  **Intervention/experimental group**  n=84. Average age (sd) = 48.9 (9.7), 78% Female  **WL Control**  n= 80. Average age (sd) = 49.8 (9.8), 74% Female |
| **Interventions** | **In/out;** Out  **Duration:** Low (18 hours total approx..)  **Group/individual:** Group.  **Intervention/experimental group**  "*12 sessions, twice a week for 6 weeks. Attendance at >10 sessions was considered full compliance with the programme; 3–9 as partial compliance, and ,3 as withdrawal. Each session lasted 90 minutes (2x45 minutes) and was conducted ingroups of 8–10 people. The programme included swimming pool sessions in 34°C water (8x45 minutes), relaxation exercises (4x45 minutes), low impact land based exercises(2x45 minutes), sessions on activities of daily living (2x90 minutes), and education-discussion sessions (6x45 minutes).*"  **Waiting list control**  "*The control group was offered the treatment programme after the 6 month follow-up evaluation"* |
| **Outcomes** | **Outcomes**;  Psychological General Well- Being (PGWB) index  Short Form-36 (SF-36)  Fibromyalgia Impact Questionnaire (FIQ  regional pain score  treatment satisfaction  **time points**: Intermediate  Baseline, 6 month follow-up. |
| **Notes** |  |

Risk of bias table

| **Bias** | **Authors' judgement** | **Support for judgement** |
| --- | --- | --- |
| Random sequence generation (selection bias) | Low risk | *"After baseline medical evaluation, participants were randomly allocated to a treatment group or a control group."* |
| Allocation concealment (selection bias) | Low risk | *"The assignment was performed in blocks of 20, split into treatment programme (n=10) or control (n=10). Randomisation was made by means of an electronic numbers generator (SPSS). An independent person who was not responsible for determining the participants eligibility provided sequentially numbered, sealed, and opaque envelopes."* |
| Blinding of participants and personnel (performance bias) | High risk | Not possible to blind ppts due to differences between exp groups, both are measured at baseline and 6 months, and participants can be expected to discern whether they have been involved directly in an intervention or whether they have been wating for an intervention. |
| Blinding of outcome assessment (detection bias) | High risk | "i*t was not possible to ensure that the follow-up medical evaluation at 6 months was blind."*  *"coding of the baseline and follow-up self administered questionnaires was blinded*"  As a self report measure is used and no clear blinding of participants from group allocation has been described, there is a high risk of bias in this domain. |
| Incomplete outcome data (attrition bias) | High risk | "*The lack of information on the end points for the drop outs did not allow us to perform the classic intention to treat analysis. The continuous nature of the variables makes it difficult to assign a quantitative value to the missing data. For this reason we analysed the data using an approach close to the per-protocol analysis— that is, using only the cases for which there was information at the end of the follow-up.*" |
| Selective reporting (reporting bias) | High risk | a protocol mentioned but not cited. Some parts of scales used not reported (SF-36 sub scales) |

Dufour 2010

| **Methods** | **Title;** A Randomized, Clinical Trial Comparing Group-Based Multidisciplinary Biopsychosocial Rehabilitation and Intensive Individual Therapist-Assisted Back Muscle Strengthening Exercises  **Location**: Denmark |
| --- | --- |
| **Participants** | **Eligible**: "*Between January 2002 and November 2003, rheumatologists in the county of Copenhagen were invited to refer patients with CLBP to the study site. These patients, together with CLBP patients habitually referred by general practitioners were found to represent the CLBP healthcare population in the area and were consecutively screened. Inclusion criteria were low back pain lasting more than 12 weeks with or without pain radiating into the leg(s), and an age of 18 to 60 years. Patients were evaluated by conventional radiograph, CT scan, or MRI scan of the lumbar spine, as well as by physical examination. Patients with symptoms of serious spinal pathology such as malignancy, osteoporosis, vertebral fracture, spinal stenosis, clinical symptoms of an acute herniated disc accompanied by nerve root entrapment, unstable spondylolisthesis, spondylitis, health conditions that prevented them from performing strenuous exercise and language problems were excluded.*"  **Total sample before randomisation: n=286. IMPT=142 Control n=144** baseline data below represents number that proceeded to begin treatment  **MBI**  Baseline: n=129 (mean age (sd) = 41.2(10) 56.6%female  3month FU n= 118  6month FU n=107  12 month FU n=92  24 month FU n=83  **Control -** Intensive Individual Therapist-Assisted Back Muscle Strengthening Exercises  Baseline: n=143 (mean age (sd) = 40.6 (9.1) 55.9 %female)  3month FU n= 132  6month FU n= 122  12 month FU n=107  24 month FU n=91 |
| **Interventions** | **In/out:** out  **Duration:** med, 75 hours in total:  **group/individual:** mixed  **MBI**: Intervention was biopsychosocial group intervention:  "*Patients in group A were treated in groups of 6 patients and received a program of combined exercise, education, and pain management based on a program described by Bendix et al.24,25,27–29 The patients were reassured that there was no serious cause for their back pain and that the exercise program was safe and effective. Treatment was scheduled for 12 weeks and divided into 3 periods of 4 weeks. During the first period, exercise was performed 3 times a week in 2-hour sessions. At the first session, a preprogram assessment was performed to familiarize patients with the exercise program, set treatment goals, and set the initial duration for each exercise. The following sessions began with a warm-up and ended with stretching. The bulk of the session consisted of aerobic training and training to strengthen the muscles in the back, gluteus region, and abdominal wall. These exercises were all performed in the supine position using machines and circuit training. In total, 22 hours of exercises were performed. In addition, patients were provided 1.5 hours to play ball games, 1.5 hours of training in hot water, and 2 hours of ball stick training. Biweekly lessons on anatomy, postural techniques, and pain management were provided by a physiotherapist and on back care and lifting techniques by an occupational therapist in total 10 hours. During the second period, 2-hour exercise sessions were performed twice a week at the study site and once a week at either the patient’s home or in a fitness center. During the third period, 2-hour exercise sessions were performed 3 times a week at home or in a fitness center. In total, the patients performed 75 hours of moderate muscle training exercise."*  **Control**: **I**ntensive Individual Therapist-Assisted Back Muscle Strengthening Exercises  "*Patients in group B received a program of specific and in- tensive muscle training exercises to strengthen and shorten the muscles in the back and gluteus region developed by Oefeldt.41 The program consisted primarily of body and leg lifting in the prone position, supplemented with exercises aimed at dynamic contraction of painful muscles. The program did not include stretching or abdominal muscle exercises. The body and leg lifting exercises were carried out in 6 sets of 10 repetitions, and exercises involving musculus piriformis were executed in 3 to 6 sets of 15 repetitions. All other exercises were carried out in 3 to 4 sets of 10 repetitions. Each patient had a specially trained therapist who encouraged and assisted the patient in order to achieve full contraction of the painful muscle and who arranged the patient in the least painful position using different pillows and straps together with suitable equipment.42 Initially, the therapist did most of the work, but gradually reduced the amount of assistance such that the patient took over progressively more of the work. The program ran for 1 hour twice a week for 12 weeks and included a physical examination at the beginning and at the end. In total, the patients received 22 hours of intensive muscle training exercise. The treatment- related costs per patient amounted to 24 hours of therapist assistance."* |
| **Outcomes** | **Outcomes**:  Roland Morris Disability questionnaire  SF-36  VAS pain scale  Global perceived outcomes  Ability to work  **Timepoints**: (short, med, long)  Baseline, 3, 6, 12, +24 month follow-up |
| **Notes** |  |

Risk of bias table

| **Bias** | **Authors' judgement** | **Support for judgement** |
| --- | --- | --- |
| Random sequence generation (selection bias) | Low risk | "*...they were allocated by an separate secretary to a group-based multidisciplinary biopsychosocial rehabilitation programme (group a) or intensive individually therapist assisted... according to random number chart"* |
| Allocation concealment (selection bias) | Low risk | see quote above |
| Blinding of participants and personnel (performance bias) | Unclear risk | There was a single researcher who was blind: "*All physical examinations at trial visits were performed by one physician who was blinded to the treatment group and had no access to the treatment areas."*  Unclear whether PPTs were blind; "*The groups were treated at separate locations and had no personal contact during treatment*"  depending on the level of detail provided for informed consent, it is possible that participants could have been unaware of their group allocation. |
| Blinding of outcome assessment (detection bias) | Unclear risk | researcher gathering measurements from groups was blind, however no mention of blinding of analysis. However, as a self report measure is used to assess HRQoL, and no clear blinding of participants from group allocation has been described, there is an unclear risk of bias in this domain. |
| Incomplete outcome data (attrition bias) | Low risk | intention to treat applied |
| Selective reporting (reporting bias) | Low risk | No protocol, but all measures noted in methods reported in results section. |

Dysvik 2010

| **Methods** | **Title:** The effectiveness of a multidisciplinary pain management programme managing chronic pain on pain perceptions, health-related quality of life and stages of change—A non-randomized controlled study  Non-randomised quazi-experimental control study  **Location:** Norway |
| --- | --- |
| **Participants** | **Eligibility/Recruitment**; "*A consecutive sample of 117 outpatients from 11 treatment groups referred to a rehabilitation unit was included in this intervention study. Four patients did not complete the programme, reducing the sample to 113. Participants from every second group were defined as waiting list controls during the 8-week basic course, and out of these, 39 formed the control group. After the control period they were included in the 113 final participants. A total of 48 participants selected at random received an ‘‘additional package’’, containing a contract to be signed and two individual follow-ups. The participants were recruited through their general practitioners and were considered a representative sample for this purpose. They met the following inclusion criteria for admission:*  *aged between 18 and 67 years*  *chronic non-malignant pain lasting for longer than 6 months*  *medical investigation and/or treatment completed before referral*  *motivated to participate in an active rehabilitation programme*  *no ongoing litigation due to their pain problem Exclusion criteria:*  *affected by major mental disorders*  *affected by major medical conditions requiring treat- ment*  *Prior to inclusion, all participants were given an introduction day where the programme, expectations and obligations were discussed. In addition, a clinical interview was performed by one of the counsellors to assess suitability. Written instructions were handed out. It was emphasized that participation was voluntary and that they could leave the programme at any time. Confidentiality was guaranteed and a written consent was obtained*"  **Total Sample**: n=117 (79% female, age mean (range); 45 (21–66))  **Experimental/Intervention group:**  n=78 (79% female, mean age (range): 45 (21–66)  **WL control group**.  n=39 (77% female, mean age (range): 44 (22–64)  "*Participants from every second group were defined as waiting list controls during the 8-week basic course, and out of these, 39 formed the control group. After the control period they were included in the 113 final participant"* |
| **Interventions** | **Inpatient vs outpatient:** out  **Intensities**: **med** 40 hours approx. (5 h per week for eight weeks)  **Group or individual**: group  **intervention**:  *"is based on a group approach and consists of therapeutic dialogues and training, combined with physical activity and associated homework"*  "*The original CBT programme described elsewhere is based on a group approach and consists of therapeutic dialogues and training, combined with physical activity and associated homework. Basic assumptions are acceptance of the chronic pain diagnosis, understanding of the body-mind connection and an active orientation towards self-management. Furthermore, the programme has several features that directly address the psychosocial aspects in chronic pain, which are further specified*  *The main changes in the treatment programme made for the present study were:*  *-A 1-day introduction before initiation*  *-Inclusion in the team of a volunteer patient who has gone through the programme and who has been trained to provide some counselling, in addition to the two counsellors*  *-Extending each meeting from 3 to 5 h, with a lunch break included*  *- In addition to the core programme, development of an additional package administered at random, which included two individual consultations and signing a contract.*  *Each group met with the two counsellors (a nurse and a physiotherapist) and the volunteer for 5 h a week over an 8- week period. A psychologist and a physician also took part in the training.*"  **Waiting list controls**  *"Data were routinely collected before starting (time t1) and after termination of the course (time t2). For the waiting list controls, data were also collected 8 weeks before start (time t0), and the time span from t0 to t1 was approximately the same as from t1 to t2. Demographic data were collected at t1."* |
| **Outcomes** | **Outcomes**:  Brief pain inventory  pain stages of change questionnaire  patient satisfaction  SF-36  **time points**: (short)  pre post (8 weeks) |
| **Notes** |  |

Risk of bias table

| **Bias** | **Authors' judgement** | **Support for judgement** |
| --- | --- | --- |
| Random sequence generation (selection bias) | Unclear risk | "*Participants from every second group were defined as waiting list controls during the 8-week basic course*" - detail lacking, could be considered quasi-randomised, however title specifically labels the study 'non-randomized' |
| Allocation concealment (selection bias) | High risk | Not expressly detailed but based on information from the randomisation process means that we can deduce this. |
| Blinding of participants and personnel (performance bias) | High risk | not possible due to differences between exp and control condition. |
| Blinding of outcome assessment (detection bias) | High risk | As a self report measure is used to assess HRQoL, and no clear blinding of participants from group allocation has been described, there is a high risk of bias in this domain. |
| Incomplete outcome data (attrition bias) | High risk | information not included on how drop out data was managed |
| Selective reporting (reporting bias) | Low risk | No protocol, but all measures noted in methods reported in results section. |

Gatchel 2009

| **Methods** | **Title:** Preliminary Findings of a Randomized Controlled Trial of an Interdisciplinary Military Pain Program  Randomized Controlled Trial  **Location**: USA |
| --- | --- |
| **Participants** | **recruitment**; *"After signing an informed consent document, a total of 66 participants were assessed individually to determine a pre- treatment baseline for all measures. They were then randomly assigned to either one of two treatment groups: (1) Standard Treatment Comparison Group (standard anesthesia pain clinic medical care; n = 36) or (2) Functional Restoration (n = 30). The two groups were matched for age, gender, race, and time since original injury or onset of pain, on the basis of an urn randomization procedure*"  **Functional restoration**  Baseline: n=30 (mean age (SD)=36.9 (7.5). 70% Male)  6 months: n=22 ST  **Standard treatment**  Baseline: n= 36 (mean age (SD)=34.4 (6.9). 64 % Male)  6 month n=23 |
| **Interventions** | **In/out**: Information not included  **Duration**: information unclear  **Individual vs group**: Information not included  **Functional restoration**  "*The functional restoration approach involves an interdisciplinary team approach consisting of three major com- ponents—physical therapy, occupational therapy, and psycho- social intervention—which is guided by a supervising nurse and physician team. It is based upon the assumption that almost all patients suffering from occupational musculoskeletal pain and disability can be returned to a productive life- style through appropriate reconditioning and coping skills training. This program is accomplished through an aggressive psychosocial and physical reconditioning program, not through traditional passive physical treatment modalities. Treatment is initially guided by quantified measurements of function, which not only allows the reconditioning to proceed safely, but also provides quantifiable documentation of compliance, effort, and eventual success. Psychosocial issues and return-to-work issues are simultaneously addressed by the psychology and occupational therapy components of the program. Such issues can be effectively dealt with using psychosocial approaches.8 We have described this approach in detail in a number of publications*." (9,10 on source reference list) - see notes section below for futher consideration of this.  **ST Comparison Group**  "*The ST Comparison Group (standard treatment in the anesthesia pain clinic) received treatment similar to specialty pain treatment available at many of the larger military medical treatment facilities with staffing commensurate to that of WHMC. This treatment is more than the usual medical care that most patients with chronic musculoskeletal pain condi- tions receive by their primary medical provider or primary care manager. Physicians working at WHMC and BAMC are anesthesiologists with fellowship training in pain management or pain medicine. Of the five providers in these locations, all were board certified in anesthesiology, four were board certified in pain management, and the other was board eligible in pain management. These providers had extensive experience in the assessment and treatment of chronic pain conditions, including musculoskeletal pain disorders. Common treatments in this setting include management of pain medications, proper use of antidepressant medications as appropriate, nerve blocks and steroid injections, and a basic exercise program when appropriate."* |
| **Outcomes** | **Outcomes**:  Pain Drawing and Visual Analog Scale (VAS)  Dallas Pain and Disability Questionnaire  Oswestry Disability Questionnaire  SF-36  Beck Depression Inventory  Multidimensional Pain Inventory  Fear Avoidance Beliefs questionnaire  Functional Capacity evaluation  **Timepoints** (med, long)  Pre, Post, 6 month FU, 12 month FU |
| **Notes** | Military study.  Gaps in key pieces of information which were unable to be recovered, particularly regarding specifics of the actual intervention. As quoted in the interventions section above, the authors direct the reader to other sources for detail on the intervention specifics, however in both cases the sources cited are somewhat dated general books on the functional restoration treatment approach broadly:  Gatchel RJ, Turk DC: Psychological Approaches to Pain Management: A Practitioner’s Handbook. New York, Guilford Publications, Inc., 1996.  Mayer TG, Gatchel RJ: Functional Restoration for Spinal Disorders: The Sports Medicine Approach. Philadelphia, PA, Lea & Febiger,1988. |

Risk of bias table

| **Bias** | **Authors' judgement** | **Support for judgement** |
| --- | --- | --- |
| Random sequence generation (selection bias) | Low risk | *"The two groups were matched for age, gender, race, and time since original injury or onset of pain, on the basis of an urn randomization procedure"* |
| Allocation concealment (selection bias) | Low risk | *"An independent individual, who was not responsible for determining the eligibility of patients for the study, was responsible for the randomization assignment"* |
| Blinding of participants and personnel (performance bias) | Unclear risk | Information not included |
| Blinding of outcome assessment (detection bias) | Unclear risk | Information not included |
| Incomplete outcome data (attrition bias) | High risk | No discussion of how drop out data handled. Data reported in a per protocol style. |
| Selective reporting (reporting bias) | Low risk | All materials from method reported in results. |

Grahn 1998

| **Methods** | **Title:** Effects of a multidisciplinary rehabilitation programme on health-related quality of life in patients with prolonged musculoskeletal disorders: A 6-month follow-up of a prospective controlled study  6 month follow-up of a prospective controlled study.  **Location:** Sweden |
| --- | --- |
| **Participants** | **Eligibility/recruitment**: *"In 1994 the county of Kronoberg had a total population of about 181000, for which the Kronoberg Occupational Rehabilitation Service has regional responsibility. Over a period of 1.5 years all the patients consecutively referred to this centre who fulfilled the inclusion criteria were invited to participate in the study. The inclusion criteria were: PMSD as main diagnosis (such as cervical syndromes, lumbago and sciatica including pain and movement limitations, chronic pain syndromes), problems with long and/or repeated short periods of sick leave during the past year and rehabilitation periods in 1994and/or the first half of 1995. The exclusion criteria were: temporary or permanent complete disability pension, known substance abuse, serious mental illness or being a non-Swedish speaker. In all, 129 patients were invited to participate in the study and 122 accepted. A control group of patients matched with respect to PMSD, sex, age, cultural background, employment/unemployment and the extent of sick leave was identified by the Regional Social Insurance Office in the county of Kronoberg. A total of 114control patients accepted the invitation to participate."*  **Exp**  baseline: n= 122 (mean age (SD) n= 44.3 (9.1) 82% Female  6m follow-up n=115  **Control**  baseline: n= 114 (mean age (SD) n= 44.8 (9.2) 44.3 (9.1) 82.5% Female  6m follow-up n=107 |
| **Interventions** | **In/out:** in (for exp group)  **Duration**: high  **Group/individual:** mixed  "*All the patients in both groups had their own general practitioner and access to paramedical care (mostly physiotherapy) at a primary health-care centre."*  **exp**  "*The inpatient rehabilitation programme relies on a bio-psychosocial/system-theoretical approach and focuses on BAT and cognitive and relaxation treatment. The programme had no special gender perspective.’ The aim of the team of health-care providers was thus to frame an individually designed rehabilitation programme in which the patient was active in setting objectives related to work, leisure and social pursuits, in both the longer and shorter term. The patient was given an individual schedule (see Appendix). The programme continued for 4 weeks, including workplace visits, and was followed by an active 6-month follow-up period, in which the patients could contact the team at any time for further advice. At least two visits with the team after 3 and 6 months were scheduled during the follow-up period. The multidisciplinary rehabilitation teams consisted of a chief senior rehabilitation physician, consultant specialists in orthopaedics and psychiatry, a physiotherapist, occupational therapist, social adviser and assistant nurse."*  Duration estimation: appendix provides a schedule for week 2 showing a 8am -4pm 5 day a week plan. this is reported to continue for 4 weeks. 160 hours approx.  **control**:  Patients received standard care in the Swedish healthcare system: "*In patients with PMSD, traditional treatment within primary health care usually consists of a medical examination, advice, prescription of medicine, assessment of the need for sick leave and a referral for physiotherapy. This treatment usually involves a physical examination, mobility and strength training, stretching of tight muscles and advice on a home-training programme."* |
| **Outcomes** | **Outcomes**:  Nottingham Health Profile  Body awareness scale  postural control test  pain-related drug consumption  Assessment of pain  Isometric endurance test  Aerobic capacity  questionnaire about health and work environments  **Time points**: (med)  Baseline, 6 month follow-up. |
| **Notes** | there was on going follow-up treatment after the initial intervention  inpatient setting |

Risk of bias table

| **Bias** | **Authors' judgement** | **Support for judgement** |
| --- | --- | --- |
| Random sequence generation (selection bias) | High risk | Unclear on the process. Paper says, 122 people accepted the invitation to participate in exp group, and then an age and gender matched control group was recruited |
| Allocation concealment (selection bias) | High risk | no, see previous comment |
| Blinding of participants and personnel (performance bias) | High risk | It would not possible to blind participants due to difference between conditions (effectively passive control group, just receiving ongoing standard treatment), no information provided about blinding of investigators |
| Blinding of outcome assessment (detection bias) | High risk | Due to lack of blinding of participants and self evaluation of outcomes, this category has been rated as high. |
| Incomplete outcome data (attrition bias) | Low risk | intention to treat applied |
| Selective reporting (reporting bias) | Low risk | no protocol, measures reported in method included in methods reported in results. |

Grahn 2000

| **Methods** | **Title**: Motivation as a predictor of changes in quality of life and working ability in multidisciplinary rehabilitation  prospective 2 year controlled study. Carried out in Sweden between 94-96. (follow-up of Grahn 1998)  **Location**: Sweden |
| --- | --- |
| **Participants** | **Eligibility/recruitment:** The inclusion criteria for invited patients were: prolonged MSD as the main diagnosis (such as cervical syndromes, lumbago and sciatica including pain and movement limitations, chronic pain syndromes), problems with long and}or repeated short periods of sick leave during the past year and rehabilitation periods in 1994 and}or the first half of 1995. The exclusion criteria were : temporary or permanent complete disability pension, known substance abuse, serious mental illness or being a non-Swedish speaker. A total of 122 patients agreed to participate. The control group was matched in terms of prolonged MSD, sex, age, cultural background, employment}unemployment and the extent of sick leave  **exp**:  n= 122 (female=82%) mean age 44±3 years(SD9±1)  **Control**  n=114 (female= 82%) mean age 44±8 years(SD 9±2)  (control group was matched in terms of prolonged MSD, sex , age, cultural background, employment} unemployment and the extent of sick leave. |
| **Interventions** | **inpatient vs outpatient**: In  **Intervention intensities:** high, Approx 40 hours a week individual tailored intervention (detail provided in Grahn 1998)  **Group or individual approach**: mixed.  **Exp**:  multidisciplinary rehabilitation teams consisted of a chief senior rehabilitation physician, consultant specialists in orthopaedics and psychiatry, a physiotherapist, occupational therapist, social adviser and assistant nurse. individually tailored biopsychosocial intervention. The rehabilitation programme continued for 4 weeks, including workplace visits, and was followed by an active 6-month follow-up period, in which the patients could contact the team at any time for further advice.  **Control**:  Received standard treatment for the Swedish medial system: '*Traditional treatment within primary care for patients with prolonged MSDs generally includes a medical examination, advice, prescription of medicine, assessment of the need for sick leave and a referral for physiotherapy, such as heat, massage, mobility and strength training, stretching of tight muscles and home- training advice. The content of the standard treatment was checked by general practitioners and district physio- therapists within the region. At the time of the present study, BAT was not accessible within traditional physio- therapeutic treatment in the region."* |
| **Outcomes** | **Outcomes:**  Nottingham Health profile (HRQoL)  Body awareness scale  Pain: VAS  isometric endurance test  Aerobic endurance  health and working env. questionnaire  psychosomatic symptoms  working ability  motivational analysis  **Time points**:(long-term).  baseline and 24 month follow-up |
| **Notes** |  |

Risk of bias table

| **Bias** | **Authors' judgement** | **Support for judgement** |
| --- | --- | --- |
| Random sequence generation (selection bias) | High risk | For organizational and ethical reasons, a randomized design could not be used in the present study. |
| Allocation concealment (selection bias) | Unclear risk | information not included |
| Blinding of participants and personnel (performance bias) | High risk | not possible (passive control) |
| Blinding of outcome assessment (detection bias) | High risk | information not included, however, as a self report measure is used to assess HRQoL, and no clear blinding of participants from group allocation has been possible, there is an high risk of bias in this domain. |
| Incomplete outcome data (attrition bias) | Low risk | Intention to treat approach taken |
| Selective reporting (reporting bias) | Low risk | no protocol, all measures from methods reported in results. |

Helminen 2015

| **Methods** | **Title**: Effectiveness of a cognitive– behavioural group intervention for knee osteoarthritis pain: a randomized controlled trial  **Location**: Finland |
| --- | --- |
| **Participants** | **Eligibility/recruitment: *"****Patients aged between35and75yearswithclinical symptoms and radiographic grading (Kellgren– Lawrence 2–4)18 of knee osteoarthritis were eligible. All patients had experienced pain within the last year in or around the knee occurring on most days for at least a month.19 One week prior to the study, their knee pain was rated as ≥40 on a 100- mm visual analogue scale in the pain subscale of the Western Ontario and McMaster Universities (WOMAC) Osteoarthritis index.20 Exclusion criteria encompassed severe psychiatric or psychological disorders that had led to hospitalization or an inability to work, previous or planned lower extremity joint surgery, and other back or lower*"  **Exp**:  (GP care + intervention, n =55) (71% female) Mean age (SD): 64.5 (7.3)  **Control"** (GP care, n = 56) (68% female) Mean age (SD): 62.8 (7.2) |
| **Interventions** | i**npatient vs outpatient:** out  **Intervetnion intensities:** low  **Group or individual approach**: mixed.  **Exp**:  "*Patients allocated to the cognitive–behavioural group intervention took part in six weekly sessions supervised by an experienced psychologist and a physiotherapist. The sessions took place in a group of 7−13 persons according to the model presented by Linton.14 Each session lasted for two hours with a 15−20 minute break to enhance peer support and social bonding. The outline of the sessions included an introduction (15minutes), lecture (knowledge and insight, max 15minutes), problem solving (in pairs/teams, 15−20minutes), skills training (15−20minutes), homework assignments (15min- utes), and a résumé (feedback) of the session (15 minutes). A written example of a knee osteoar- thritis pain patient’s life was used throughout the intervention as a basis for discussion and practice in problem solving. An outline of each session is presented elsewhere.17 The psychologist was the principal leader of the cognitive–behavioural intervention. The physiotherapist’s tasks were to lead the relaxation exercises, provide the information on osteoarthritis pain mechanisms in the first session, offer advice about suitable exercises in the second session, and facilitate the group in general when needed. Both the intervention and the control group continued side by side with the usual GP care that patients might have received in primary care throughout the study*."  Control:  Received standard Finnish GP care |
| **Outcomes** | **Outcomes**:  WOMAC self reported pain scale  Örebro Musculoskeletal Pain Questionnaire  pain - numeric rating scale  Treatment use was recorded  Life satisfaction,  sense of coherence, pain self-efficacy,  kinesiophobia,  pain catastrophizing.  beck depression inventory  global assessment of change  15d euroqol  SF-36  **Timepoints**: (short, long)  baseline, 3 month, 12 month |
| **Notes** |  |

Risk of bias table

| **Bias** | **Authors' judgement** | **Support for judgement** |
| --- | --- | --- |
| Random sequence generation (selection bias) | Low risk | "the participants were randomly assigned to intervention" |
| Allocation concealment (selection bias) | Low risk | The study doctors responsible for enrolling the patients, distributing the envelopes for randomization, collecting the data, and performing the statistical analysis were blinded to the group assignment and were not involved in providing the interventions. |
| Blinding of participants and personnel (performance bias) | High risk | Only one group receives active intervention so not possible to blind participants to group allocation |
| Blinding of outcome assessment (detection bias) | High risk | study doctors responsible for the collection were blinded to the group assignment. However as a self report measure is used to assess HRQoL, and no clear blinding of participants from group allocation has been described therefore a high risk of bias has been estimated in this category |
| Incomplete outcome data (attrition bias) | Low risk | "All analyses adhered to the intent-to-treat principle." |
| Selective reporting (reporting bias) | Low risk | study adhered to protocol outline |

Hurley 2007

| **Methods** | **Title**: Clinical Effectiveness of a Rehabilitation Program Integrating Exercise, Self-Management, and Active Coping Strategies for Chronic Knee Pain: A Cluster Randomized Trial  **Location**: England |
| --- | --- |
| **Participants** | **Eligibility/recruitment:**  recruited individuals age 50 years or older who had consulted a primary care physician for mild, moderate, or severe knee pain of 6 months’ duration.  **total** n= 418  **individual rehab**  n = 146 (6 weeks n=127) (6 months n=120) (*see note below about collapsing of goups*)  **group rehab**  n =132 ( 6 weeks n= 111) ( 6 months n=107) age: 66 (50–91) 68 (51–84) 85%female  **usual care:**  n=140 (6 weeks n= 127) (6 months n=110) Mean age: 67 (51–89) 69% female |
| **Interventions** | **In**/**out**: Out  **Group**/**individual**: group  **Duration**: low  pragmatic study compared usual primary care with a rehabilitation program. The content and format were identical for both Indiv-rehab and Grp-rehab, involving 12 supervised sessions (twice weekly for 6 weeks) that combined discussion on specific topics regarding self-management and coping, etc., with an individualized, progressive exercise regimen.  **Exp:**  The intervention comprised integrated patient education, with simple self-management and pain coping strategies, delivered in the first 15---20 minutes of each rehabilitation session. Sessions were designed to be interactive, including active problem solving where appropriate. The content and progression were similar irrespective of participation in individual rehabilitation or group rehabilitation. This was followed by a 35---45-minute individualized progressive exercise program. The order in which the exercises were performed varied due to the circuitous regimen, and exercise specificity varied between participants and within participants over time, depending on their ability, rate of progression, and identified areas of disability. Exercise complexity and duration was increased through mutual agreement between physiotherapist and participant.  **Usual Care**: |
| **Outcomes** | **Outcomes**  western Ontarioand McMaster Universities Osteoarthritis Index (WOMAC)  objective functional performance (aggregated functional performance time of 4 common activities of daily living exercise health beliefs and self-efficacy questionnaire  hads  EuroQol  patient preference health-related quality of life questionnaire (McMaster Toronto Arthritis [MACTAR])  quadriceps strength (quadriceps maximum voluntary contraction), and quadriceps voluntary activation (29).  **time points**: (Short, long)  baseline, 6 weeks, 6 months |
| **Notes** | At the start of this paper, they begin by presenting data for 3 groups: control, individual rehab, group rehab. However, by the time they are reporting the data, they have collapsed the two real groups into a single group for analysis. Due to this we are just using the combined effect size as the main finding, and so there are only 2 groups shown in our data. The control and a combined rehab group. |

Risk of bias table

| **Bias** | **Authors' judgement** | **Support for judgement** |
| --- | --- | --- |
| Random sequence generation (selection bias) | Low risk | *"Primary care practices were the unit of randomization. The randomization list was generated at a central location away from the research center by an author (BR), who was not involved in the execution of the trial."* |
| Allocation concealment (selection bias) | Low risk | "*Practices were randomly allocated in blocks of 3 to receive 1) usual primary care (whatever intervention a participant’s primary care physician considered to be re- quired and appropriate), 2) usual primary care plus indi- vidual rehabilitation (Indiv-rehab), or 3) usual primary care plus rehabilitation in groups of 8 participants (Grp- rehab)*." |
| Blinding of participants and personnel (performance bias) | High risk | not possible due to difference between groups |
| Blinding of outcome assessment (detection bias) | High risk | "Outcome assessors were blinded to a participant’s allocation". However, as a self report measure is used to assess HRQoL, and no clear blinding of participants from group allocation has been possible, there is a high risk of bias in this domain. |
| Incomplete outcome data (attrition bias) | Low risk | "*prespecified protocol, based on intent-to-treat with no interim or post hoc analyses."* |
| Selective reporting (reporting bias) | Low risk | protocol is mentioned in text but not cited, all measures from methods reported in results. |

Jensen 2001

| **Methods** | **Title**: A randomized controlled component analysis of a behavioral medicine rehabilitation program for chronic spinal pain: are the effects dependent on gender?  **Location**: Sweden |
| --- | --- |
| **Participants** | **Eligibility/recruitment:**  "T*he subjects were 214 individuals (97 men, 117 women) suffering from long-term, non-specific spinal pain. Data was gathered between May 1995 and October 1999. The selected rehabilitation clinics were situated in Stockholm, Gothenburg, Helsingborg, and Malm?.The inclusion criteria were as follows: non-specific spinal pain, currently and continuously sick-listed for at least 1 month and a maximum of 6 months before inclusion (because of spinal pain), fluency in Swedish and 18-60 years of age. Exclusion criteria were: serious spinal pathology (e.g. tumors or spinal fractures), exposure to physical trauma within 6 months of examination (e.g. a whiplash associated disorder), objective neurological signs indicating a need for surgery, comorbidities (e.g. alcohol abuse, acute psychosis), ongoing rehabilitation, and verified pregnancy. The source population were persons covered by the AGS insurance scheme, which is a health insurance plan covering 2.4 million employees in Sweden. (AGS is a sick pay and disability pension insurance scheme which is part of the agreement between the Swedish Employers' Confederation (SAF) and the Swedish Trade Union Confederation (LO)). The recruitment procedure can be divided into four steps."*  total n= 214 (mean age (sd) = 43.3 (10.4) 55 % female)  Baseline:  **Physical Therapy**  1./ n=54 (mean age (sd) = 43.3 (9.4) 68 % female)  **Cognitive Behavioural**  2./ n=49 (mean age (sd) = 43.8 (9.6) 45 % female)  **Behavioural Medicine**  3./ n=63 (mean age (sd) = 42.5 (11.8) 48 % female)  **Control Group**  4./ n=48 (mean age (sd) = 43.9 (10.8) 58 % female) |
| **Interventions** | **In/out:** out  **intensities:** high: Interventions took place over a 4 week period. Groups 1 and 2 were aprox. 20 hours a week (80 hours total). group 4 was 'full time' (specific number of hours not provided, however on the assumption that full time implies a 9-4pm schedule this would equate to around 35 hours a week therefore 140 hours total).  **individual/group:** Group  **4 groups**:  1./**physical therapy (PT)**  "*The PT intervention was carried out on a part-time basis (approximately 20 scheduled hours per week) and was aimed at enhancing the physical functioning and facilitate a lasting behavior change of the individual. Each participant was assigned to an individually tailored training program. The PT intervention involved a pedagogical approach in that the participants were given practical examples of how to perform different activities in their everyday lives which they had reported to be problematic. The program included individual goal setting, gradually increased exercises to improve muscular endurance, aerobic training (e.g. cycling on a test bicycle), water exercise (pool training), relaxation techniques according to Jacobson (1938) and Westin (1985) and body awareness therapy. Practical sessions in ergonomics were also included as well as didactic presentations of anatomy and exercise physiology. Home work assignments for physical activities were given according to the individual's interests and problem areas.*"  2./ **CBT**  "*The CBT intervention comprised, on average, 13-14 scheduled hours per week and was aimed at improving the subjects ability to manage their pain and to resume a normal level of activity. If no other literature references are given, the techniques referred to in the following have been described in Philips (1988) and/or Turk et al. (1983). The CBT program included activity planning and goal setting, problem solving, applied relaxation (Lisspers and Hallgren, unpublished data, 1994), cognitive coping techniques (e.g. distracting imagery, external focusing, coping self-statements), activity pacing, the role of vicious circles and how to break them, the role of significant others and assertion training. Individually tailored homework assignments were given at the end of each session pertaining to what had been dealt with during the session. The general structure of the sessions was as follows: discussion of the homework assignments given at the last session, introduction of a new topic or a new step in the treatment, a practical example of the topic, and, finally, new homework assignments*."  **3./behavioural medicine (PT +CBT)**  "*This condition included both the PT and CBT programs."*  **4./Treatment as usual.**  *"The CG was not offered any types of intervention in the research project. Consequently, they were subjected to the normal routines in health care."* |
| **Outcomes** | **Outcomes:**  Sick leave from work  early retirement  SF-36  **Timepoints: (med, long)**  Pre, post, 6month, 18 month follow-up |
| **Notes** | This study reports that it creates a 'global' score for the SF-36 by combining the mean of the sub scales. As far as understood this is not a valid approach. Individual sub-scales are still reported however data is split by gender. (while the actual figures are reported for individual sub-scales, the statistical analysis lumps them all together). - Following consultation from DS and FM; recommend treating the two different group s as different samples.  All participants in the study were members of a specific work based insurance scheme in Sweden.  Some large gender imbalances between groups  This study has 4 groups. 1 is IMPT intervention, then 2 active controls and one TAU. Can't do multiple comparisons between IMPT and both active controls, so have opted to make a comparison between IMPT and PT (as PT has larger sample size) and omit CBT group data from the comparisons. |

Risk of bias table

| **Bias** | **Authors' judgement** | **Support for judgement** |
| --- | --- | --- |
| Random sequence generation (selection bias) | Low risk | *"a block randomization procedure was employed to ensure an even distribution of treatment conditions"* |
| Allocation concealment (selection bias) | Low risk | *"the randomisation was carried out with the use of opaque envelopes, each envelope containing one of the conditions on it."* |
| Blinding of participants and personnel (performance bias) | High risk | Due to differences in conditions it was not possible to blind either participants or personnel. |
| Blinding of outcome assessment (detection bias) | High risk | information not included. However, as a self report measure is used to assess HRQoL, and no clear blinding of participants from group allocation has been possible, there is an unclear risk of bias in this domain. |
| Incomplete outcome data (attrition bias) | Low risk | Intention to treat analysis |
| Selective reporting (reporting bias) | Low risk | No protocol, all measures from methods reported in results. |

Jensen 2011

| **Methods** | **TItle**: One-Year Follow-Up in Employees Sick-Listed Because of Low Back Pain: Randomized Clinical Trial Comparing Multidisciplinary and Brief Intervention  **Location**: Denmark |
| --- | --- |
| **Participants** | **Eligibility/recruitment**  The general practitioners were encouraged to refer patients to the study at the Research Unit of the Spine Center, Regional Hospital Silkeborg, Denmark, if the patients were aged 16 to 60 years and partly or fully sick-listed from work for 4 to 12 weeks because of LBP. The first visit at the Spine Center was not always possible within this time frame, and consequently the duration of sick leave ranged from 3 to 16 weeks at the time of inclusion. The patients were not en- rolled in the study if they1 were unemployed,2 had continuing or progressive signs or symptoms of nerve root affection im- plicating plans for surgery,3 had low back surgery within the last year or specific back diseases, (e.g., tumor),4 were pregnant,5 had known dependency on drugs or alcohol or6 had any primary psychiatric disease.  **Exp**  n = 175 (mean age 42.1 (10.5)) 54% female  **Control**  n= 176 (mean age 41.9 (10.4)) 50.3% female |
| **Interventions** | **Duration:** unclear (further information requested but no reply reieved) " *A more detailed description of the interventions can be found at our homepage*." - no homepage found.  **In/out**: Out  **Group/indiviual:** Mixed  **Control**:  A standard clinical LBP examination was carried out by the physician, relevant imaging and examinations were ordered and treatment options were discussed. Patients with nonspecific LBP were informed about the difficulties of visualizing the cause of pain with certainty, the best documented treatment being exercise and training, and psychological distress possibly worsening and prolonging pain. Patients with nerve root pain were informed about the good spontaneous prognosis and about the possibility of surgery if no improvement occurred. Furthermore, they were informed about exercise being beneficial if leg pain did not worsen. Information was given in a reassuring way and medical pain management was adjusted. The participants were advised to resume work when possible. The physiotherapy examination included a standardized, mechanical evaluation, and advice on exercise was chosen accordingly. General advice was given to increase physical activity and exercise. To ensure coordination be- tween stakeholders copies of the medical records were always sent to the participant, the general practitioner, and the municipal social services responsible for reimbursement of sick leave compensation. For all participants, a follow-up visit at the physiotherapist was scheduled 2 weeks later, and a follow- up visit at the physician was arranged for participants needing answers in relation to test results  **Experimental:**  In addition to the brief clinical intervention described above, participants allocated to the multidisciplinary intervention group were scheduled for an interview with a case manager within two to three workdays. This interview was standardized and included questions of work history, private life, and questions on how pain and disability was perceived. It normally lasted for 1 to 2 hours. The participant was seen once or more times by the case manager depending on need and progress. The case manager and the participant together made a tailored rehabilitation plan aiming at full or partial RTW. If this was deemed unrealistic, a plan toward staying on the labor market in other ways was made, for instance by jobs supported by the social system. Each case was discussed several times by the entire multidisciplinary team including the rehabilitation physician, a specialist in clinical social medicine, a physiotherapist, a social worker, and an occupational therapist. Appointments with other members of the team and meetings at the work place or at the social service center were regularly arranged. |
| **Outcomes** | **Outcomes**:  Return to work  Pain (LBP rating scale)  Roland Moris Disability  Fear-avoidance (Orebro)  SF35 - 8 subscales, no composite scores.  **Timepoints**: baseline 12 months  Long |
| **Notes** |  |

Risk of bias table

| **Bias** | **Authors' judgement** | **Support for judgement** |
| --- | --- | --- |
| Random sequence generation (selection bias) | Low risk | "*The first clinical examination was carried out double blind, as randomization subsequently was carried out by a secretary. She phoned a computer generating an automatic voice response on the basis of block-randomization designed by a data management unit at another hospital* " |
| Allocation concealment (selection bias) | Low risk | *See above* |
| Blinding of participants and personnel (performance bias) | High risk | At the following consultations, both participants and caregivers were aware of the result of the randomization. |
| Blinding of outcome assessment (detection bias) | High risk | "*We consider the clinical intervention to be similar in the two intervention groups, as the clinical intervention was managed by the same physician and physiotherapist and they were blinded for group allocation at the first consultation. At later consultations they were not blinded, which may have induced bias in favor of the multidisciplinary group."*  *"Naturally, the consultations with the case manager in the multidisciplinary intervention group were not blinded*" |
| Incomplete outcome data (attrition bias) | Low risk | Study reports there was no missing data |
| Selective reporting (reporting bias) | Low risk | No study protocol cited, however all measures from the methods section are reported in the results. |

Jousset 2004

| **Methods** | **Title:** Effects of Functional Restoration Versus 3 Hours per Week Physical Therapy: A Randomized  Controlled Study  **Location**: France |
| --- | --- |
| **Participants** | **Eligibility/recruitment**  18 to 50 years of age; living within three counties in the west of France; presently engaged in a non limited work contract; threatened in their job situation by chronic LBP; and not relieved by conventional medical or surgical interventions.  **intervention**  n=41 (m:f 30/13 mean age: 41.4 s.d. 7)  **control**  n=41 (m:f 26/15 mean age: 39.4 sd5.9) |
| **Interventions** | **group/individua**l: group  **duration**: high  **In/out**: out (rehabilitation centres)  **intervention**:  The same program was performed in two different rehabilitation centers, 6 hours a day, 5 days a week, during 5 weeks. It was adjusted to each participant’s capacity but always conducted as a group, sports like activity. Frequent contacts between both teams ensured program homogeneity. exercises, occupational therapy, psychiatrist and psychologist.  **active control**:  The program was supervised by private practice physiotherapists. A group of therapists was constituted for this study, and a precise intervention protocol was defined based on the recommendations of the French health accreditation and evaluation agency and on professional experience. Within this group of professionals, the patient was free to choose his therapist. Patients received 1-hour treatment sessions, three times a week during 5 weeks. These included the teaching of a program of exercises that the patient was to perform alone at home for 50 minutes on the two remaining weekdays. |
| **Outcomes** | **Outcomes:**  HADS  medication use  Quebec pain inventory  French version of the Dallas Pain questionnaire (daily and work-leisure activities, anxiety depression, social interest)  pain over last 48 hours VAS  *"Trunk flexibility was measured by fingertip–floor distance. Trunk strength was estimated by the duration of isometric contraction of the flexors and extensors as described by Ito et al and Biering-Sorensen Lifting capacity was evaluated by the Progressive Isoinertial Lifting Evaluation (PILE) test and is presented as a percentage of the body weight. Endurance was measured on bicycle ergometer*s."  **time points**: (medium)  baseline and 6 month follow-up |
| **Notes** | majority male sample.  "*Twenty-five patients referred to the clinic have not been included: 10 lacked sufficient motivation, 4 presented major psychiatric disease, 3 did not present disabling LBP, 3 met medical exclusion criteria, 2 refused to participate because of the delay from the waiting list, 1 had limited duration work contract, 1 was older than 50 years, and 1 refused randomization. The selection period ran for 1 year*" |

Risk of bias table

| **Bias** | **Authors' judgement** | **Support for judgement** |
| --- | --- | --- |
| Random sequence generation (selection bias) | Low risk | Eighty-six patients have been included and were randomized to the FRP group (44 patients) or to the AIT group (42 patients). Block randomization was performed using an eight element permutation table. |
| Allocation concealment (selection bias) | Unclear risk | information not included |
| Blinding of participants and personnel (performance bias) | High risk | not possible due to difference in conditions |
| Blinding of outcome assessment (detection bias) | High risk | The evaluation was not blind as self report measures used. |
| Incomplete outcome data (attrition bias) | Low risk | no drop out |
| Selective reporting (reporting bias) | Low risk | no protocol but all measures reported fully in results |

Kwok 2016

| **Methods** | **Title**: The Effect of a Self-management Program on the Quality-of-Life of Community-dwelling Older Adults with Chronic Musculoskeletal Knee Pain: A Pilot Randomized Controlled Trial  **Location**: Hong Kong |
| --- | --- |
| **Participants** | **Eligibility/recruitment**  All of the participants were older adults (aged 60 or above) who had been recruited at a mobile health centre in Hong Kong. All were able to communicate in Cantonese and therefore had no difficulty participating in activities during the program.  **experimental** (n = 19)  **wait-list** **control** **groups** (n = 27). |
| **Interventions** | **In/out:** out  **duration**: Low. "six 2-hour weekly sessions"  **Group/individual**: mixed  **Experimental**:  self-management program for knee pain, which adopted the construction of evidenced-based Arthritis Self Management programme. The program was delivered to the participants in groups of 6 to 7. The content covers various topics, including information on diseases, an overview of self-management principles (including the five core self-management skills), cognitive symptom management skills (e.g., distraction and relaxation, and managing depressive moods), skills for communicating with family members and health professionals, training in ADLs, training in problem-solving skills and social skills, different forms of counselling and therapy, social support, exercise (including stretching, strengthening, and aerobic exercise), healthy eating.  **Waiting list control**  *"For the control group, the baseline assessment session was followed by the 6-week control period. After that, a post-control-period assessment was conducted. The researcher delivered the identical program to the participants in the control group within one week after the post-control-period assessment."* |
| **Outcomes** | **Outcomes**  Pain = VAS  30 minute chair stand test  60 minute walk test  demographic information  SF-36  pain self-efficacy questionnaire  **Time points:** (short)  Pre, post, 1 month follow-up |
| **Notes** | In the paper they report most of SF36, however data on the subscale 'bodily pain' is only partially available. Further data requested from authors however not provided. BP was not able to be in included in the analysis. |

Risk of bias table

| **Bias** | **Authors' judgement** | **Support for judgement** |
| --- | --- | --- |
| Random sequence generation (selection bias) | Low risk | "*Forty-six participants with chronic knee pain living in the community were randomly assigned"* |
| Allocation concealment (selection bias) | Low risk | "*All were blinded to the group allocation."* |
| Blinding of participants and personnel (performance bias) | High risk | not possible due to differences between groups. |
| Blinding of outcome assessment (detection bias) | High risk | information not included, however, as a self report measure is used to assess HRQoL, and no clear blinding of participants from group allocation has been possible, there is a high risk of bias in this domain. |
| Incomplete outcome data (attrition bias) | Low risk | "There were no drop-outs in this study, so all of the available data were analyzed." |
| Selective reporting (reporting bias) | High risk | no protocol, some data missing from SF-36 sub scales, not clear whether this was gathered and not reported. requested from authors - no reply. |

Lang 2003

| **Methods** | **Title**: Multidisciplinary rehabilitation versus usual care for chronic low back pain in the community: effects on quality of life  comparison of IMPT vs Usual care  **Location**; Germany |
| --- | --- |
| **Participants** | **Eligibility/recruitment**  *"Physicians were instructed to recruit all consecutive patients between May 1997 and May 1998 who met the follow- ing inclusion criteria: seeking treatment of pain in the lumbar and/or thoracic spine with facultative irradiation cranially, caudally or ventrally, persistence of pain for at least 3 months without decreasing duration and no need for surgical inter- vention. Patients were excluded from the study if their in- formed consent for participation in the study could not be obtained by the physician, the patient was not able to answer the questionnaires independently, pain was localized over almost the whole body or there was a history of cancer.*  *The same inclusion and exclusion criteria were used for patients who participated in our MRP between June 1998 and June 1999. These patients were referred from independent physicians within the selected region to the study coordinator in the outpatient facilities of the Departments of Neurology and Orthopedics for inclusion in the MRP."*  **MBI:**  n= 51 (mean age (sd) = 53(10) 57% female)  **usual care:**  n= 157 (mean age (sd) = 53(12) 60% female) |
| **Interventions** | **Out/in: Out**  **Duration: High (160 hours)**  **Individual/group: Group**  **Experimental**: These patients participated in a comprehensive multidisciplinary rehabilitation program with functional restoration of 20 days (3 days per week) and 4 hours per day. Health-care providers in the local community organized the program. The MRP team included four sport teachers who ran a sport center for health sport and sport therapy and were qualified in prevention and rehabilitation of diseases; one clinical psychologist with experience in psychosocial problems of patients with back pain; three physiotherapists experienced in rehabilitation of musculoskeletal disorders; one physician who was experienced in management of chronic back pain. Groups of 7 to 12 persons per group  **Usual care** by independent physicians group No intervention was performed. Patients with low back pain were treated by usual non multidisciplinary and non surgical treatments of physicians and physiotherapists in the community. |
| **Outcomes** | **Outcomes**:  SF-36  Mean pain duration (Brief pain inventory)  pain related interference (Brief pain inventory)  german depression scale  days off work in last 3 months  general satisfaction upon completion of study  **Timepoints**: (short, med)  pre, post, 6month follow-up |
| **Notes** |  |

Risk of bias table

| **Bias** | **Authors' judgement** | **Support for judgement** |
| --- | --- | --- |
| Random sequence generation (selection bias) | High risk | No detil about randomisation  "*CONCLUSIONS: MRP is promising to improve health-related quality of life for patients with chronic back pain in the community. Before implementation of MRP in the repertoire of community medicine, superiority of MRP over usual care should be* ***confirmed by a randomized controlled trial*"** |
| Allocation concealment (selection bias) | High risk | See section above |
| Blinding of participants and personnel (performance bias) | High risk | not possible due to differences between conditions. |
| Blinding of outcome assessment (detection bias) | High risk | information not included, however as a self report measure is used to assess HRQoL, and no clear blinding of participants from group allocation has been possible, there is a high risk of bias in this domain. |
| Incomplete outcome data (attrition bias) | Unclear risk | information not included |
| Selective reporting (reporting bias) | Low risk | no protocol, all measures from methods reported in results |

Lera 2009

| **Methods** | **TItle**: Multidisciplinary treatment of fibromyalgia: Does cognitive behavior therapy increase the response to treatment?  RCT comparing a rehabilitation programme without CBT, with a rehab programme with CBT.  **Location**: Spain |
| --- | --- |
| **Participants** | **Eligibility/recruitment**  *"Female volunteers were recruited from the Fibromyalgia Unit of the Hospital Sant Joan de Déu in Manresa, Barcelona. The inclusion criteria were as follows: (a) having a diagnosis of FM according to American College of Rheumatology criteria [3], made or ratified by the same rheumatologist in all cases; (b) being female; and (c) not being involved in litigation against the government for disability pensions (the aim here is to prevent the participation of patients who were not clearly seeking help in order to feel better). Men were excluded because of their low attendance rate at the unit and to avoid an excessively heterogeneous sample. Suffering from severe depression, psychosis, or delusional disorder was also an exclusion criterion."*  Total n=83  **Exp: IMPT+CBT**  n= 43 (35 completed). 6 month FU n= 30  **Control: IMPT**  n= 40 (33 completed) 6 month FU n= 26 |
| **Interventions** | **in/out**: out  **duration:** Med  **group/individual**: individual  **Control group:**  **rehab programme (**they call it MT)**:** (*use this as control, the intervention in this group only looks at physical aspects*) All patients received the basic MT program, which included 14 group sessions, 1 h per week over 4 months.  *"All subjects received MT. Medical treatment for FM was applied individually, comprising appointments with the rheumatologist for clinical and pharmacological management of pain, sleep disturbances, muscular problems, and secondary symptoms of depression and anxiety; this was based, in principle, on low-dose amitriptyline or analgesics and was administered naturalistically. Patients also received the basic MT program, which included 14 group sessions, 1 h per week over 4 months. Four sessions were dedicated to physical education, led by a rheumatologist and a rehabilitation practitioner and using slides and videos. The content was as follows: (a) description of typical symptoms, causes of the syndrome, course, and treatment; (b) correction of bad habits when lifting weights, body postures, or balancing movements; and (c) organization of daily activities, gradual increase of duration, planning of breaks, and time for physical exercise. The end of each session comprised a group discussion. The remaining 10 sessions were led by the physiotherapist and were devoted to physical exercise and reducing fatigue and stiffness. Patients performed cardio- vascular activities for the first 20 min of each session (on the static bicycle or fast walking, depending on their physical condition) and muscle stretching and restoration for the following 20 min. The final 10 min was set aside for discussion and psychosocial support."*  **Exp: IMPT+CBT**  Ppts received the intervention above along with 22 hours of CBT.  *"The CBT program, developed in line with the recommendations of Bennett and Nielson [19], was led by a clinical psychologist trained in CBT techniques for the control of chronic pain and consisted of 15 group sessions, 90 min per week, before each MT session (except for the first one). CBT included (a) education about the central nervous system and the peripheral sensations, different levels of pain processing, and medullar system of pain control; (b) training in mind– body techniques to reduce physiological and mental distress; (c) behavioral techniques to improve sleep quality; (d) promotion of satisfying activities to increase mental health, self-esteem, and achievement of desired objectives; (e) planning of daily activities, ordering necessary goals, and delaying others according to the intensity of pain; (f) detection and modification of negative cognitions related with pain in order to reduce interference in behavior and a passive lifestyle; (g) training in coping skills and social assertiveness; and (h) psychosocial support. Participants were set tasks to do during the week, and any difficulties with these were discussed in each session."* |
| **Outcomes** | **Outcomes**:  demographic information  The Medical Outcomes Survey Short Form (SF-36) (PCS, and MCS)  Fibromyalgia Impact Questionnaire (FIQ)  FM tender points  The Symptom Checklist-90 — Revised (SCL-90-R)  **time points: (med, med)**  pre, post (4m), 6 month FU |
| **Notes** | The 'MT' group in this study only have physical teaching, used them as a control comparison rathe/as well as the low vs med duration comparison. Ensure not to double count intervention group.  All female Sample  Seems they have mislabelled subscales in the paper  MBI vs IMPT (with higher duration being the comparison group) |

Risk of bias table

| **Bias** | **Authors' judgement** | **Support for judgement** |
| --- | --- | --- |
| Random sequence generation (selection bias) | Low risk | "*Eighty-three accepted and entered the study. After being informed of the nature of the trial, placed consecutively onto a waiting list. When a subgroup of 20–22 subjects from the waiting list was reached, they were randomly assigned (by the flip of a coin) to either the MT group or the MT+CBT group."* |
| Allocation concealment (selection bias) | Low risk | See description above |
| Blinding of participants and personnel (performance bias) | Unclear risk | "*The two groups met on different days of the week in order to prevent the exchange of information. All efforts were made to maintain participants as blind as possible to the contents of the other treatment.".*  However, individuals in the CBT group also given 1 to 1 sessions with psychologist, and all other aspects were group interventions. Depending on how informed their consent was, its possible this difference could alert people to their allocation. |
| Blinding of outcome assessment (detection bias) | Unclear risk | Some aspects: *"A resident physician specifically trained and blinded to the group assignation explored the 18 bilateral pairs of tender points related to FM".*  Further information not provided about analysis of numerical data. A self report measure was used to assess HRQoL, however as some effort has been made to blind participants the risk posed here is unclear. |
| Incomplete outcome data (attrition bias) | Low risk | Intention to treat analysis |
| Selective reporting (reporting bias) | Low risk | no protocol cited, however all measures from methods reported in results section. |

Luciano 2013

| **Methods** | **Title**: Cost-Utility of a Psychoeducational Intervention in Fibromyalgia Patients Compared With Usual Care An Economic Evaluation Alongside a 12-Month Randomized Controlled Trial  **Location:** Spain |
| --- | --- |
| **Participants** | **Eligibility/recruitment:**  Three general practices within the metropolitan area of Barcelona (Spain) participated in the study. The general practitioners (GPs) at these centers referred those patients suspected of having FM to the hospital. A rheumatologist from that hospital confirmed or ruled out the diagnosis of FM using the ACR criteria, and included the patients in a database if she/he received a FM diagnosis. In the present study, the sample pool consisted of all patients included in this database between 2005 and 2008. All patients aged 18 to 75 years and contactable by telephone were candidates for inclusion in the study. Exclusion criteria: diagnosis of FM not based on the ACR criteria, cognitive impairment, presence of physical/psychiatric limitations (any severe medical illness, psychotic symptoms or disorders, or substance abuse) that impeded participation in the study assessments, life expectancy <12 months, and absence of schooling.  total n=216  **exp**  n= 108 (mean age (sd) = 55.17(8.58) 97.2%female)  **control**  n=108 (mean age (sd) = 55.42 (8.63) 98.1 %female) |
| **Interventions** | **Out/in**: out  **duration:** low  **Individual/group**: group  **EXP group**  "*consisted of nine 2- hour sessions delivered over a 2-month period (1 session/wk). Participants were allocated to groups with a maximum of 18 participants per group. The educative part of the program (5 sessions) included information about typical symptoms, usual course, comorbid medical conditions, potential causes of the illness, the influence of psychosocial factors on pain, current pharmacological and non pharmacological treatments, the benefits of regular exercise, and the typical barriers to behavior change. The speakers included 4 GPs and 1 rheumatologist. Participants were encouraged to be active, to ask questions, and to discuss issues with the speakers or with other participants. The autogenic training (4 sessions) was led by a clinical psychologist with the main aim of increasing participants’ pain contro*l."  **Control group:**  "*Patients in the control group received standard medical attention. In Spain, the treatment provided is mainly pharmacological and adjusted to the symptomatic profile of the fibromyalgic patient. Counseling about aerobic exercise adjusted to patients’ physical limitations is usually provided. Usual care is an ideal comparator in the case of FM, for which no specific treatment is considered the gold standard of care*." |
| **Outcomes** | **Outcomes**  Demographics  Fibromalgia Impact questionnaire  Euroqol 5D  Clinet service receipt inventory  **Timepoints**:  pre,post, 6 month, 12 months |
| **Notes** | Trial Registration: NCT00550966.  Almost entirely female sample |

Risk of bias table

| **Bias** | **Authors' judgement** | **Support for judgement** |
| --- | --- | --- |
| Random sequence generation (selection bias) | Unclear risk | The study cites its trial registration and some other studies which used the same data, however there are no details provided about how participants are randomised. They were selected retrospectively from a database, its not explained how ppts came to either be in the usual care group or the intervention |
| Allocation concealment (selection bias) | Unclear risk | Information not included |
| Blinding of participants and personnel (performance bias) | High risk | not possible due to group differences |
| Blinding of outcome assessment (detection bias) | High risk | Blinding of assessor noted in the study protocol, however as a self report measure is used to assess HRQoL, and no clear blinding of participants from group allocation has been possible, there is a high risk of bias in this domain. |
| Incomplete outcome data (attrition bias) | Low risk | intention to treat applied |
| Selective reporting (reporting bias) | Low risk | All measures noted in methods included the intervention |

Martins 2014

| **Methods** | **Title**: Randomized controlled trial of a therapeutic intervention group in patients with fibromyalgia syndrome  **Location**: Brazil |
| --- | --- |
| **Participants** | **Eligibility/recruitment:** patients diagnosed with FMS (test group – TG) were recruited according to criteria of the Ameri- can College of Rheumatology,1 of both genders, with enough cognitive level to understand the procedures and follow the directions given. Patients with psychiatric disease and no clinical follow-up in the Pain Clinic, Hospital de Base, were excluded. The control group (CG) consisted of patients who were in interconsultation in the Pain Clinic and without diagnosis of musculoskeletal and neurological disorders, or with disabling complaints in these systems, with a recommendation for walking (pelvic pain, migraine, inflammatory bowel pain post-herpetic neuralgia). CG consisted of subjects matched for age and educational level in relation to TG (n = 15).  **total** n=27 (mean age (sd) = 42.5 (9.8) 58.5 % female)  **exp**:  n= 12 (mean age (sd) =39,5±7,8 62% female)  **Control**  n=15 (mean age (sd) = 45,5±8,3 55% female) |
| **Interventions** | **out/in:** out  **Duration:** low  **group/individual:** group  **exp**:  "*sessions lasted 60 minutes each and happened once a week for 12 weeks for each of the study groups, being conducted by a physician, occupational therapist, physiotherapist, psychologist and social worker.*" sessions included exercise, relaxation, education etc.  **Control**:  "*The control group (CG) consisted of patients who were in interconsultation in the Pain Clinic and without diagnosis of musculoskeletal and neurological disorders, or with disabling complaints in these systems, with a recommendation for walking (pelvic pain, migraine, inflammatory bowel pain post-herpetic neuralgia).*"  No information about what they did, sounds like usual care. |
| **Outcomes** | **Outcomes**  Post sleep protocol  SF-12  Visual analogue scale for pain  Fibromalgia Impact questionnaire  HADS  **Timepoints**: (short)  pre, post |
| **Notes** | Intervention and control group information vague |

Risk of bias table

| **Bias** | **Authors' judgement** | **Support for judgement** |
| --- | --- | --- |
| Random sequence generation (selection bias) | Unclear risk | Information not included |
| Allocation concealment (selection bias) | Unclear risk | information not included |
| Blinding of participants and personnel (performance bias) | High risk | not possible due to differences in conditions |
| Blinding of outcome assessment (detection bias) | High risk | information not included. However, as a self report measure is used to assess HRQoL, and no clear blinding of participants from group allocation has been possible, there is an unclear risk of bias in this domain. |
| Incomplete outcome data (attrition bias) | Unclear risk | information not included |
| Selective reporting (reporting bias) | Low risk | no protocol cited, all measures from methods reported in results |

Monticone 2012

| **Methods** | **Title**: Chronic neck pain and treatment of cognitive and behavioural factors: results of a randomised controlled clinical trial  **Location:** Italy |
| --- | --- |
| **Participants** | **Eligibility/recruitment**  The inclusion criteria were a diagnosis of chronic non-specific NP (i.e. a documented history of pain lasting more than 3 months), a good understanding of the Italian language and an age of more than 18 years. The exclusion criteria were cognitive impairment (deficits in higher reasoning, forgetfulness, learning disabilities, concentration difficulties, decreased intelligence and other reductions in mental functions) and all causes of specific NP, such as whiplash injuries, previous cervical surgery, infection, fracture or malignancy, and systemic or neuromuscular diseases. Any subjects who had previously participated in a cognitive-behavioural intervention for neck or low back pain (LBP) were also excluded.  Outpatients referred to the physical medicine and rehabilitation unit of our hospital were consecutively included in the study between December 2007 and December 2008.  **exp**:  n= 40 baseline 40 completers. Mean age (sd): 54.97 (13.83). Sex m/f: 10 (25)/30 (75)  **control**:  n= 40 baseline 35 completers. Mean age (sd): 44.20 (11.44). Sex m/f: 10 (25)/30 (75) |
| **Interventions** | **Out/in**: Out  **Duration:** Low  **Group/individual**: individual  **Exp:** Physiotherapy plus cognitive-behavioural therapy (PTcb group).  "*The intervention lasted from a minimum of 2 months to a maximum of 3 months. All of the subjects followed the rehabilitation programmes individually; two physiotherapists were separately responsible for the interventions in each group. The physiotherapists for both groups were allowed to arrange up to 12 sessions lasting 45–50 min each, one or twice a week; In addition to following the same physiotherapy programme as above, in all of the patients of this group, the physiotherapists concentrated on the subjects’ beliefs, negative automatic thoughts and behaviours. Using a process of correct re-learning and cognitive reconditioning, the approach consisted of gradually recovering physical abilities and treating some psychosocial characteristics of patients with chronic pain, such as fear of movement, hypervigilance, catastrophising and the reduction of social relationships. In addition to following the same physiotherapy programme as above, in all of the patients of this group, the physiotherapists concentrated on the subjects’ beliefs, negative automatic thoughts and behaviours. Using a process of correct re-learning and cognitive reconditioning, the approach consisted of gradually recovering physical abilities and treating some psychosocial characteristics of patients with chronic pain, such as fear of movement, hypervigi- lance, catastrophising and the reduction of social relationships*"  **Control**: Physiotherapy alone (PT group),  *"consisting of a multi- modal approach, including passive and active mobilisation of the neck, and exercises aimed at improving postural control, strengthening muscles and stretching. Passive mobilisation involved manual therapy for accessory and physiological movements designed to improve the range of motion. Postural control was developed by means of exercises aimed at developing motor control of the deep muscles of the neck and scapula. All of the procedures were addressed to improve upper quadrant mechanics and thoracic posture. The strengthening exercises were introduced only after motor control had been regained. Segmental stretching involved the upper trapezius, levator scapulae and scalenus muscles. The patients were also encouraged to perform the same exercises at home. Ergonomic advice was given to facilitate the modification of daily living activities."* |
| **Outcomes** | **Oucomes**:  Treatment satisfaction  neck pain and disability scale  pain - single item rating scale  SF-36  **Timepoints**: (short, long)  Pre, post, 1 year follow-up |
| **Notes** | Additional Information about the duration was requested and provided by the author.  Individual interventions. |

Risk of bias table

| **Bias** | **Authors' judgement** | **Support for judgement** |
| --- | --- | --- |
| Random sequence generation (selection bias) | Low risk | subjects were randomly programmes. Randomisation was performed centrally using a computerised procedure (SAS PROC PLAN) |
| Allocation concealment (selection bias) | Unclear risk | not explicitly stated |
| Blinding of participants and personnel (performance bias) | High risk | The patients were partially blinded as they were unaware of the hypothesised differences between the groups, but they were aware of what treatment they were participating in. Personel blinding not mentioned. |
| Blinding of outcome assessment (detection bias) | Unclear risk | The investigators who obtained and assessed the outcome data were blinded to the patients’ treatment. However as a self report measure is used to assess HRQoL, and no consistent blinding of participants from group allocation has been clearly described, there is an unclear risk of bias in this domain. |
| Incomplete outcome data (attrition bias) | Low risk | intention to treat applied |
| Selective reporting (reporting bias) | Low risk | all measures mentioned in materials reported in results. No protocol cited. |

Monticone 2013

| **Methods** | **Title**: Effect of a Long-lasting Multidisciplinary Program on Disability and Fear-Avoidance Behaviors in Patients With Chronic Low Back Pain Results of a Randomized Controlled Trial  **Location**: Italy |
| --- | --- |
| **Participants** | **Eligibility/recruitment**  The inclusion criteria were a diagnosis of nonspecific CLBP (ie, a documented history of pain lasting for >3 mo), a good understanding of Italian, and an age of > 18 years. The exclusion criteria were cognitive impairment and all causes of specific CLBP, such as previous spinal surgery, deformity, infection, fracture or malignancy, and systemic or neuromuscular diseases. Any patients receiving compensation for work-related disabilities or who had previously participated in a cognitive-behavioral intervention for CLBP were also excluded.  Exp:  n= 45 mean (sd) = 48.9 (7.97) m/f = 18/27  control  n=45 mean (sd) = 49.71±7.01 m/f = 20/25 |
| **Interventions** | **In/out;** Out  **Intensities**: Low CBT and exercise: 5/60 mins then 12 more hours x2 about 36-40 hours  **Group/individual**; mixed  **Delivered by:**  These involved 2 physiatrists, a psychologist, and 4physiotherapists. The experimental group underwent a multidisciplinary program consisting of CBT and exercise training; the control group were only given exercise.  Both programs lasted 5 weeks (instructive phase) plus 1 year (reinforcement phase)  **CBT (Experimental Group)**  **"*T****he experimental group underwent a multidisciplinary program consisting of CBT and exercise training; the control group were only given exercise"*  *"Under the supervision of a clinical psychologist, the purpose was to modify fear of movement beliefs, catastrophizing thinking, and negative feelings, and ensuring gradual reactions to illness behaviors. The main situations avoided by the patients were pointed out on the basis of the fear-avoidance beliefs emerging from their usual activities and the results arising from the presentation of images of back-stressing activities. After explaining the fear-avoid- ance model,8,9 the psychologist educated the patients to view their pain as something that can be self-managed rather than a serious disease that needs careful or vigilant protection. Correct relearning and cognitive reconditioning were based on developing awareness of the problem and seeking a means of reacting to frightening thoughts. The patients were assisted in transferring attention from their fear of movement to increasing the level of activity by means of graded exposure to the situations they had previously identified as dangerous. Negative appraisals were also taken into account and discussed to promote the re- acquisition and development of neglected coping strategies by means of communication, motivation, and sharing the goals to be reached during the usual activities of everyday life.*  *All of the patients followed the 60-minute CBT sessions individually once a week for 5 weeks (instructive phase), after which the psychologist met the patients for further 1-hour sessions once a month for a year to verify their growing ability to manage chronic pain and reinforce the self-management of dysfunctional thoughts and wrong behaviors related to the fear of movement (reinforcement phase)."*  **Exercise Training (Experimental and Control Group)**  *"The patients underwent a multimodal motor program consisting of active and passive mobilizations of the spine, and exercises aimed at stretching and strengthening muscles, and improving postural control. The passive mobilization involved manual therapy for accessory and physiological movements to improve the range of motion. The stretching was segmentary and involved the groups of lower limb and back muscles. Basic exercises were gradually introduced to improve spinal deep muscle awareness, and the patients learned a specific strengthening technique for the same muscles. Postural control was developed by means of exercises aimed at developing motor control of the spine and pelvis.17,18 Ergonomic advice was provided by means of a booklet given to the patients during the first session to facilitate the modification of daily living activities.*  *All of the patients followed the exercise program individually. Under the supervision of a physiatrist, 2 physiotherapists were separately responsible for the interventions in each group, and were both allowed to arrange 10, 60-minute sessions twice a week for 5 weeks (instructive phase). The physiotherapists completed an ongoing treatment diary for each session. The patients were asked to continue the exercises taught actively in twice-weekly 60- minute sessions for 1 year (reinforcement phase), during which they received telephone reminders from personal staff aimed at strengthening their adhesion to these indications.*" |
| **Outcomes** | **Outcomes**:  Roland Morris  Tampa Scale for Kinesiophobia (TSK)  Short-Form (36) Health Survey (SF-36), (Italian)  **timepoints** : short, long  "questionnaires were completed before treatment (T1), 5 weeks later (at the end of the instructive phase, T2), and then 12 months (post-treatment analysis, T3) and 24 months after the end of the instructive phase (1-year follow-up, T4)." |
| **Notes** |  |

Risk of bias table

| **Bias** | **Authors' judgement** | **Support for judgement** |
| --- | --- | --- |
| Random sequence generation (selection bias) | Low risk | "*Immediately after the patients had given their consent, the physiatrists e-mailed the Principal Investigator, who randomized the patients to one of the 2 treatment programs using a list previously generated by a biostatistician (SASPROC PLAN)16 and delivered to the Principal Investigator with blinded treatment codes.*" |
| Allocation concealment (selection bias) | Low risk | see above |
| Blinding of participants and personnel (performance bias) | High risk | "For obvious reasons, the physiatrists, psychologist, and physiotherapists could not be blinded." |
| Blinding of outcome assessment (detection bias) | Unclear risk | "The Principal Investigator obtaining and assessing the outcome data, and the biostatisticians making the analyses, were all blinded to the treatments."  However, as a self report measure is used to assess HRQoL, and no clear blinding of participants from group allocation has been described, there is an unclear risk of bias in this domain. |
| Incomplete outcome data (attrition bias) | Low risk | No drop out |
| Selective reporting (reporting bias) | Low risk | All measures reported in methods included in results. |

Monticone 2014

| **Methods** | **title:** A multidisciplinary rehabilitation programme improves disability, kinesiophobia and walking ability in subjects with chronic low back pain: results of a randomised controlled pilot study  **Location**: Italy |
| --- | --- |
| **Participants** | **Eligibility/recruitment:**  The study involved outpatients aged [18 years with non- specific CLBP (i.e. a documented history of pain lasting [3 months) and a good understanding of Italian who were referred to our hospital between January and June 2013. Patients with central or peripheral neurological signs, cognitive impairment (i.e. deficits in higher reasoning, forgetfulness, learning disabilities, concentration difficul- ties, decreased intelligence and other reductions in mental functions), severe cardio-vascular and respiratory comor- bidity, prior spine surgery, ambulation deficits due to neurological or orthopaedic impairments were excluded, as were those who were pregnant or who had previously participated in cognitive–behavioural interventions.  All of the patients satisfying the entry criteria were asked to give their written informed consent, to declare their willingness to comply with whichever treatment option they were randomly assigned to, and to attend all of the follow-up visits.  **Exp**:  n= 10 Mean age (SD): 58.9 (16.4) male/female 3/7  **control**:  n= 10 Mean age (SD) 56.6 (14.4) male/female 6/4 |
| **Interventions** | **Out/in**: Out  **Intensities**: low  **Individual/group**: mixed  **exp:**  The subjects in the experimental group attended individual 60-min cognitive–behavioural sessions once a week for 8 weeks, and the subjects of both groups attended individual 60-min motor training sessions twice a week for 8 weeks. The intervention involved two physiatrists, a psychologist, an occupational therapist, and two physiotherapists. The experimental group followed a multidisciplinary programme consisting of motor training integrated with cognitive– behavioural therapy;  **control group**  only did physical exercises. |
| **Outcomes** | **Outcomes**:  Oswestry Disability Index,  Tampa Scale for Kinesiophobia,  the Pain Catastrophizing Scale  pain numerical rating scale Short-Form Health Survey  Spatio-temporal gait parameters  **Timepoints**: (short)  pre, post, 3 months |
| **Notes** | very small sample size due to pilot study |

Risk of bias table

| **Bias** | **Authors' judgement** | **Support for judgement** |
| --- | --- | --- |
| Random sequence generation (selection bias) | Low risk | "principal investigator (PI) randomised them to one of the treatment programmes using a list of blinded treatment codes previously generated by a biostatistician" |
| Allocation concealment (selection bias) | Low risk | "Immediately after the patients had given their consent, the principal investigator (PI) randomised them to one of the treatment programmes using a list of blinded treatment codes previously generated by a biostatistician and an automatic assignment system in order to conceal the allocation." |
| Blinding of participants and personnel (performance bias) | High risk | "The physiatrists, the psychologist, the physiotherapists, and the patients could not be blinded." |
| Blinding of outcome assessment (detection bias) | High risk | The PI obtaining and assessing the outcome data, and the biostatisticians making the analyses were blinded to the treatments.  However, as a self report measure is used to assess HRQoL, and no clear blinding of participants from group allocation has been described, there is an clear risk of bias in this domain. |
| Incomplete outcome data (attrition bias) | Low risk | no drop outs |
| Selective reporting (reporting bias) | Low risk | No protocol, but all measures from methods reported in results. |

Morone 2011

| **Methods** | **Title**: Quality of life improved by multidisciplinary back school program in patients with chronic non-specific low back pain: a single blind randomized controlled trial  **Location**: Italy |
| --- | --- |
| **Participants** | **Eligibility/recruitment**  *"performed in a rehabilitation center. The nature and purpose of the study were presented to patients and written informed consent was obtained. The study was approved by the Ethical Committee. People enrolled in the study were referred to the Physiatry dept. of Policlinico Umberto I, Sapienza University, from February 2007 to February 2008."*  *"Inclusion criteria were: age (between 18 and 80) and chronic non-specific LBP persisting for at least 3 months. Exclusion criteria were: acute pain; low back pain due to specific causes (fracture, spondylolisthesis, disc herniation and lumbar stenosis); presence of rheumatological, neurological or oncological concomitant disease; back surgery before study; cognitive impairment (MMSE 17 score s24) and pregnancy."*  **MBI**:  n=41 (mean age (sd)= 61.2 (13.3) male/female71/24)  **Control**:  n= 29 (mean age (sd)= 58.6 (12.2) male/female 8/21) |
| **Interventions** | **Out/in**: Out  **Intensities**: Low  **group/individual**: Group  **exp**:  "Intensive 4 week intervention made by multidisciplinary professional healthcare. It was conducted in a rehabilitation centre made by 10 session interventions. All sessions lasted an hour. Each group included 4 or 5 participants."  **Control**:  treatment as usual. |
| **Outcomes** | **Outcomes**  demographics  SF-36  VAS  Waddell Disability index  Owestry Disability index  **Timepoints**: (short, med)  pre, post, 3 months, 6 months |
| **Notes** |  |

Risk of bias table

| **Bias** | **Authors' judgement** | **Support for judgement** |
| --- | --- | --- |
| Random sequence generation (selection bias) | Low risk | *"Randomization was performed by means of extraction each time on a group of 15 patients: 5 patients were allocated in a treatment group performing back school programme, other 4 patients in a similar treated group and the last 6 in the control group"* |
| Allocation concealment (selection bias) | Unclear risk | detail unclear |
| Blinding of participants and personnel (performance bias) | High risk | Blinding of participants and researchers not possible due to group differences |
| Blinding of outcome assessment (detection bias) | High risk | *"Another physician was involved in patients' assessment and was unaware of treatment"*  However, as a self report measure is used to assess HRQoL, and no consistent blinding of participants from group allocation has been possible, there is a high risk of bias in this domain. |
| Incomplete outcome data (attrition bias) | High risk | No missing data strategy discussed |
| Selective reporting (reporting bias) | Low risk | no protocol cited, all measures reported however lacking some detail in parts |

Nost 2018

| **Methods** | **Title**: Short-term effect of a chronic pain self- management intervention delivered by an easily accessible primary healthcare service: a randomised controlled trial  An open, pragmatic, parallel group randomised controlled trial (RCT) was conducted from August 2015 to March 2017  **Location**: Norway |
| --- | --- |
| **Participants** | **Eligibility/recruitment**  "*Recruitment for the RCT began in September 2015 and ended in October 2016. Individuals who met the following inclusion criteria were admitted: adults of 18 years of age or older, self-reported pain for 3months or more, able to take part in group discussions in Norwegian and a signed agreement to accept randomisation to one of the trial activities after a full explanation of the trial. The exclusion criteria were as follows: inability to participate in low-impact physical activity for at least 1 hour, pain arising from malignant diseases and inability to consent to study participation.*  *The opportunity for people with chronic pain to participate in the trial was communicated through posters and information leaflets distributed to general practitioners, physiotherapists, relevant departments at the hospital, Norwegian Labour and Welfare Administration offices and other relevant organisations in the municipality. To encourage self-referrals for the trial, advertisements were also placed in local newspapers, websites, social media and email invitations to patient organisations. Those interested in participating were encouraged to contact the first author by either phone or email"*  *"Of the 208 people who responded to the trial announcement, 87 declined to participate after receiving additional information or did not meet the inclusion criteria, leaving 121 participants suitable for inclusion"*  **exp**  n=60 (88.3% female) Mean age 52.1 years  **Control**  n=61 (86.9% female) Mean age 53.3 years |
| **Interventions** | **Duration**: low  **Out/in**: out  **Group/individual**: group  **Exp**:  *"The course used elements from cognitive–behavioural therapy (CBT) by creating a focus on thoughts, emotions and actions related to pain. When discussing the participants’ experiences with pain in everyday life, the instructors focused on activating events, beliefs or presumptions related to the events as well as consequences in terms of feelings, physical symptoms and behaviours. The course included topics such as pain theory, barriers in everyday life due to chronic pain, problem solving, goal setting and techniques to deal with fatigue, poor sleep, frustration and isolation. The course aimed to teach skills such as setting specific, functional and realistic goals, activity pacing and structured problem solving. The movement exercises based on psychomotor physiotherapy63 concluding each session, aimed to improve balance, posture and breathing, providing the participants with techniques to increase body awareness and the ability to relax...course was delivered as 2.5-hour weekly group sessions during the day (12:30–15:00) for a period of 6weeks and a total of 15hours. The self-management course was facilitated by two HLC physiotherapists experienced in working with behaviour changes, coping and chronic pain. One of the physiotherapists was educated in psychomotor physiotherapy and had extensive experience from a multidisciplinary hospital pain clinic."*  **control**:  "*The control group was offered a group-based physical activity that was already available as an activity at the HLC. The low-im- pact physical activity was a weekly 1hour drop-in session during the day (13:00–14:00) for a period of 6weeks, which consisted of walking and simple strength exercises (eg, squats and push-ups against a tree or a bench)...Participation was voluntary, which is in line with the drop-in policy for this type of activity at the HLC. Two dedicated instructors familiar with physical exercise led the activity. The instructors encouraged the exchange of information among the participants rather than answering questions and giving advice themselves. Hence, there was no education for the control group* " |
| **Outcomes** | **Outcomes**  Patient activation was assessed using the Patient Activation Measure (PAM)  The short version of the Brief Pain Inventory (BPI)  participants reported experienced pain during the previous week using a one-item, 100 mm visual analogue scale (VAS)  The Hospital Anxiety and Depression Scale  Self-efficacy was measured using the Pain Self-Efficacy Questionnaire (PSEQ)  The 13-item Norwegian version of the Sense Of Coherence (SOC) scale was used to assess the capacity to respond to stressful situations and remain healthy  The EuroQoL (EQ-5D-5L) was used to assess health-related quality of life  The Arizona Integrative Outcomes Scale (AIOS) was used to measure an overall experience of well-being using a one-item,  To assess global self-rated health, participants were asked: ‘By and large, would you say that your health is: poor, not so good, good, very good or excellent’?  In addition, a measure of physical ability was included using the 30s chair to stand test to measure lower body strength  **Timepoints**; short  baseline - 3 months |
| **Notes** |  |

Risk of bias table

| **Bias** | **Authors' judgement** | **Support for judgement** |
| --- | --- | --- |
| Random sequence generation (selection bias) | Low risk | "Following an individual randomisation procedure from a computer-based internet trial service provided by a third party (Unit for Applied Clinical Research at the Norwegian University of Science and Technology, NTNU), participants were consecutively randomly allocated to one of two trial arms with a ratio of 1:1 after completing the baseline assessment" |
| Allocation concealment (selection bias) | Low risk | "Following an individual randomisation procedure from a computer-based internet trial service provided by a third party (Unit for Applied Clinical Research at the Norwegian University of Science and Technology, NTNU), participants were consecutively randomly allocated to one of two trial arms with a ratio of 1:1 after completing the baseline assessment" |
| Blinding of participants and personnel (performance bias) | High risk | The blinding of participants and instructors was not possible due to the nature of the interventions; however, the research assistant who supervised the physical ability test at the follow-up appointment was blinded to allocation. |
| Blinding of outcome assessment (detection bias) | High risk | "The blinding of participants and instructors was not possible due to the nature of the interventions; however, the research assistant who supervised the physical ability test at the follow-up appointment was blinded to allocation." |
| Incomplete outcome data (attrition bias) | Low risk | Patterns of missing values were investigated and determined to be missing at random.  The effect of the intervention was assessed using an intention-to-treat and per-protocol procedures. |
| Selective reporting (reporting bias) | Low risk | "The protocol for the trial has been published previously.25 There were no changes to the methods after trial commencement." |

Nygaard 2020

| **Methods** | **Title**: Group-based multimodal physical therapy in women with chronic pelvic pain: A randomized controlled trial  **Location**: Norway |
| --- | --- |
| **Participants** | **Eligibility**  *Inclusion criteria* Norwegian-speaking women Age 20-65 years Chronic pelvic pain diagnosis Motivated to participate in a group intervention *Exclusion criteria* Malignancy and conditions requiring special medical attention Pregnancy at the time of inclusion or childbirth during the previous 12 months Drug addiction Serious psychiatric diagnosis Previous treatment by the physical therapists involved in the intervention Intra-abdominal or pelvic surgery within the last 6 months Botulinum toxin injections in the pelvic area in the last 4 months  Sixty-two women were randomly assigned between March 2015 and November 2016. Data collection was completed in January 2017, with the data of 26 and 25 women available for the 12-month analyses from the intervention and comparator groups, respectively  **exp**  (n = 30) (mean age 39.7)  **control**  (n = 32) (mean age 36.2) |
| **Interventions** | **Duration**: High  **Out/in:** Out  **Group/individual**: Group  **Exp:**  The study intervention was based on the biopsychosocial model,11 combined body awareness therapy,8,12 patient education,9,13 and cognitive approach of “acceptance and commitment therapy”10 in a group setting. There was a pre-planned schedule, with an initial 10- day session followed by 2-day sessions after 3, 6, and 12 months. The aim was to reduce pain and improve daily function by challenging avoidance habits and providing new positive body experiences.8,12 Detailed information about the intervention is shown in Supporting material, Table S1 (schedule) and Table S2 (TidiER checklist).  **Control:**  Women in the comparator group were referred to a physical therapist in primary health care with competence in women's health. The therapists received an information letter (Supporting material, Appendix S1), and they were asked to provide treatment according to their academic competence and in consultation with the woman. The deductibles of the physical therapy treatment were refunded.  Duration information. First 2 weeks are 7 hours a day for 5 days (7x5)x2=70 hours  then a further 6 days over the remaining 12 months - 7x6 = 42  total hours - 112 (high duration) |
| **Outcomes** | **Outcomes**  mean pain intensity  Movement patterns were assessed using the Standardized Mensendieck test  Pain-related fear of physical movement and activity was registered with the validated Tampa scale for Kinesiophobia  Health-related quality of life was measured using the EQ5D-5L questionnaire. An EQ5D-index and an EQ visual analogue scale  Symptoms of anxiety and depression were recorded using the Hopkins Symptom checklist-25  **Timepoints**: long  baseline - 12months |
| **Notes** | entirely female sample |

Risk of bias table

| **Bias** | **Authors' judgement** | **Support for judgement** |
| --- | --- | --- |
| Random sequence generation (selection bias) | Low risk | The randomization database was administered by the Clinical Research Department at the hospital, and was available only for the primary researcher and the project leader. |
| Allocation concealment (selection bias) | Low risk | The randomization database was administered by the Clinical Research Department at the hospital, and was available only for the primary researcher and the project leader. |
| Blinding of participants and personnel (performance bias) | High risk | not possible due to differences between study groups. |
| Blinding of outcome assessment (detection bias) | High risk | Baseline data were collected at the outpatient clinic at the time of inclusion before randomization, all outcomes were collected again after 12 months. Information about pain intensities was also collected by mail at 3 and 6 months. Women who did not manage to travel to the hospital for the post-test for practical reasons were contacted by phone and mail. Two physical therapists (ASN and MFE) performed the baseline and follow-up tests.  However, as a self report measure is used to assess HRQoL, and no clear blinding of participants from group allocation has been possible, there is an unclear risk of bias in this domain. |
| Incomplete outcome data (attrition bias) | Low risk | In case of missing data on sub-items of the secondary outcome mea- sures, averages of the available responses were used |
| Selective reporting (reporting bias) | High risk | "Regrettably, the registration of the primary outcome at clinicaltrials. gov was misleading, including mean, least, and worst recorded pain intensities at three different time-points. Some secondary outcomes were also registered as measured at different time-points. However, the objective of the trial was to analyse changes from baseline to 12 months. Additionally, the sample size calculation was 46 and not 50, as registered at clinicaltrials.gov." |

Paolucci 2017

| **Methods** | **Title**: Improved interoceptive awareness in chronic low back pain: a comparison of Back school versus Feldenkrais method  single blind randomised control trial  **Location**: Italy |
| --- | --- |
| **Participants** | **Eligibility/recruitment**  *"The study was performed in the outpatient rehabilitation center of Policlinico Umberto I Hospital, Rome, Italy. Subjects aged between 30 and 75 years with a diagnosis of chronic nonspecific LBP for at least 3 months were eligible for inclusion. The exclusion criteria were: acute LBP; LBP due to specific causes; concomitant rheumatological, neurological, or oncological disease; previous back surgery; severe cognitive impairments; and pregnancy. Seventy-two patients were screened in the study from September 2014 to March 2015, 53 of whom were enrolled and then randomized"*  **Exp:** Back School group  (BG, N=27, 81% females, mean age 60.70 ± 11.72 years, BMI 26.18 ± 2.62, VAS 5.4 ± 4.1)  **Control:** Feldenkrais group  (FG, N = 26, 83% females, mean age 61.21 ± 11.53 years, BMI 25.55 ± 2.62, VAS 5.3 ± 2.7) |
| **Interventions** | **In/out**: Out  **Intensity**: low  **Group/individual**: Mixed  10 intervention sessions both rehabilitation groups. Each session lasted 1 h and was performed twice per week for 5 weeks to ensure better compliance and participation of work-ing patients. Each group comprised 4 or 5 participants  **Intervetnion**:  The Back School program was a mild 5-week intervention that was administered by a multidimensional professional team.general anatomical information on the spine and its function and ergonomic positions in daily living. Teachers (physicians) also gave information on chronic pain and LBP, the related psychological aspects, and stress management. Physiotherapists conducted another 9 sessions that included exercises that were based on diaphragmatic breathing  **Control**:  Feldenkrais method is based on awareness through movement lessons, which are verbally guided explorations of movement, conducted by a physiotherapist. The 10 lessons were structured to enhance trunk mobility in improving overall function without pain |
| **Outcomes** | **Outcomes**:  Pain VAS  the McGill Pain Questionnaire (MPQ)  Waddel Disability Index  pain rating index (PRI) and 4 subscales  pain intensity (PPI), based on a 5-point intensity scale  Multidimensional Assessment of Interoceptive Awareness Questionnaire  SF-36  **Time points**: (short)  baseline, 3 month follow-up |
| **Notes** |  |

Risk of bias table

| **Bias** | **Authors' judgement** | **Support for judgement** |
| --- | --- | --- |
| Random sequence generation (selection bias) | Low risk | "...were enrolled and then randomized to the Feldenkrais group..."  +  " a 1:1 ratio according to a computer-generated randomization list" |
| Allocation concealment (selection bias) | Low risk | Allocation was concealed for patients and examiner: patient’s allocation was obtained from a computer generated list (developed using the website www.random.org), that was printed and each number was covered by a patch by a researcher not involved into patient’s assessment. Patches were successively removed after each patient’s inclusion in the study, revealing only his/her allocation by another researcher not involved into the patient’s assessment. |
| Blinding of participants and personnel (performance bias) | Unclear risk | not clear but unlikely due to difference in interventions. |
| Blinding of outcome assessment (detection bias) | Unclear risk | "the clinical assessor was unaware of group allocation"  However, as a self report measure is used to assess HRQoL, and no clear blinding of participants from group allocation has been described, there is an unclear risk of bias in this domain. |
| Incomplete outcome data (attrition bias) | Low risk | Intention to treat |
| Selective reporting (reporting bias) | Low risk | All scales from the protocol included (clinicaltrials.gov identifier:NCT0223155). however "pain rating index (PRI) and 4 subscales" was not mentioned in protocol but reported in study. |

Roche-Leboucher 2011

| **Methods** | **Title**: Multidisciplinary Intensive Functional Restoration Versus Outpatient Active Physiotherapy in Chronic Low Back Pain  "prospective open clinical trial"  **Location**: France |
| --- | --- |
| **Participants** | **Eligibility/recruitment**  "*Patients were eligible for inclusion if they were referred to a multidisciplinary LBP clinic, in a level 1 hospital, be- tween January 2000 and April 2003. They were evaluated consecutively and independently in this multidisciplinary clinic, by a physical medicine and rehabilitation specialist, an occupational medicine specialist, a psychologist, and an ergonomist. They were assessed on their medical and employment histories and had a standardized medical examination. The purpose of the study was explained. This study was approved by the local ethics committee.*  *The inclusion criteria were nonspecific chronic LBP for at least 3 months, age 18 to 50 years, on sick leave or at risk of work disability, presently engaged in a nonlimited work con- tract, and having given informed consent.*  *Exclusion criteria were LBP of specific origin (malignant, traumatic, infectious, or inflammatory LBP, acute sciatica, spondylolisthesis), recent spinal surgery (<4 months), cardiac or respiratory insufficiency (detected by stress tests), neurologic impairment, a psychiatric disorder precluding group therapy, and receiving disability pensions. A total of 132 patients (46 women and 86 men) were included and were randomized to either the AIT group (64 patients) or the FRP group (68 patients)."*  **Exp**;  n= mean age(sd) = 40.8 (7.4) 68% male  **control**:  n= mean age(sd) = 38.7 (6.1) 62% male |
| **Interventions** | **In/out:** Out  **Intensities**: high (150 hours)  **Group/individual:** Mixed  **Exp: (functional restorations)**  The FRP was performed 6 hours a day, 5 days a week, during 5 weeks, in 2 rehabilitation centers. Patients were treated in groups of 6 to 8.  **control**: (physio)  The AIT was composed exclusively of ambulatory physiotherapy, 1 hour 3 times a week, during 5 weeks, and was provided by a private-practice physiotherapist, |
| **Outcomes** | **Outcomes**  Number of sick days  Dallas Pain Questionnaire  trunk endurance and flexibility  Lifting capacity  Pain over last 24 hours  **Time points**: (short, Long)  Pre, post, 12 month FU |
| **Notes** |  |

Risk of bias table

| **Bias** | **Authors' judgement** | **Support for judgement** |
| --- | --- | --- |
| Random sequence generation (selection bias) | Low risk | "Patients were randomized by an independent methodologist to one of the 2 rehabilitation programs, according to an eight-element permutation table." |
| Allocation concealment (selection bias) | Low risk | "Patients were randomized by an independent methodologist to one of the 2 rehabilitation programs, according to an eight-element permutation table." |
| Blinding of participants and personnel (performance bias) | High risk | not possible due to differences between groups. |
| Blinding of outcome assessment (detection bias) | Unclear risk | information not included, however, as a self report measure is used to assess HRQoL, and no clear blinding of participants from group allocation has been described, there is an unclear risk of bias in this domain. |
| Incomplete outcome data (attrition bias) | High risk | "The main limitation of this study is the lost to follow-up rate. The outcome measures and specially the number of sick leaves were not available for these patients. Intention to treat analysis could not be performed" |
| Selective reporting (reporting bias) | Low risk | no protocol cited, however all measures from methods reported in results. |

Ronzi 2017

| **Methods** | **Title**: Efficiency of three treatment strategies on occupational and quality of life impairments for chronic low back pain patients: is the multidisciplinary approach the key feature to success?  A monocentric randomized controlled trial with a 12-months follow-up, conducted in the French Valley Loire region from May 2009 to April 2013.  **Location**: France |
| --- | --- |
| **Participants** | **Eligibility/recruitment:**  "*Participants were recruited from a French chronic LBP care-network involved in medical and occupational issues. This network includes a multidisciplinary clinic conducted by a rehabilitation physician, an occupational physician, a psychologist, and an occupational health nurse. Rehabilitation programs were provided in a rehabilitation center or by one of the private physio- therapists involved in the care-network. After attendance to the multidisciplinary clinic and randomization, participants were subsequently referred to the rehabilitation center or to a private physiotherapist of the region or both, depending on the arm of the trial.*  *Participants were subjects of working age suffering from non-specific chronic LBP according to the usually accepted scientific criteria.20 Patients could be included if they were aged 18 to 55, were able to sign the informed consent form, suffered from a non-specific LBP for at least three months without improvement which had led to at least one month’s sick leave during the preceding year and/or three months’ sick leave during the preceding two years, and were on an open-ended or a fixed-term contract of work in the public or private sector."*  **intervention group**  FRP : n=49 (mean age:40 55%male)  **control group**  AIP n=54 (mean age42 61% male)  **mixed**(lower intensity IMPT)  n=56 (mean age 40 62.5% male) |
| **Interventions** | **Out/in:** out  **group/individual:** mixed  **intensity**: mixed  FRP (intervention group) 150 hours  AIP (control group) 23 hours  mixed (lower intensity IMPT) 53 hours (approx.)  The three treatment strategies lasted five weeks, but the treatment content of each strategy was different.  **Exp**  Functional Restoration Program (FRP) involved six hours of treatment a day, five days a week in group of six to eight patients.  **Control** Ambulatory Individual Physiotherapy (AIP) included individual rehabilitation with a private physiotherapist for one hour, three times a week and individual exercises to be performed at home twice a week for 50 minutes.16,18  **Mixed**  The mixed strategy includes AIP combined with five one-day group sessions. Patients were off work during the five weeks of treatment in all the three groups. |
| **Outcomes** | **Outcome**  Number of days’ sick leave during the 12-months  SF-36 (PCS + MCS - no other sub scales)  Dallas Pain Questionnaire (DPQ)  standardized physical tests (Finger-Floor Distance (FFD),  Sorensen and Ito tests  visual analogic scale (VAS),  Fear Avoidance and Belief Questionnaire,  Hospital Anxiety and Depression scale  **time points**  Pre, 12 month follow-up |
| **Notes** | majority male sample  Chosen to include the high intensity data and active control as the comparison that will be included in the meta-analysis (as opposed to high vs med intensity). Best chance of detecting an effect and is a valid comparison to make within the parameters of the project. |

Risk of bias table

| **Bias** | **Authors' judgement** | **Support for judgement** |
| --- | --- | --- |
| Random sequence generation (selection bias) | Low risk | The randomization procedure of participants was determined by a computer randomization feature that automatically allocated the patient to one of the treatment strategies. |
| Allocation concealment (selection bias) | Low risk | "...feature that automatically allocated the patient to one of the treatment strategies." |
| Blinding of participants and personnel (performance bias) | High risk | Participants, therapists, and researchers could not be blinded for the allocated treatment after randomization. |
| Blinding of outcome assessment (detection bias) | High risk | An independent research assistant prepared envelopes and numbered them sequentially according to the randomization list. Envelopes were given to the rehabilitation physician who opened them at the end of the clinical evaluations described above. However, as a self report measure is used to assess HRQoL, and no clear blinding of participants from group allocation has been described, there is a clear risk of bias in this domain. |
| Incomplete outcome data (attrition bias) | Low risk | intention to treat |
| Selective reporting (reporting bias) | Low risk | all measures mentioned in materials reported in results. No protocol cited. |

Rooks 2007

| **Methods** | **Title**: Group Exercise, Education, and Combination Self-management in Women With Fibromyalgia  **Location**: USA |
| --- | --- |
| **Participants** | **Eligibility/recruitment**  "*Women 18 to 75 years of age with a confirmed diagnosis of fibromyalgia4 were recruited directly from physician practices (in the Boston area). Participants met the American College of Rheumatology criteria for fibromyalgia4; diagnosis was confirmed by the primary care physician of each participant. For participants in the care of a rheumatologist, we required agreement on the fibromyalgia diagnosis between physicians for enrolment."*  (1) aerobic and flexibility exercise (AE); **(control 1) (control group included in analysis model)**  allocated: n = 53 Mean age (sd)  completed: n = **35** Mean age (sd) 48 (11)  (2) strength training, aerobic, and flexibility exercise (ST); (**Control 2**)  allocated: n = 51Mean age (sd)  completed: n = **35** Mean age (sd) 50 (11)  (3) the Arthritis Foundation’s Fibromyalgia Self-Help Course (FSHC); (**low intensity intervention)**  allocated: n = 50 Mean age (sd)  completed: n = **27** Mean age (sd) 51 (12)  (4) a combination of ST and FSHC (ST-FSHC). (**Exp**) **(exp group included in analysis model)**  allocated: n = 55 Mean age (sd)  completed: n = **38** Mean age (sd) 50 (11) |
| **Interventions** | **In/out:** out  **Intensities**: high (100 hours)  **Group/individual:** group  All interventions were offered at 3 sites: 2 community fitness facilities outside Boston and the hospital wellness center. 16-week intervention.  **Exercise**;  Both exercise programs in this study involved approximately 60 minutes of activity per session. The 2 exercise groups met twice weekly on different days of the week (ie, Monday and Wednesday vs Tuesday and Thursday) to avoid group contamination. Written instructions were provided to all participants to perform a third day of exercise on their own. (estimated 48 hours total exercise) The FSHC is a 7-session program that teaches individuals with fibromyalgia about the condition and self-management skills.  Materials promoted basic self-management techniques to accomplish daily activities and manage symptoms and suggested ways to incorporate wellness activities, including exercise, into daily life. Information was provided through a series of lectures (5-15 minutes) with facilitated group discussion and supplementary readings.28 Sessions were 120 minutes long every 2 weeks. (56 hours total)  **EDUCATION**  The FSHC is a 7-session program that teaches individuals with fibromyalgia about the condition and self-management skills. Materials promoted basic self-management techniques to accomplish daily activities and manage symptoms and suggested ways to incorporate wellness activities, including exercise, into daily life. Information was provided through a series of lectures (5-15 minutes) with facilitated group discussion and supplementary readings.28 Sessions were 120 minutes long every 2 weeks. All FSHC instructors were certified by the Arthritis Foundation.  **COMBINATION**  Participants assigned to the combination group (ST-FSHC) participated in both the ST and FSHC groups’ activities. |
| **Outcomes** | **Outcomes**  FIQ  SF-36  Beck depression inventory  Fibromyalgia self efficacy questionnaire  **Timepoints**: (short, med)  Pre, post, 6 month follow-up |
| **Notes** | Authors seem to talk about 3 time points, but only seem to report 2 in the data (‘before’ and ‘after’ pre and 16 week follow-up). This creates some confusion as not explicitly clear if 'after' relates to 6 months, or directly after intervention completion. Based on the fact they provide conclusions about the outcomes at 6 months, it is deduced that this data must be for 6 months and not immediately post intervention.  four group in this study, the ST-FSHC was selected as the exp group due to giving most intense IMPT, AE selected as entirely separate group of ppts (as opposed to ST or FSCH groups)  all female sample |

Risk of bias table

| **Bias** | **Authors' judgement** | **Support for judgement** |
| --- | --- | --- |
| Random sequence generation (selection bias) | Low risk | Members of the hospital’s Biometrics Center not involved in the study used a computer program that generated single-page listings of random group assignment. Individual pages were placed in opaque envelopes, sealed, numbered sequentially, and stored in a locked cabinet |
| Allocation concealment (selection bias) | Low risk | see above |
| Blinding of participants and personnel (performance bias) | High risk | not possible due to group differences, *"participants assigned to the combination group (ST-FSHC) participated in both the ST and FSHC groups’ activities*." Participants in combination group would note the absence of ST participants and so know their own grouping |
| Blinding of outcome assessment (detection bias) | High risk | "Testers masked to participant group assignment administered all tests."  However, as a self report measure is used to assess HRQoL, and no clear blinding of participants from group allocation has been described, there is an unclear risk of bias in this domain. |
| Incomplete outcome data (attrition bias) | Low risk | Intention to treat applied |
| Selective reporting (reporting bias) | Low risk | No protocol cited, all measures from methods reported in results. |

Saral 2016

| **Methods** | **Title**: The effects of long‐ and short‐term interdisciplinary treatment approaches in women with fibromyalgia: a randomized controlled trial  **Location**: Turkey |
| --- | --- |
| **Participants** | **Eligibility/recruitment**  "inclusion criteria (i) women with FM, aged 25–60 years; (ii) diagnosis of FM according to the 1990 ACR diagnostic criteria [2]; (iii) followed up for at least six months after FM diagnosis; (iv) pain intensity of at least marked as five on the 10 cm visual analog scale (VAS) with 1-cm segments from 0 to 10, despite existing treatment; and (v) presence of at least five years of primary school education.  Exclusion criteria  (i) previous diagnosis of an endocrine, neuromuscular, infectious, or inflammatory disease;(ii) presence of hepatic or renal disease; (iii) malignancy;(iv) history of severe trauma; (v) advanced psychiatric diseases; (vi) serious physical comorbidities; and (vii) pregnancy."  "Eighty-six consecutive patients with FM, who were referred to our department, were recruited for the study. Among these patients, a total of 66 women who met the eligibility criteria were included in the study."  **Exp1**  the long-term interdisciplinary treatment group (LG, n = 22),  **exp 2**  the short-term interdisciplinary treatment group (SG, n = 22),  **control group**  (CG, n = 22) |
| **Interventions** | **In/out:** out  **Intensities**: Low, med  **Group/individual**: mixed  **LG**  participated in a 10-session extended CBT program (one 3-h session per week for 10 weeks), together with exercise training (one full day) and an educational program (one full day).  **SG**  received a compacted interdisciplinary treatment program that consisted of educational items, exercise training, and a brief CBT program over two consecutive days. The patients in the  **CG**  did not participate in any program and were advised to continue their previous treatments without any change. |
| **Outcomes** | **Outcomes**  VAS-Pain,  VAS-Fague,  VAS-Sleep,  Number of Tender Points,  Algometry,  FIQ,  Depressive Symptoms (BDI),  Quality of Life (SF-36)  **time points**: (med)  pre, 6 month follow-up |
| **Notes** | all female sample |

Risk of bias table

| **Bias** | **Authors' judgement** | **Support for judgement** |
| --- | --- | --- |
| Random sequence generation (selection bias) | Low risk | "According to the order of presentation to the outpatient clinic, eligible participants were randomly allocated to one of the three groups using a computer-generated random numbers program with an allocation ratio of 1:1:1 and without varying blocks": |
| Allocation concealment (selection bias) | Low risk | see quote above |
| Blinding of participants and personnel (performance bias) | High risk | No blinding was performed for the patients, outcome or data assessors |
| Blinding of outcome assessment (detection bias) | High risk | No blinding was performed for the patients, outcome or data assessors |
| Incomplete outcome data (attrition bias) | High risk | Statistical analyses were confined to the ‘per-protocol set’ (those who completed the trial in conformity with the study protocol) without any ‘intention-to-treat’ analysis due to the absence of any outcome measures after randomization/treatment for the withdrawals/dropouts. |
| Selective reporting (reporting bias) | Low risk | all measures mentioned in materials reported in results. No protocol cited. |

Tavafian 2008

| **Methods** | **Title**: A Randomized Study of Back School in Women With Chronic Low Back Pain  blind Randomised controlled trial  **Location**: Iran |
| --- | --- |
| **Participants** | Eligible participants were adult women recruited from outpatient rheumatology clinics. The selection criteria were: age 18 years and over, suffering from CLBP (persisting for 90 days or more). Exclusion criteria were having had back surgery within the 2 years before the initial observation, having complaint restricted to sacroiliac joint, cervical or thoracic regions and congenital spine disease  sample 100% female  Exp:  Allocated n=50  completed n=37 mean age (SD) 42.9 (10.7)  control:  Allocated n=52  completed n=37 mean age (SD) 44.7 (10.8) |
| **Interventions** | In/out: out  Intensity: Low (32 hours est.)  group/individual: group.  a 4-day, 5-session, multidimensional and interdisciplinary educational regime designed based on patients’ characteristics, lifestyle and subsequent ability to cope. (duration estimated at around 32 hours).  the control group (n 52) who received only medication under the supervision of the rheumatologist, and the exp group (n 50), who received the back school program in addition to medication. |
| **Outcomes** | SF-36  Timepoints;  baseline, 3,6,12 month follow-up |
| **Notes** |  |

Risk of bias table

| **Bias** | **Authors' judgement** | **Support for judgement** |
| --- | --- | --- |
| Random sequence generation (selection bias) | Low risk | *"The participants (n 102) were randomly assigned at the outset to the clinic group (n 52) who..."* |
| Allocation concealment (selection bias) | Low risk | *"The treatment allocation was not concealed and cointerventions were avoided for both group."* |
| Blinding of participants and personnel (performance bias) | High risk | *"Participants were not blinded to intervention."* |
| Blinding of outcome assessment (detection bias) | High risk | not clear, however self assessment and participants were not blind, so a high risk of bias |
| Incomplete outcome data (attrition bias) | High risk | attrition management not discussed |
| Selective reporting (reporting bias) | Low risk | all measures from methods reported in results. |

Tavafian 2011

| **Methods** | **Title**; Treatment of Chronic Low Back Pain A Randomized Clinical Trial Comparing Multidisciplinary Group-based Rehabilitation Program and Oral Drug Treatment With Oral Drug Treatment Alone  **Location**: Iran |
| --- | --- |
| **Participants** | **Eligibility/recruitment:**  "*Eligible patients were recruited from July 2008 to February 2009. Data were collected at the time of randomization (baseline), 3, and 6-month follow-ups. All CLBP patients aged Z18 years, with pain for more than 90 days, and referred to rheumatology clinics of 3 referral teaching hospitals and 1 private clinic were candidates for inclusion in the study. The primary causes of CLBP in this study were herniated intervertebral disc, facet arthropathy, and lumbar arthrosis. The 3 general hospitals of Shariati, Emam Khomeini, and Sina are affiliated with TUMS and located in center and south of Tehran where people from different sociodemographic classes live. The private clinic is located in center of Tehran near Shariati and Emam Khomeini hospitals.*  *Patients were not admitted to the study if any of the following criteria were present: back surgery within the past 2 years, recent vertebral fracture, vertebral malignancy, infection in the back, spondylolisthesis, spinal stenosis, inability to participate in the multidisciplinary program sessions, current pregnancy, residency outside of Tehran, insufficient address or phone number for follow-ups, difficulty understanding the Farsi language, or unwilling- ness to enter to the study or comply with the study protocols."*  197 patients with chronic low back pain were randomized to either  **intervention group**  (n=97) (mean age 44.6 (10.2) 73% female)  **control group**  (n=100) (mean age 45.9 (11.3) 83% female) |
| **Interventions** | **Intensity**; low  **In/out**: Out  **Group/individual:** mixed  **Exp**  *"The intervention was a group-based multidisciplinary rehabilitation program that acknowledged both biological and psychosocial aspects of CLBP and addressed these by different field specialists. This program involved five, 2-hour sessions followed by monthly booster sessions and monthly telephone counselling to encourage participants to maintain improved behaviours*."  **control group:** TAU/oral medication  "*The participants of both groups could see the physician earlier if they requested. Throughout the study, medications such as analgesics, nonsteroidal anti-inflammatory drugs, muscle relaxants, and antidepressant drugs were prescribed for patients in the 2 groups as needed. As the physician was blinded to the group assignment, the type and dosages of medications prescribed for both groups were the same and were based only on the clinical findings. Furthermore, during monthly phone counselling, the participants of both groups were encouraged to take their medications as prescribed by their physician. Prescribing opioids is illegal in Iran; therefore, these drugs were not prescribed for the participants of this study"* |
| **Outcomes** | **Outcomes**  The Short-form Health Survey (SF-36)  Ronald-Morris Disability Questionnaire (RDQ)  Quebec Back Pain Disability Scale (QDS)  **Timepoints**: short, intermediate  Baseline 3 months, 6 months |
| **Notes** | This study precedes and is aligned to the Tavafian 2017 paper below, its not been excluded because it provides the baseline values needed to include the 2017 source. The data has not been included twice or double counted in the analysis models.  While this study is comparing IMPT with pharmacological intervention (which differs from other studies included in this review, reading the details, its essentially just TAU (participants in other studies are anticipated to also have still had access to 'oral medications' (non opioid) throughout and so are comparable with the approach here). |

Risk of bias table

| **Bias** | **Authors' judgement** | **Support for judgement** |
| --- | --- | --- |
| Random sequence generation (selection bias) | Low risk | The person who was responsible for random allocation of the eligible selected patients was blind to the clinical and demographic characteristics of the patients. |
| Allocation concealment (selection bias) | Low risk | The sequence of allocation was concealed to the rheumatologist who selected the eligible patients. |
| Blinding of participants and personnel (performance bias) | High risk | "The patients were instructed to say nothing about their group assignment to the physician" |
| Blinding of outcome assessment (detection bias) | High risk | The physician and statistical analyst were blinded to the group assignment. The patients were instructed to say nothing about their group assignment to the physician.  As noted above participants were aware of their group assignment, and due to the use of self report measures to assess HRQoL there is a high risk of bias in this domain. |
| Incomplete outcome data (attrition bias) | Low risk | As there were not a considerable number of participants who did not fulfil the protocol of the study, no intention-to-treat analysis was performed. |
| Selective reporting (reporting bias) | Low risk | protocol not cited, however all measures mention in methods are reported in the results section |

Tavafian 2017

| **Methods** | **Title**: Treatment of low back pain: extended follow-up of an original trial (NCT00600197) comparing a multidisciplinary group-based rehabilitation program with oral drug treatment alone up to 30 months  **Location**: Iran |
| --- | --- |
| **Participants** | **Eligibility/recruitmen**t: (see Tavafian 2011 above for more details)  74% of patients (146/197) of the original study provided extended 30-month follow-up data.  **intervention**  n=69 (Mean age: 45.55 (9.60)) 78.3% female  **Control**  n=77 (mean age: 46.45 (11.45)) 83.1% female |
| **Interventions** | **Intensity**; low  **In/out:** Out  **Group/individual:** Group  **Exp**  The intervention was a group-based multidisciplinary rehabilitation program that acknowledged both biological and psychosocial aspects of CLBP and addressed these by different field specialists. This program involved five, 2-hour sessions followed by monthly booster sessions and monthly telephone counselling to encourage participants to maintain improved behaviours.  **control group**:  TAU/oral medication (see Tavafian 2011 above for more details) |
| **Outcomes** | **Outcomes**  The Short-form Health Survey (SF-36)  Ronald-Morris Disability Questionnaire (RDQ)  Quebec Back Pain Disability Scale (QDS)  **Timepoints**:  30 month follow-up |
| **Notes** | very long-term follow-up compared to many other sources included in the long-term follow-up category |

Risk of bias table

| **Bias** | **Authors' judgement** | **Support for judgement** |
| --- | --- | --- |
| Random sequence generation (selection bias) | Low risk | "Participants were randomly assigned into the intervention or control group through random permutation blocking of every 6 participants. This kind of randomization was used to ensure close balance of numbers in each group." |
| Allocation concealment (selection bias) | Low risk | *"The sequence of allocation was concealed to the rheumatologist who selected the eligible patients. The person who was responsible for random allocation of the eligible selected patients was blind to the clinical and demographic characteristics of the patients."* |
| Blinding of participants and personnel (performance bias) | High risk | *"Due to the nature of the intervention, full blinding of patients was impractical."* |
| Blinding of outcome assessment (detection bias) | High risk | "*The patients were instructed to say nothing about their group assignment to the physician. However, no further measures were undertaken to insure blinding of the physician."*  *from 2011 paper:*  "The physician and statistical analyst were blinded to the group assignment. The patients were instructed to say nothing about their group assignment to the physician."  As a self report measure is used to assess HRQoL, and no clear blinding of participants from group allocation has been possible, there is a high risk of bias in this domain. |
| Incomplete outcome data (attrition bias) | High risk | intention to treat not applied |
| Selective reporting (reporting bias) | Low risk | measures identified in protocol are reported in the paper |

Taylor 2016

| **Methods** | **Titl**e: Novel Three-Day, Community-Based, Nonpharmacological Group Intervention for Chronic Musculoskeletal Pain (COPERS): A Randomised Clinical Trial  **Location:** England |
| --- | --- |
| **Participants** | **Eligibility/recruitment**  We conducted a pragmatic, multi-centre randomised controlled trial of the COPERS group self-management course for adults living with chronic musculoskeletal pain. Causes of pain included, but were not restricted to, osteoarthritis, back pain, chronic widespread pain, and fibromyalgia. Participants were recruited in the UK (London and the Midlands) from primary care, community musculoskeletal pain services, and secondary care pain services.  Between August 1, 2011, and July 31, 2012, we randomised 703 participants from 35 general practices, two secondary care pain services, and one community-based musculoskeletal service  Most of the participants (85%) had had pain for at least 3 y, with 265 (38%) reporting pain for more than 10 y and 162 (23%) being prescribed strong opioids (as defined in the British National Formulary [40]) at baseline. The median number of comorbidities (determined from primary care records) was 2 (range 0–8). Only 169 (24%) participants were in any form of employment, with 148 (21%) who were unable to work due to long-term sickness and another 307 (44%) who were retired.  **Intervention**  n=403 mean age: 60.3 (13.5) :33% male  **control**  n=300 (mean age: 59.4 (13.8) 33% male). |
| **Interventions** | **In/out**: Out  **Intensity**: low  **Group**/**individual**: group  Courses were delivered by two facilitators: a health care professional with experience treating people with chronic musculoskeletal pain (physiotherapist, psychologist, osteopath, or GP) and a lay person living with chronic pain  **Exp group:**  delivered in a community setting over three alternate days in 1 wk, with a follow-up session 2 wk later (total duration = 14 h). Content included cognitive behavioural approaches to managing chronic pain (these covered acceptance, attention control, goal setting and action planning.  **control**  usual care (including a widely available pain education leaflet: and a relaxation CD (also given to intervention participants). To mimic the duration of the intervention, control participants were asked to practise relaxation daily for 3 wk and whenever they wished thereafter. |
| **Outcomes** | **Outcomes**:  Chronic Pain Grade [CPG] disability subscale);  Hospital Anxiety and Depression Scale [HADS]),  pain acceptance (Chronic Pain Acceptance Questionnaire), social integration (Health Education Impact Questionnaire social integration and support subscale),  pain-related self-efficacy (Pain Self-Efficacy Questionnaire),  pain intensity (CPG pain intensity subscale),  the census global health question (2011 census for England and Wales),  health utility (EQ-5D-3L),  health care resource use.  **time point**s: (med, long)  baseline, 6 months, 12 months |
| **Notes** |  |

Risk of bias table

| **Bias** | **Authors' judgement** | **Support for judgement** |
| --- | --- | --- |
| Random sequence generation (selection bias) | Low risk | Following the return of completed baseline questionnaires, participants were randomised to the two groups in a 1.33:1 ratio in favour of the intervention arm. |
| Allocation concealment (selection bias) | Low risk | Strict allocation concealment was maintained via an independent, centralised online service that used stratified permuted blocks with randomly varying block sizes of 7 or 14 and recruitment site as a stratification factor. |
| Blinding of participants and personnel (performance bias) | High risk | it was not feasible to mask participants or group facilitators to study arm. |
| Blinding of outcome assessment (detection bias) | High risk | Participants’ health care professionals and all those retrieving, handling, or processing outcome data remained unaware of participants’ allocated study arms.  However, as a self report measure is used to assess HRQoL, and no clear blinding of participants from group allocation has been possible, there is a high risk of bias in this domain. |
| Incomplete outcome data (attrition bias) | Low risk | intention to treat and also Multiple imputation methods applied |
| Selective reporting (reporting bias) | Low risk | study adheres to protocol:  https://www.ncbi.nlm.nih.gov/pubmed/23358564 |

van der Hulst 2008

| **Methods** | **Title**: Multidisciplinary Rehabilitation Treatment of Patients With Chronic Low Back Pain: A Prognostic Model for Its Outcome (see notes)  **Location:** Holland |
| --- | --- |
| **Participants** | **Eligibility/recruitment**  "*All patients who were admitted to the back rehabilitation program met the inclusion criteria, of which <5% refused to participate. Of the 163 patients who were included in the trial, 21 patients were lost during follow-up (13%). There was no difference in loss to follow-up between the groups.*"  **MBI**  n= 79 (mean age: 38 (10) 60% male)  **Usual** **care**  n=84 (mean age: 40 (10) 62% male) |
| **Interventions** | Out/in: **Out**  Intensity: **med** (**42h**)  Individual/group: **mixed**  **MBI:**  Patients who were allocated to the treatment group began participating in the Roessingh Back Rehabilitation Program (RRP) within 2 to 3 weeks. The RRP was based on the Swedish back school22 and multidimensional pain programs. The treatment program consists of a combination of physiotherapy, sport, education, and occupational rehabilitation. The RRP is provided on the basis of a standardized protocol. Patients are not allowed to be absent more than 10% of the time. An RRP group consists of 8 patients and comprises 3 hours of conditional training and sport, 0.5 hours of swimming, 1.5 hours of occupational therapy, and 4 hours of physiotherapy each week for 7 weeks. Treatment is under the supervision of a specialist in physical and rehabilitation medicine and conducted by a team consisting of a physiotherapist, an occupational therapist, a sport therapist, and, if necessary, a psychologist and a dietician. **(42 hours total**)  **Usual care:**  Patients randomized to the control group (ie, waiting list) were allowed to apply for usual health care facilities outside the rehabilitation centre. The medical consumption of the control group was assessed at T1 and every subsequent month till T5 by a questionnaire that was sent home. They could enter the back rehabilitation program after the 6-month follow-up period. |
| **Outcomes** | **Outcomes**  pain intensity VAS  Work status  multidimensional pain inventory  sick leave  receiving financial compensation  Symptom Checklist-90 subscale depression (SCL-90-Dep)  Tampa Scale of Kinesiophobia-Dutch Version(TSK-DV,)  Roland morris disability questionnaire  Sf-36  **Time points:** (short, Long**)**  Baseline, 8 weeks, 6 month follow-up |
| **Notes** | study aims to develop a prognostic model using data from an earlier RCT (Vollenbroek-Hutten 2004 ). To avoid duplication this earlier study has not been included. The current study was preferred due to its reporting of the SF-36 and homogeneity of measure (which was omitted from the original study).  majority male sample. |

Risk of bias table

| **Bias** | **Authors' judgement** | **Support for judgement** |
| --- | --- | --- |
| Random sequence generation (selection bias) | Low risk | *"Randomization was performed using the minimization method as described by Pocock20 and balanced for sex, work status, and low back muscle function as estimated by dynamometry (by using the Isostation B200)."* |
| Allocation concealment (selection bias) | Low risk | *"To enable an adequate assignment procedure, a computer program was used.*" |
| Blinding of participants and personnel (performance bias) | High risk | *"Patients were not blinded for the group they were randomized to, but the researchers conducting the measurements were*" |
| Blinding of outcome assessment (detection bias) | High risk | Patients were not blinded for the group they were randomized to, but the researchers conducting the measurements were. However, as a self report measure is used to assess HRQoL, and no clear blinding of participants from group allocation has been described, there is a high risk of bias in this domain. |
| Incomplete outcome data (attrition bias) | Low risk | Under the hypothesis that the data were missing at random, multiple imputation techniques were used |
| Selective reporting (reporting bias) | High risk | As noted above in the 'notes' section. this study uses data gathered from an earlier RCT. Selective reporting of measures is present in both. |

Van der Maas 2015

| **Methods** | **Title**: Improving the Multidisciplinary Treatment of Chronic Pain by Stimulating Body Awareness Cluster-randomized Trial  **Location**: Holland |
| --- | --- |
| **Participants** | **Eligibility/recruitment**  Patients with chronic musculoskeletal pain, such as nonspecific low back pain, post whiplash syndrome, and fibromyalgia, who were referred for pain rehabilitation treatment to an outpatient centre for pain rehabilitation in Amsterdam,  **Exp**:  PMT n= 49 (mean age: 38.6 (11.1). 91% female)  **Control**  TAU n= 45 (mean age: 45.4 (11.1). 71% female) |
| **Interventions** | **In/out:** out  **Intensity:** Med and high  **Group/individual:** mixed  **TAU+group PMT (Exp - this group has standard IMPT + extra PMT)**  Patients in the TAU+group PMT received 10 sessions of psychomotor training 1.5 hours in addition to TAU  **MBI** (Treatment as usual):  multi-component group treatment package and included the following elements:  relaxation21 (61.5 h),  graded activity based on the program of Fordyce et al22 on 1 aerobic fitness device (331 h),  rational-emotive therapy23 (91h, 61.5 h),  occupational therapy (61.5 h),  chronic pain education (31.5 h),  sports (in the swimming pool [51 h]  and in the sports hall [51 h]),  partner education (31.5 h),  and coaching (41 h),  - a total of 94 hours.  The different components of treatment were spread across 3 d/wk during 12 weeks. Two follow-up group sessions of 1.5 hours were offered after 3 and 6 months. |
| **Outcomes** | **Outcomes**  pain intensity (11 point scale)  SF-36  Pain Disability Index (Dutch version)  Beck Depression Inventory  Scale of Body Connection  Pain Self-Efficacy Questionnaire  The Pain Catastrophizing Scale  **Timepoints**: (short, med, long)  Pre, post, 3 month follow-up, 6 month follow-up. 12month |
| **Notes** | Study is examining the impact of adding 'Psycho motor treatment' to pre existing IMPT. technically this should be included based on the new criterion for included studies. the two groups straddle the medium and high intensity threshold. 94 hours and 109 hours. |

Risk of bias table

| **Bias** | **Authors' judgement** | **Support for judgement** |
| --- | --- | --- |
| Random sequence generation (selection bias) | Low risk | *"Cluster randomization was used to assign treatment groups of 4 to 6 patients to one of 2 multidisciplinary treatment interventions,"* |
| Allocation concealment (selection bias) | Unclear risk | not explicitly stated |
| Blinding of participants and personnel (performance bias) | High risk | *"The patients and therapists were aware of the treatment group assignment"* |
| Blinding of outcome assessment (detection bias) | High risk | information not included. However, as a self-report measure is used to assess HRQoL, and no clear blinding of participants from group allocation has been described, there is a high risk of bias in this domain. |
| Incomplete outcome data (attrition bias) | Low risk | intention to treat. |
| Selective reporting (reporting bias) | Low risk | all measures mentioned in materials reported in results. No protocol cited. |

Westman 2010

| **Methods** | **Title**: Controlled 3-year follow-up of a multidisciplinary pain rehabilitation program in primary health care  **Location**: Sweden |
| --- | --- |
| **Participants** | **Eligibility/recruitment**  *"During the period 1998–2000, a rehabilitation project was carried out in primary health care in the county of Va ̈stmanland, Sweden. One hundred and fifty-eight consecutive patients with musculoskeletal pain were recruited to the project. It was emphasised that participation was voluntary and had no negative consequences for their insurance status. Eighty-nine patients (experimental group) from two primary health care units received different treatment interventions after a multidisciplinary investigation; Sixty-nine patients (control group) from four other primary health care units, fulfilling the same inclusion criteria, received routine treatment. Inclusion criteria were patients with musculoskeletal pain, age 18–65 years, sick leave between 28 and 180 days and/or consultation of the general practitioner about the same problem three times during the last 12 months. The participants were either in permanent employment or seeking work. Patients requiring orthopaedic surgery and those with a psychiatric disorder or substance abuse problem were excluded. Furthermore, participants had to be able to speak Swedish sufficiently well to be able to describe their symptoms and understand the in- formation given."*  (data for for sf36 measure, some variation in sample sizes for other outcomes)  **exp:**  n=89 (mean age: 46 (10.3) m/f: 25/64  **Con:**  n=69 (mean age: 47 (9.3) m/f: 23/46 |
| **Interventions** | **out/in:** Out  **Intensity:** Med  **Individual/group:** mixed  **Experimental** group. The patients were offered a multidisciplinary programme including one or more of the following interventions The team met once a week and suggested the kind of treatment the patient was to be offered:  1. 6-week group rehabilitation programme (41 patients) 2. Three-way communication – patient/general practitioner/psychologist (or social worker) (24 patients) 3. Individual treatment:. physiotherapy (79 patients) short-term psychotherapy (44 patients) 4. Workplace-based intervention (38 patients)  ***However***: results reports this as 1 group not 4.  *"The group program lasted for six consecutive weeks, in groups of 6–8 patients, with 4-h sessions 4 days per week. The sessions consisted of physical training, body awareness exercises, relaxation training and creative activities (e.g. music, dance, art)." This equals around 96 hours*  **Control**  *"The patients in the control group received routine treatment from the general practitioner. In Sweden, most patients with long-lasting musculoskeletal pain receive medication and general advice from general practitioners. They may also be referred to a physiotherapist or chiropractor/napra- path and, occasionally, for orthopaedic consultation."* |
| **Outcomes** | Work capacity/sick leave  SF-36  Job strain  Coping strategies Questionnaire  Pain Catastrophising Scale  Tampa Scale for Kinesiophobia  Health care utilisation  Drug consumption  Psychosomatic symptoms  Questions about pain  **time points**: (long)  pre, 3 year follow-up |
| **Notes** | See intervention information about breadth of ppts in groups and how outcomes were combined to a single group for reporting outcomes. |

Risk of bias table

| **Bias** | **Authors' judgement** | **Support for judgement** |
| --- | --- | --- |
| Random sequence generation (selection bias) | High risk | *"it was deemed neither suitable nor possible to randomise patients to the different primary health care units. To keep the personnel and the participating patients ‘clean’ in the experimental sense, we chose instead to select the controls from four other primary health care units in the region where traditional care of pain patients was practiced."* |
| Allocation concealment (selection bias) | Unclear risk | information not included |
| Blinding of participants and personnel (performance bias) | High risk | not possible due to difference in experimental groups |
| Blinding of outcome assessment (detection bias) | High risk | Information not included, However, as a self report measure is used to assess HRQoL, and no clear blinding of participants from group allocation has been possible, there is a high risk of bias in this domain. |
| Incomplete outcome data (attrition bias) | Unclear risk | drop outs mentioned but data approach not explained.  *"At 3-year follow-up there was 10 drop-outs. Drop- out analysis showed a sex difference with 72% of the study group being female when compared with 60% among the drop-outs*." |
| Selective reporting (reporting bias) | Low risk | all measures mentioned in materials reported in results. No protocol cited. |

Footnotes

##

**Characteristics of excluded studies**

Abbasi 2012

| **Reason for exclusion** | No QoL Measure |
| --- | --- |

Abbey 2013

| **Reason for exclusion** | No comparison/control group |
| --- | --- |

Abrams 2013

| **Reason for exclusion** | No comparison/control group |
| --- | --- |

AlAujan 2016

| **Reason for exclusion** | Protocol rather than actual study |
| --- | --- |

Allaire 2017

| **Reason for exclusion** | Review article |
| --- | --- |

Allaire 2018

| **Reason for exclusion** | No comparison/control group |
| --- | --- |

Amstel 2018

| **Reason for exclusion** | Intervention not biopsychosocial |
| --- | --- |

Anagnostis 2003

| **Reason for exclusion** | No QoL |
| --- | --- |

Anderson 2006

| **Reason for exclusion** | Study focuses on follow-up intervention rather than IMPT directly |
| --- | --- |

Anderson 2007

| **Reason for exclusion** | Intervention not biopsychosocial |
| --- | --- |

Anderson 2007a

| **Reason for exclusion** | Not directly concerning a MM pain programme |
| --- | --- |

Angst 2006

| **Reason for exclusion** | No comparison/control group |
| --- | --- |

Artacho-Cordón et al 2023

| **Reason for exclusion** | Intervention not delivered by multidisciplinary team |
| --- | --- |

Asenlöf 2005

| **Reason for exclusion** | No QoL |
| --- | --- |

Baranoff 2014

| **Reason for exclusion** | No QoL  Uses a scale called ‘Chronic Pain acceptance questionnaire’ and reports that acceptance is related to HrQoL measures. + the scale has convergent validity with the SF-36 http://liu.diva-portal.org/smash/get/diva2:690738/FULLTEXT01.pdf  however after close inspection its not convergent enough. |
| --- | --- |

Baranoff 2016

| **Reason for exclusion** | No comparison/control group |
| --- | --- |

Bauer 2016

| **Reason for exclusion** | No QoL |
| --- | --- |

Becker 2001

| **Reason for exclusion** | Duplicate data from Becker 2000 |
| --- | --- |

Bendix 1995

| **Reason for exclusion** | No QoL |
| --- | --- |

Bendix 1996

| **Reason for exclusion** | No QoL |
| --- | --- |

Bendix 1997

| **Reason for exclusion** | No QoL |
| --- | --- |

Bendix 1998

| **Reason for exclusion** | No Qol measure |
| --- | --- |

Bendix 1998a

| **Reason for exclusion** | No Qol measure |
| --- | --- |

Bendix 2000

| **Reason for exclusion** | No QoL measure |
| --- | --- |

Bergström 2012

| **Reason for exclusion** | No QoL |
| --- | --- |

Bertsche 2009

| **Reason for exclusion** | Cancer patients |
| --- | --- |

Bierner 2010

| **Reason for exclusion** | Sample not CP patients |
| --- | --- |

Bileviciute‐Ljungar 2014

| **Reason for exclusion** | No QoL |
| --- | --- |

Björnsdóttir 2018

| **Reason for exclusion** | no control group, two experimental groups. Both groups are IMPTs of the same intensity so can't compare. |
| --- | --- |

Bliokas 2007

| **Reason for exclusion** | No QoL |
| --- | --- |

Borys 2015

| **Reason for exclusion** | Marburg Questionnaire on Habitual Well-being not suitable HRQoL measure  Its noted in Donath, C., Geiß, C., & Schön, C. (2018). Validation of a core patient-reported-outcome measure set for operationalizing success in multimodal pain therapy: useful for depicting long-term success?. BMC health services research, 18(1), 1-13.  Who cite (in german):  Nagel B, Pfingsten M, Lindena G, Nilges P. Deutscher Schmerzfragebogen. Handbuch. Berlin: Deutsche Schmerzgesellschaft e.V; 2012.  Stating that the Marburg Questionnaire on Habitual Well-being “The construct has been reported to be only moderately correlated with Quality of Life and the DASS variables [5]. Thus, the authors concluded that it offers additional information”  For this reason it was concluded that no meaningful measure of HRQoL has been induced in this paper. |
| --- | --- |

Boyers 2013

| **Reason for exclusion** | Review article |
| --- | --- |

Brendbekken 2016

| **Reason for exclusion** | No QoL |
| --- | --- |

Brendbekken 2017

| **Reason for exclusion** | No QoL |
| --- | --- |

Brox 2003

| **Reason for exclusion** | No QoL |
| --- | --- |

Brox 2006

| **Reason for exclusion** | No QoL |
| --- | --- |

Brox 2010

| **Reason for exclusion** | No Qol |
| --- | --- |

Bults et al 2023

| **Reason for exclusion** | study frames itself as different from other PMP evaluations in this review due to it focusing on delivering their intervention in a 'primary' rather than 'secondary' or 'tertiary' healthcare setting. Excluded to avoid conflating effects between different health environments and approaches. Further to this, participants receive individual diagnosis and treatment, there is no group element, and there is no set ‘programme’ or intervention to be assessed. |
| --- | --- |

Burckhardt 1994

| **Reason for exclusion** | insufficient data included in paper for analysis. Author contacted but had nothing further to offer. |
| --- | --- |

Busch 2011

| **Reason for exclusion** | No QoL |
| --- | --- |

Busschbach 2012

| **Reason for exclusion** | Sample not restricted to CP |
| --- | --- |

Caby 2016

| **Reason for exclusion** | despite the title of this paper, there is no control/comparison group |
| --- | --- |

Carbonell-Baeza 2010

| **Reason for exclusion** | No QoL |
| --- | --- |

Cardosa 2012

| **Reason for exclusion** | No QoL |
| --- | --- |

Cederbom 2019

| **Reason for exclusion** | Intervention not carried out by multidisciplinary team. |
| --- | --- |

Chan 2013

| **Reason for exclusion** | No QoL |
| --- | --- |

Christiansen 2015

| **Reason for exclusion** | No QoL |
| --- | --- |

Cipher 2007

| **Reason for exclusion** | Sample ailments too broad |
| --- | --- |

Coleman 2012

| **Reason for exclusion** | No QoL |
| --- | --- |

Coleman 2012a

| **Reason for exclusion** | duplicate record |
| --- | --- |

Cormier 2016

| **Reason for exclusion** | No comparison/control group |
| --- | --- |

Cuesta-Vargas 2013

| **Reason for exclusion** | No comparison/control group |
| --- | --- |

Cuesta-Vargas 2015

| **Reason for exclusion** | No comparison/control group |
| --- | --- |

Cunningham 2011

| **Reason for exclusion** | Protocol |
| --- | --- |

Dahl 2004

| **Reason for exclusion** | Intervention not Biopsychosocial |
| --- | --- |

Dalewski 2019

| **Reason for exclusion** | Intervention is not PMP |
| --- | --- |

Darchuk 2010

| **Reason for exclusion** | No comparison/control group |
| --- | --- |

Darnall 2014

| **Reason for exclusion** | No QoL |
| --- | --- |

De Andres 2017

| **Reason for exclusion** | Intervention not Biopsychosocial |
| --- | --- |

Dekker 2016

| **Reason for exclusion** | Protocol |
| --- | --- |

Demoulin 2006

| **Reason for exclusion** | No comparison/control group |
| --- | --- |

Demoulin 2010

| **Reason for exclusion** | exp group are measured a pre, 18 weeks and 32 weeks. control are only measured twice at 4 week intervals. Due to the large difference in these conditions it was deemed that accurate comparison between these scores would not be valid. |
| --- | --- |

Dengler 2017

| **Reason for exclusion** | the 'control' group is the IMPT for this study, and while some patients had access to CBT not all did and most only had some pharma optimisation and physio (therefore not qualifying as IMPT consistently) |
| --- | --- |

Devan 2018

| **Reason for exclusion** | Review article |
| --- | --- |

Dobscha 2009

| **Reason for exclusion** | Intervention team not multidisciplinary professionals |
| --- | --- |

Doleys 2006

| **Reason for exclusion** | Study only reports post outcomes for QoL measures. Authors were contacted but reported that no further data was available. |
| --- | --- |

Dong 2019

| **Reason for exclusion** | Not RCT |
| --- | --- |

Durmus 2013

| **Reason for exclusion** | No QoL + is a conference abstract |
| --- | --- |

Dysvik 2004

| **Reason for exclusion** | No comparison/control group |
| --- | --- |

Dysvik 2005

| **Reason for exclusion** | No comparison/control group |
| --- | --- |

Dysvik 2012

| **Reason for exclusion** | inadequate control condition |
| --- | --- |

Eaton 2014

| **Reason for exclusion** | not face to face |
| --- | --- |

Elsesser 2017

| **Reason for exclusion** | Intervention not Biopsychosocial |
| --- | --- |

Erp 2015

| **Reason for exclusion** | Protocol |
| --- | --- |

Ersek 2008

| **Reason for exclusion** | No QoL |
| --- | --- |

Esmer 2010

| **Reason for exclusion** | Intervention and delivery team not multidisciplinary enough. |
| --- | --- |

Fairbank 2005

| **Reason for exclusion** | Surgical intervention vs IMPT, no comparison to be made. |
| --- | --- |

Finney 2016

| **Reason for exclusion** | Review article |
| --- | --- |

Fischer 2014

| **Reason for exclusion** | No comparison/control group |
| --- | --- |

Fullen 2014

| **Reason for exclusion** | No QoL |
| --- | --- |

Furunes 2017

| **Reason for exclusion** | Surgical intervention vs IMPT, no comparison to be made. |
| --- | --- |

Furunes 2017a

| **Reason for exclusion** | duplicate |
| --- | --- |

Garcia 2013

| **Reason for exclusion** | intervention group are too similar to allow comparison. |
| --- | --- |

Garland 2014

| **Reason for exclusion** | No QoL |
| --- | --- |

Garschagen 2015

| **Reason for exclusion** | Review article |
| --- | --- |

Gerdle 2016

| **Reason for exclusion** | No comparison/control group |
| --- | --- |

Ghadyani 2016

| **Reason for exclusion** | No QoL measure. |
| --- | --- |

Ghelman 2020

| **Reason for exclusion** | Not PMP |
| --- | --- |

Gianola 2018

| **Reason for exclusion** | Review article |
| --- | --- |

Glombiewski 2018

| **Reason for exclusion** | not PMP |
| --- | --- |

Goertz 2017

| **Reason for exclusion** | This paper only takes a post measure for HRQoL, this means it is not possible to ascertain a pre post measure and an overall measure of change. |
| --- | --- |

Goossens 1998

| **Reason for exclusion** | The measure used in this study is a translation of another measure. The translation is not validated in anyway and therefore is not a reliable scale for QoL |
| --- | --- |

Goossens 2005

| **Reason for exclusion** | QoL not reported directly |
| --- | --- |

Gowans 1999

| **Reason for exclusion** | No QoL |
| --- | --- |

Grant 1995

| **Reason for exclusion** | Review article |
| --- | --- |

Guerriere 2010

| **Reason for exclusion** | No QoL |
| --- | --- |

Gunreben-Stempfle 2009

| **Reason for exclusion** | No QoL |
| --- | --- |

Gustafsson 2002

| **Reason for exclusion** | There is insufficient reporting of data in the original paper, and authors are not able to provide any further detail that would enable it to be included in the analysis.  pre post and change data not reported in any format for the QoL scale, just some p values. |
| --- | --- |

Gustafsson 2014

| **Reason for exclusion** | No QoL |
| --- | --- |

Guzman 2001

| **Reason for exclusion** | Review article |
| --- | --- |

Hadlandsmyth 2016

| **Reason for exclusion** | Intervention not biopsychosocial |
| --- | --- |

Hagen 2015

| **Reason for exclusion** | Review article |
| --- | --- |

Haldorsen 2002

| **Reason for exclusion** | No QoL |
| --- | --- |

Hallstam 2016

| **Reason for exclusion** | No comparison/control group |
| --- | --- |

Hallstam 2017

| **Reason for exclusion** | Intervention not biopsychosocial |
| --- | --- |

Hamnes 2012

| **Reason for exclusion** | No QoL measure |
| --- | --- |

Hansen 2017

| **Reason for exclusion** | Intervention not Biopsychosocial |
| --- | --- |

Härkäää 1996

| **Reason for exclusion** | No QoL |
| --- | --- |

Hausmann 2017

| **Reason for exclusion** | Intervention not Biopsychosocial |
| --- | --- |

Heapy 2007

| **Reason for exclusion** | Intervention not Biopsychosocial |
| --- | --- |

Heiskanen 2012

| **Reason for exclusion** | No comparison/control group |
| --- | --- |

Hellum 2011

| **Reason for exclusion** | Surgical intervention vs IMPT, no comparison to be made. |
| --- | --- |

Henriksson 2004

| **Reason for exclusion** | No comparison/control group |
| --- | --- |

Hernandez-Lucas et al 2023a

| **Reason for exclusion** | Intervention not delivered by multidisciplinary team |
| --- | --- |

Hernandez-Lucas et al 2023b

| **Reason for exclusion** | Intervention not delivered by multidisciplinary team |
| --- | --- |

Heutink 2012

| **Reason for exclusion** | has a ‘life satisfaction’ measure, had initially thought could be proxy for QoL but as per A.Bowling 2017 Measuring Heath. This is not accurate |
| --- | --- |

Heutink 2014

| **Reason for exclusion** | Duplication of Heutink 2014a (see included studies) + is a thesis rather than publication. |
| --- | --- |

Heutink 2014a

| **Reason for exclusion** | while this is a follow-up on Heutink 2012, the new follow-up data is only for the experimental and not the control group. |
| --- | --- |

Hoon 2017

| **Reason for exclusion** | No comparison/control group |
| --- | --- |

Hoon 2017a

| **Reason for exclusion** | No QoL |
| --- | --- |

Huffman 2017

| **Reason for exclusion** | No QoL |
| --- | --- |

Huge 2006

| **Reason for exclusion** | The control group in this study had individual multidisciplinary support plans designed for them. These varied based on need with respect to duration, content and intensity. Due to the inability to differentiate between the intervention in the control and exp conditions it is not possible to include this study. |
| --- | --- |

Hutting 2015

| **Reason for exclusion** | Intervention not biopsychosocial or lead by multidisciplinary team |
| --- | --- |

Jeitler 2015

| **Reason for exclusion** | intervention not biopsychosocial |
| --- | --- |

Jensen 1997

| **Reason for exclusion** | Group intervention conditions too similar |
| --- | --- |

Jensen 2005

| **Reason for exclusion** | They report a global sf 36 score by taking an average of the 8 sub scales, however this isn’t a valid way to process the data. In a purist sense it can’t be included, as; If the tools are misused, then the data can’t be considered ‘valid’ in a statistical sense. |
| --- | --- |

Jensen 2006

| **Reason for exclusion** | Intervention not biopsychosocial |
| --- | --- |

Johnsen 2014

| **Reason for exclusion** | Surgical intervention vs IMPT, no comparison to be made. |
| --- | --- |

Joos 2004

| **Reason for exclusion** | No comparison/control group |
| --- | --- |

Kaiser 2017

| **Reason for exclusion** | Review article |
| --- | --- |

Karttunen 2014

| **Reason for exclusion** | No QoL |
| --- | --- |

Karttunen 2015

| **Reason for exclusion** | No QoL |
| --- | --- |

Keel 1997

| **Reason for exclusion** | Not English language |
| --- | --- |

Kell 2011

| **Reason for exclusion** | Intervention not Biopsychosocial |
| --- | --- |

Keller 1997

| **Reason for exclusion** | excluded because it looks at 'wellbeing' rather than QoL. This is a separate construct. |
| --- | --- |

Kerkvliet 2008

| **Reason for exclusion** | No QoL |
| --- | --- |

Koleck 2006

| **Reason for exclusion** | No intervention |
| --- | --- |

Kole-Snijders 1999

| **Reason for exclusion** | No QoL |
| --- | --- |

Kopkow 2016

| **Reason for exclusion** | Review article |
| --- | --- |

Kroenke 2014

| **Reason for exclusion** | Not face to face (telecare) |
| --- | --- |

Kroese 2009

| **Reason for exclusion** | No comparison/control group |
| --- | --- |

la Cour 2015

| **Reason for exclusion** | intervention not delivered by multidisciplinary team. |
| --- | --- |

Lamb 2010

| **Reason for exclusion** | intervention isn't biopsychosocial |
| --- | --- |

Lamb 2010a

| **Reason for exclusion** | Duplicate of Lamb 2010 |
| --- | --- |

Landstrom 2017

| **Reason for exclusion** | Conference abstract |
| --- | --- |

LeFort 1998

| **Reason for exclusion** | not delivered by multidisciplinary team |
| --- | --- |

Lemstra 2002

| **Reason for exclusion** | No QoL |
| --- | --- |

Lemstra 2005

| **Reason for exclusion** | No QoL |
| --- | --- |

Leroux 2018

| **Reason for exclusion** | Intervention not delivered by multidisciplinary team |
| --- | --- |

Li 2006

| **Reason for exclusion** | Sample is 'work related injuries and long-term leave' rather than chronic pain. |
| --- | --- |

Linden 2014

| **Reason for exclusion** | Intervention not Biopsychosocial |
| --- | --- |

Linton 2000

| **Reason for exclusion** | Sample is acute rather than chronic pain |
| --- | --- |

Linton 2001

| **Reason for exclusion** | No QoL |
| --- | --- |

Linton 2006

| **Reason for exclusion** | intervention not biopsychosocial |
| --- | --- |

Ljungkvist 2000

| **Reason for exclusion** | no comparison/control group |
| --- | --- |

Lopes 2019

| **Reason for exclusion** | Not RCT |
| --- | --- |

Luciano 2011

| **Reason for exclusion** | Not delivered by multidisciplinary team |
| --- | --- |

Lynch 1996

| **Reason for exclusion** | No QoL |
| --- | --- |

Magnusson 2004

| **Reason for exclusion** | Insufficient reporting in original source to include in analysis. Repeated attempts to contact the authors for further detail were unsuccessful. |
| --- | --- |

Majeed 2018

| **Reason for exclusion** | Review article |
| --- | --- |

Manca 2010

| **Reason for exclusion** | Intervention not Biopsychosocial |
| --- | --- |

Mangels 2009

| **Reason for exclusion** | 3 interventions are all high intensity, no control/comparison can be made. |
| --- | --- |

Mannerkorpi 2002

| **Reason for exclusion** | No comparison/control group |
| --- | --- |

Mannion 2013

| **Reason for exclusion** | Surgical intervention vs IMPT, no comparison to be made. |
| --- | --- |

Mårtensson 1999

| **Reason for exclusion** | No comparison/control group |
| --- | --- |

Mårtensson 2001

| **Reason for exclusion** | Description of Doctorate |
| --- | --- |

Martín 2012

| **Reason for exclusion** | While the authors are using the FiQ as a measure of QoL in this study, its ability to measure this construct is not something that is supported by the original development paper. There are some effort to demonstrate construct validity between this measure and other dedicated HRQoL measures ([Rivera and González 2004](#REF-Rivera-and-Gonz_x00e1_lez-2004)), however correlations are broadly weak and the FiQ questions do not address the broad nature of HRQoL as a multidimensional construct.. |
| --- | --- |

Martin 2014

| **Reason for exclusion** | While the authors are using the FiQ as a measure of QoL in this study, its ability to measure this construct is not something that is supported by the original development paper. There are some effort to demonstrate construct validity between this measure and other dedicated HRQoL measures ([Rivera and González 2004](#REF-Rivera-and-Gonz_x00e1_lez-2004)), however correlations are broadly weak and the FiQ questions do not address the broad nature of HRQoL as a multidimensional construct. |
| --- | --- |

Martin 2014a

| **Reason for exclusion** | While the authors are using the FiQ as a measure of QoL in this study, its ability to measure this construct is not something that is supported by the original development paper. There are some effort to demonstrate construct validity between this measure and other dedicated HRQoL measures ([Rivera and González 2004](#REF-Rivera-and-Gonz_x00e1_lez-2004)), however correlations are broadly weak and the FiQ questions do not address the broad nature of HRQoL as a multidimensional construct. |
| --- | --- |

Martin 2014b

| **Reason for exclusion** | Using FiQ as a measure of HRQoL, this isn't valid. |
| --- | --- |

Martin 2017

| **Reason for exclusion** | No comparison/control group |
| --- | --- |

Martins et al 2022

| **Reason for exclusion** | no comparison group |
| --- | --- |

Mason 1998

| **Reason for exclusion** | While the authors are using the FiQ as a measure of QoL in this study, its ability to measure this construct is not something that is supported by the original development paper. There are some effort to demonstrate construct validity between this measure and other dedicated HRQoL measures ([Rivera and González 2004](#REF-Rivera-and-Gonz_x00e1_lez-2004)), however correlations are broadly weak and the FiQ questions do not address the broad nature of HRQoL as a multidimensional construct. |
| --- | --- |

McCormick 2015

| **Reason for exclusion** | No QoL |
| --- | --- |

McCoy 2016

| **Reason for exclusion** | PhD draft/preview rather than published research |
| --- | --- |

McCracken 2005

| **Reason for exclusion** | No QoL |
| --- | --- |

McCracken 2011

| **Reason for exclusion** | No QoL |
| --- | --- |

McGeary 2016

| **Reason for exclusion** | duplicate data from Gatchel 2009 |
| --- | --- |

McGillion 2008

| **Reason for exclusion** | Not delivered by multidisciplinary team |
| --- | --- |

Mehlsen 2015

| **Reason for exclusion** | No comparison/control group |
| --- | --- |

Mehlsen 2017

| **Reason for exclusion** | No QoL |
| --- | --- |

Meineche-Schmidt 2017

| **Reason for exclusion** | No comparison/control group |
| --- | --- |

Meng 2017

| **Reason for exclusion** | No QoL |
| --- | --- |

Merrick 2012

| **Reason for exclusion** | Authors have used the List11 scale. but then only used a single construct of ‘vocational health’. This is insufficient for inclusion in the present analysis and the original authors have not provided further detail. |
| --- | --- |

Milosavljevic 2015

| **Reason for exclusion** | Intervention delivery team not sufficiently broad |
| --- | --- |

Molander 2014

| **Reason for exclusion** | No comparison/control group |
| --- | --- |

Monticone 2008

| **Reason for exclusion** | No comparison/control group |
| --- | --- |

Monticone 2017

| **Reason for exclusion** | had been included originally, however was withdrawn by the publishers before completion of the project |
| --- | --- |

Monticone 2018

| **Reason for exclusion** | exp and comparison group conditions too similar |
| --- | --- |

Moradi 2010

| **Reason for exclusion** | No QoL |
| --- | --- |

Morales-Asencio 2016

| **Reason for exclusion** | Protocol |
| --- | --- |

Morlion 2011

| **Reason for exclusion** | No QoL |
| --- | --- |

Motoya 2017

| **Reason for exclusion** | No QoL |
| --- | --- |

Moulin 2015

| **Reason for exclusion** | No comparison/control group |
| --- | --- |

Nazzal 2013

| **Reason for exclusion** | No QoL |
| --- | --- |

Norén 2015

| **Reason for exclusion** | Poster presentation |
| --- | --- |

O'Sullivan 2015

| **Reason for exclusion** | No QoL |
| --- | --- |

Ólason 2018

| **Reason for exclusion** | No QoL |
| --- | --- |

Otten 2013

| **Reason for exclusion** | Review article |
| --- | --- |

Paolucci 2012

| **Reason for exclusion** | Duplication of Morone 2011 and also results are reported with respect to personality characteristics. |
| --- | --- |

Pearce 2007

| **Reason for exclusion** | Review article |
| --- | --- |

Perry 2010

| **Reason for exclusion** | The control group in this study had individual multidisciplinary support plans designed for them. These varied based on need with respect to duration, content and intensity. Due to the inability to differentiate between the intervention in the control and exp conditions it is not possible to include this study. |
| --- | --- |

Peters 2000

| **Reason for exclusion** | No comparison/control group |
| --- | --- |

Peterson 2011

| **Reason for exclusion** | Review article |
| --- | --- |

Peterson 2017

| **Reason for exclusion** | review article |
| --- | --- |

Pieber 2014

| **Reason for exclusion** | No comparison/control group |
| --- | --- |

Ris 2016

| **Reason for exclusion** |  |
| --- | --- |

Rivero-Arias 2005

| **Reason for exclusion** | Duplication of Fairbank 2005 |
| --- | --- |

Robinson 2001

| **Reason for exclusion** | No QoL |
| --- | --- |

Robinson 2004

| **Reason for exclusion** | It is a follow-up on Robinson 2001. in that original paper they didn't use a QoL measure, in this paper they have, however it is only taking a single post measurement so there is no pre-post comparison that can be made. |
| --- | --- |

Roche 2007

| **Reason for exclusion** | No QoL |
| --- | --- |

Rodevand 2017

| **Reason for exclusion** | No comparison/control group |
| --- | --- |

Rolving 2014

| **Reason for exclusion** | Protocol |
| --- | --- |

Rolving et al 2022

| **Reason for exclusion** | Sample is not simply chronic pain, but specifically people with chronic pain and a particular psychological profile. |
| --- | --- |

Rothman 2013

| **Reason for exclusion** | they are making comparisons of evaluation methods rather than treatment conditions. Participants are allocated to a treatment condition based on an evaluation by practitioners, then they are grouped back together at the end for analysis based on how they were evaluated rather than how they were treated. |
| --- | --- |

Ruth 2007

| **Reason for exclusion** | No QoL |
| --- | --- |

Rybarczyk 2001

| **Reason for exclusion** | Intervention not biopsychosocial |
| --- | --- |

Scascighini 2008

| **Reason for exclusion** | Review article |
| --- | --- |

Schmidt

| **Reason for exclusion** | Protocol and MSc project |
| --- | --- |

Schmidt 2018

| **Reason for exclusion** | No Results (justification and description paper) |
| --- | --- |

Schmidt 2021

| **Reason for exclusion** | The intensities of interventions were not suitably different to enable comparison within the remits of the current project. The keyway they vary is in the timing of delivery. |
| --- | --- |

Schramm 2014

| **Reason for exclusion** | No QoL |
| --- | --- |

Schuetze 2009

| **Reason for exclusion** | Not English language |
| --- | --- |

Schuetze 2014

| **Reason for exclusion** | No comparison/control group |
| --- | --- |

Scott 2015

| **Reason for exclusion** | Review article |
| --- | --- |

Scott 2017

| **Reason for exclusion** | No comparison/control group |
| --- | --- |

Seal 2020

| **Reason for exclusion** | Study became a tele-health approach due to covid pandemic, also, this source doesn't directly report results. |
| --- | --- |

Semrau 2015

| **Reason for exclusion** | Intervention conditions too similar |
| --- | --- |

Shaw 2014

| **Reason for exclusion** | No QoL |
| --- | --- |

Simopoulos 2018

| **Reason for exclusion** | Intervention not biopsychosocial |
| --- | --- |

Skouen 2002

| **Reason for exclusion** | No QoL |
| --- | --- |

Smeeding 2011

| **Reason for exclusion** | study comparing outcomes for different pain conditions rather than different types of pain intervention (no comparison/control) |
| --- | --- |

Sundelin 2013

| **Reason for exclusion** | Duplicate of Merrick 2013 |
| --- | --- |

Sørensen 2008

| **Reason for exclusion** | Intervention not Biopsychosocial |
| --- | --- |

Tait 1988

| **Reason for exclusion** | No QoL measure included |
| --- | --- |

Tavafian 2007

| **Reason for exclusion** | data duplicate for Tavafian 2008 |
| --- | --- |

Tavafian 2014

| **Reason for exclusion** | duplicated data with Tavafian 2017 |
| --- | --- |

Taylor 2016a

| **Reason for exclusion** | Duplicate of Taylor 2016 |
| --- | --- |

Tseli 2020

| **Reason for exclusion** | As all the interventions included are in the high category, it’s not possible for a comparison to be made within the parameters of the current review and the lack of a control group means the source will have to be excluded. |
| --- | --- |

Turcotte et al 2023

| **Reason for exclusion** | no QoL measure |
| --- | --- |

Turner-Stokes 2003

| **Reason for exclusion** | No QoL |
| --- | --- |

Uhlemann 2007

| **Reason for exclusion** | Intervention not Biopsychosocial |
| --- | --- |

Vachon-Presseau 2013

| **Reason for exclusion** | No QoL |
| --- | --- |

van Amstel 2018

| **Reason for exclusion** | Intervention team was not interdisciplinary. |
| --- | --- |

van der Maas 2016

| **Reason for exclusion** | Data duplicate from Van Der Mass 2015 |
| --- | --- |

Van der Mass (chapter 6)

| **Reason for exclusion** | Duplicate of Van Der Mass 2015 |
| --- | --- |

van der Zee 2011

| **Reason for exclusion** | No QoL |
| --- | --- |

van Geen 2007

| **Reason for exclusion** | Review article |
| --- | --- |

van Hooff 2011

| **Reason for exclusion** | Poster session summary |
| --- | --- |

van Wijk 2008

| **Reason for exclusion** | Intervention not Biopsychosocial |
| --- | --- |

Vincent 2013

| **Reason for exclusion** | Review article |
| --- | --- |

Vlaeyen 1996

| **Reason for exclusion** | No QoL |
| --- | --- |

Volker 2017

| **Reason for exclusion** | No comparison/control group |
| --- | --- |

Vollenbroek-Hutten 2004

| **Reason for exclusion** | Same sample as van der Hulst 2008a therefore excluded to avoid duplication. Other study chosen over this earlier publication due to desire for greater homogeneity of outcome measures and its use of SF-36 instead of the EQ-5D (as was the case in this 2004 study). |
| --- | --- |

Vong 2011

| **Reason for exclusion** | intervention delivered by a single professional rather than multidisciplinary team. |
| --- | --- |

Vriezekolk 2013

| **Reason for exclusion** | No comparison/control group |
| --- | --- |

Waheed 2013

| **Reason for exclusion** | Intervention not Biopsychosocial |
| --- | --- |

Walker 1998

| **Reason for exclusion** | Commentary |
| --- | --- |

Wallasch 2012

| **Reason for exclusion** | No comparison/control group |
| --- | --- |

Wetherell 2011

| **Reason for exclusion** | Intervention not biopsychosocial |
| --- | --- |

Wicksell 2013

| **Reason for exclusion** | Intervention not Biopsychosocial |
| --- | --- |

Wiesender 2006

| **Reason for exclusion** | Review article |
| --- | --- |

Wilson 2018

| **Reason for exclusion** | Review article |
| --- | --- |

Wong 2009

| **Reason for exclusion** | No comparison/control group |
| --- | --- |

Wong 2011

| **Reason for exclusion** | intervention only delivered by single practitioner |
| --- | --- |

Yea-Ing 2013

| **Reason for exclusion** | Not Chronic Pain patients |
| --- | --- |

# AppendixS9: Overview of all measures and subscales included in the analyses.

**SF36**

PCS

MCS

Physical functioning

Role Physical

pain

general health

vitality

social functioning

role emotional

emotional wellbeing

**SF12**

PCS

MCS

Vitality

Physical functioning

Bodily pain

General health perceptions

Physical role functioning

Emotional role functioning

Social role functioning

Mental health

**Euro QoL**

**EQ15D**

**EQ5D**

**Dallas Pain questionnaire**

ADL

Work/Leisure

Anxiety/Depression

Social Interest

**Nottingham health profile**

**Part 1**

energy

Pain

emotional reactions

social isolation

sleep disruption

physical mobility

Total score

**The quality-of-life scale**

material and physical well-being

relationships with other people

social, Community and civic activities

personal development and fulfilment

recreation

**lisat-9**

Life as a whole

Self-care

Vocational situation

Financial situation

Leisure situation

Sexual life

Partner relations

Family Life

Contact w/ friends

**Icelandic Quality of Life (IQL) scale**

general health

depression

social functioning

financial status

energy

anxiety

physical health

self-control

sleep

general wellbeing

# AppendixS10: Supporting passage for the reading of table 7

As a brief supporting guide to interpreting table 3 consider the group of outcomes for PFWB, IMPT vs TAU, at 0-3 months (this is the first result shown). The first row is the main analysis. Findings show a small positive effect (SMD = 0.41) in favour of the intervention - CIs (i.e. 0.20; 0.62) do not cross 0, but heterogeneity levels are violated (i.e. I^2^>60). The second row presents the findings from analysis of higher quality sources only and again shows a small positive effect in favour of the intervention (SMD = 0.46) - CIs do not cross 0, and, this time, heterogeneity is acceptable. In the third row we see the findings from analyses excluding outliers; a small positive effect in favour of the intervention is shown (SMD = 0.33) - CIs do not cross 0 and acceptable heterogeneity. Taken in sum, these outcomes provide consistent support for the intervention. Thus, the rating of ‘maybe’ for Clinical relevance (see appendix 6 for a full overview of the process for allocating clinical relevance categories).

In cases where trim and fill analysis indicated possible asymmetry in the sample, this is also reported in an additional fourth row (e.g., see results for Physical Functioning and Wellbeing, IMPT vs. TAU, at 4-11 months). Any adjusted models from these analyses are reported in table 3. However, as outlined in appendix 6 and in line with guidance (Page et al., 2019), they only contributed to assessments of clinical relevance when the sample of studies was 10 or larger. Egger test results are also reported for trim and fill analysis (Sterne & Egger, 2005) in cases where p<0.05 asymmetry could be attributed to potential publication bias (e.g. physical functioning and wellbeing IMPT vs. TAU Intermediate term follow up). Finally, subgroup analysis comparing different intervention intensities was applied for each outcome. However, as no significant differences were found for any subgroup analyses these results are not reported here (Risk of bias evaluations, forest plots and data for individual studies included in all analyses can be found within the supplementary digital content).

# AppendixS11: Example of subgroup analysis by intervention duration


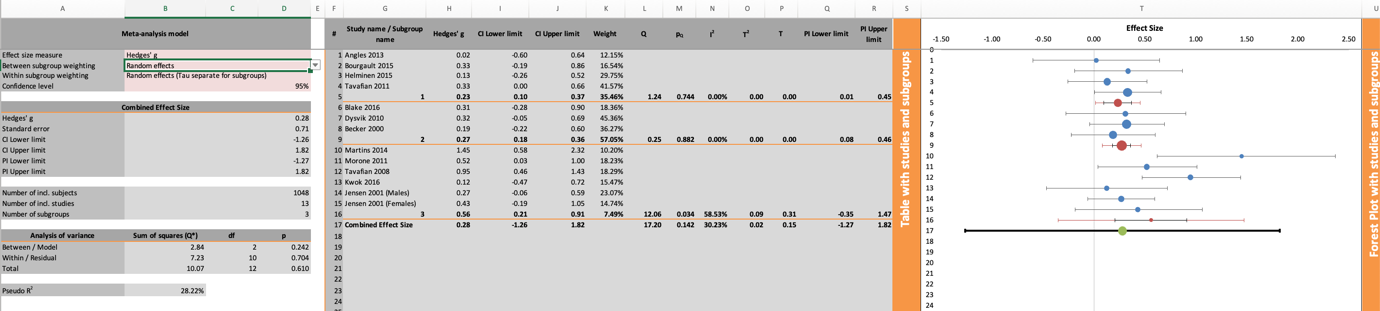


# AppendixS12: Description and sample for potential/trailed narrowing of analyses

The authors considered whether restricting certain aspects of the study might provide greater clarity on the size or nature of effects. They explored whether limiting the analysis to a specific pain condition and a single validated measure could offer improved clinical insight and applicability.

To test this, an additional analysis was conducted. To maximise the available sample size, the authors focused on the most common pain condition in the dataset (non - specific chronic low back pain) and the most frequently used HRQoL measure (SF - 36). This approach identified 12 relevant studies, which were then grouped based on comparisons with TAU (n=5) or ACG (n=7). The larger of these two groups was selected for comparative analysis and further separated by Follow-up time points (short-term: n=6, intermediate: n=1, long-term: n=5). The short-term group had a larger total participant count and was chosen for final analysis, with emotional functioning and wellbeing being selected as the domain for analysis.

To illustrate the impact of these restrictions on the analysis: in the full sample of 10 studies (SMD = 0.36; 95% CI (0.02; 0.87); p = 0.02; I^2^ = 77%); in the restricted sample of five studies (SMD = 0.34; 95% CI (-0.60; 1.27); p = 0.319; I^2^ = 84.51%). These findings indicate that, despite similarity in the reported effect sizes, restricting the analysis to a single pain condition and measurement tool did not reduce heterogeneity (I^2^) or narrow the confidence intervals. Accordingly, further analyses based on this restrictive approach were not conducted.

| **MBI vs ACG - Non-specific chronic low back pain measued by SF-36** | | |  |  |  |  |  |
| --- | --- | --- | --- | --- | --- | --- | --- |
| A - SML | Dufour 2010 | Non-specific chronic low back pain | n=286 | 56% female | Medium duration IMPT vs Low duration ACG | Denmark | SF36 - PCS, MCS, PF, BP, GH, RE, V MH, SF, RP |
| A-SL | Monticone 2012 | Non-specific chronic low back pain | n=80 | 75% female | Low Duration IMPT vs. Low duration ACG | Italy | SF36 - PF, BP, GH, RE, V MH, SF, RP |
| A-SL | Monticone 2013 | Non-specific chronic low back pain | n=90 | 58% female | Low Duration IMPT vs. Low duration ACG | Italy | SF36 - PF, BP, GH, RE, V MH, SF, RP |
| A-SM | Monticone 2014 | Non-specific chronic low back pain | n=10 | 55% female | Low Duration IMPT vs. Low duration ACG | Italy | SF36 - PF, BP, GH, RE, V MH, SF, RP |
| A-S | Paolucci 2017 | Non-specific chronic low back pain | n=53 | 82% female | Low Duration IMPT vs. Low duration ACG | Italy | SF36 - PF, BP, GH, RE, V MH, SF, RP |
| P-SML | Tavafian 2008 | Non-specific chronic low back pain | n=102 | 100% female | Low Duration IMPT vs. Low duration ACG | Iran | SF36 - PCS, MCS |
| A-l | Ronzi 2017 | Non-specific chronic low back pain | n=159 | 40% female | High duration IMPT vs Med duration IMPT vs. Low duration ACG | France | SF36 - PCS, MCS |
| **MBI vs TAU - Non-specific chronic low back pain measued by SF-36** | | |  |  |  |  |  |
| P-SM | Van der Hulst 2008 | Non-specific chronic low back pain | n=163 | 39% female | Medium duration IMPT vs TAU | Holland | SF36 - PCS, MCS |
| p-M | Lang 2003 | Non-specific chronic low back pain | n=208 | 58% female | High duration IMPT vs TAU | Germany | SF36 - PCS, MCS, PF, BP, GH, RE, V MH, SF, RP |
| P-SM | Tavafian 2011 | Non-specific chronic low back pain | n=197 | 78% female | Low Duration IMPT vs. TAU | Iran | SF36 - PF, BP, GH, RE, V MH, SF, RP |
| P-L | Tavafian 2017 | Non-specific chronic low back pain | n=197 | 78% female | Low Duration IMPT vs. TAU | Iran | SF36 - PF, BP, GH, RE, V MH, SF, RP |
| A-l | Jensen 2011 | Non-specific chronic low back pain | n=351 | 52% female | Unclear duration IMPT vs TAU | Denmark | SF36 - PF, BP, GH, RE, V MH, SF, RP |
