# Supplementary material for: Assessing the Impact of Interdisciplinary Multimodal Pain Treatment on Health‐Related Quality of Life in Chronic Pain: A Systematic Review and Meta‐Analyses
Source: Eur J Pain. 2025 Dec 2;30(1):e70176. doi: 10.1002/ejp.70176 (PMC12670346; doi:10.1002/ejp.70176)

# Data, Forest Plots and ROB for all outcomes

## Physical Functioning and Wellbeing - IMPT VS TAU

### Short term follow-up

| Study | Hedges' g | CI Lower limit | CI Upper limit | Weight |
| --- | --- | --- | --- | --- |
| Angeles 2013 | -0.28 | -0.91 | 0.34 | 5.27% |
| Blake 2016 | 0.26 | -0.32 | 0.85 | 5.59% |
| Bourgault 2015 | -0.05 | -0.57 | 0.48 | 6.21% |
| Becker 2000 | 0.28 | -0.14 | 0.69 | 7.44% |
| Dysvik 2010 | 0.05 | -0.31 | 0.42 | 8.02% |
| Helminen 2015 | 0.25 | -0.14 | 0.64 | 7.73% |
| Kwok 2016 | 0.89 | 0.29 | 1.53 | 5.27% |
| Martins 2014 | 0.90 | 0.11 | 1.74 | 3.93% |
| Morone 2011 | 0.25 | -0.23 | 0.73 | 6.66% |
| Tavafian 2008 | 1.13 | 0.64 | 1.63 | 6.50% |
| Van der Hulst 2008 | 0.43 | 0.11 | 0.75 | 8.60% |
| Tavafian 2011 | 0.50 | 0.17 | 0.83 | 8.47% |
| Jensen 2001 (male) | 0.38 | -0.24 | 1.01 | 5.27% |
| Jensen 2001 (female) | 0.27 | -0.24 | 0.80 | 6.23% |
| Björnsdóttir 2016 | 0.92 | 0.62 | 1.22 | 8.81% |


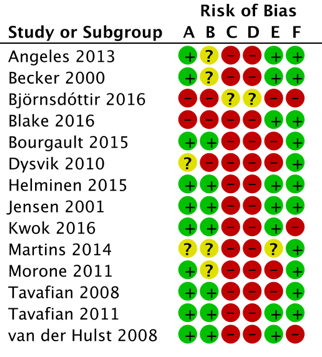


### Intermediate term follow-up

| Study | Hedges' g | CI Lower limit | CI Upper limit | Weight |
| --- | --- | --- | --- | --- |
| Amris 2014 | -0.01 | -0.29 | 0.28 | 10.54% |
| Saral 2016 | 1.12 | 0.46 | 1.82 | 4.81% |
| Tavafian 2008 | 0.65 | 0.18 | 1.12 | 7.24% |
| Van der Hulst 2008 | 0.42 | 0.11 | 0.75 | 9.84% |
| Becker 2000 | 0.42 | 0.01 | 0.84 | 8.10% |
| Cedraschi 2004 | 0.16 | -0.14 | 0.47 | 10.10% |
| Lang 2003 | 0.25 | -0.07 | 0.57 | 9.89% |
| Morone 2011 | 0.56 | 0.08 | 1.06 | 6.98% |
| Tavafian 2011 | 0.77 | 0.44 | 1.12 | 9.50% |
| Jensen 2001 (male) | 0.00 | -0.62 | 0.61 | 5.39% |
| Jensen 2001 (female) | 0.43 | -0.09 | 0.96 | 6.48% |
| Grahn 1998 | 0.04 | -0.21 | 0.30 | 11.13% |


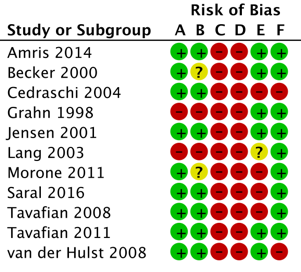


### Long term follow-up

| Study | Hedges' g | CI Lower limit | CI Upper limit | Weight |
| --- | --- | --- | --- | --- |
| Helminen 2015; | 0.16 | -0.23 | 0.55 | 17.54% |
| Tavafian 2008 | 0.56 | 0.10 | 1.04 | 15.98% |
| Tavafian 2017 | 0.65 | 0.32 | 0.99 | 18.63% |
| Grahn 2000; | -0.08 | -0.32 | 0.16 | 20.37% |
| Jensen 2001 (male) | 0.48 | -0.14 | 1.11 | 13.07% |
| Jensen 2001 (female) | 0.96 | 0.42 | 1.52 | 14.42% |


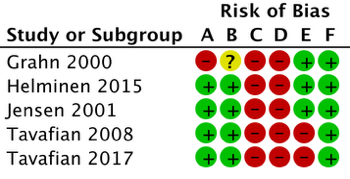


## Physical Functioning and Wellbeing - IMPT VS ACG

### Short term follow-up

| Study | Hedges' g | CI Lower limit | CI Upper limit | Weight |
| --- | --- | --- | --- | --- |
| Angst 2009 | 0.01 | -0.21 | 0.23 | 11.60% |
| Dufour 2010 | 0.23 | -0.01 | 0.47 | 11.48% |
| Gatchel 2009 | 1.32 | 0.79 | 1.87 | 8.44% |
| Jensen 2001 Males | 0.11 | -0.48 | 0.71 | 7.95% |
| Jensen 2001 Females | 0.27 | -0.21 | 0.76 | 8.99% |
| Monticone 2012 | -0.51 | -0.97 | -0.07 | 9.39% |
| Monticone 2014 | 1.70 | 0.72 | 2.84 | 4.61% |
| Paolucci 2017 | 0.03 | -0.51 | 0.58 | 8.43% |
| Becker 2000 | 0.31 | -0.10 | 0.73 | 9.72% |
| Van Der Maas 2015 | 0.16 | -0.25 | 0.57 | 9.82% |
| Monticone 2013 | 0.76 | 0.33 | 1.19 | 9.58% |


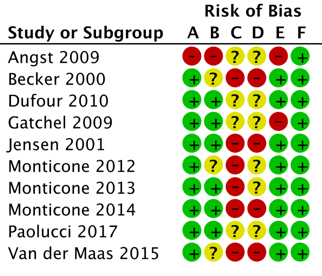


### Intermediate term follow-up

| Study | Hedges' g | CI Lower limit | CI Upper limit | Weight |
| --- | --- | --- | --- | --- |
| Angst 2009 | -0.18 | -0.46 | 0.09 | 12.72% |
| Dufour 2010 | 0.23 | -0.01 | 0.47 | 13.11% |
| Gatchel 2009 | 1.20 | 0.68 | 1.74 | 9.35% |
| Lera 2009 | 0.12 | -0.37 | 0.61 | 9.91% |
| Van Der Maas 2015 | 0.32 | -0.09 | 0.73 | 10.93% |
| Jensen 2001 Males | -0.02 | -0.61 | 0.57 | 8.65% |
| Jensen 2001 Females | 0.29 | -0.19 | 0.78 | 9.91% |
| Monticone 2014 | 1.96 | 0.93 | 3.15 | 4.51% |
| Becker 2000 | 0.36 | -0.06 | 0.78 | 10.82% |
| Rooks 2007 | 0.08 | -0.40 | 0.55 | 10.11% |


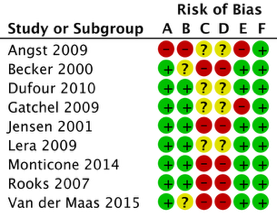


### Long term follow up

| Study | Hedges' g | CI Lower limit | CI Upper limit | Weight |
| --- | --- | --- | --- | --- |
| Dufour 2010 | 0.38 | 0.14 | 0.62 | 12.99% |
| Gatchel 2009 | 1.20 | 0.68 | 1.74 | 9.30% |
| Ronzi 2017 | 0.33 | -0.05 | 0.73 | 11.09% |
| Van Der Maas 2015 | 0.28 | -0.13 | 0.69 | 10.86% |
| Westman 2010 | 0.16 | -1.14 | 1.48 | 3.68% |
| Jensen 2001 Males | 0.51 | -0.08 | 1.12 | 8.50% |
| Jensen 2001 Females | -0.13 | -0.62 | 0.35 | 9.88% |
| Monticone 2012 | 0.06 | -0.38 | 0.51 | 10.45% |
| Monticone 2013 | 0.86 | 0.44 | 1.31 | 10.52% |
| Jensen 2011 | -0.19 | -0.45 | 0.08 | 12.72% |


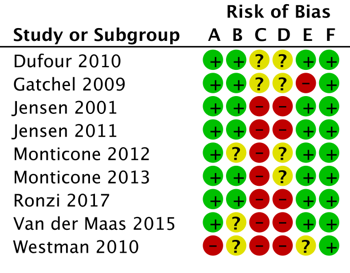


## Emotional Functioning and Wellbeing vs TAU

### Short term follow-up

| Study | Hedges' g | CI Lower limit | CI Upper limit | Weight |
| --- | --- | --- | --- | --- |
| Angles 2013 | 0.02 | -0.60 | 0.64 | 5.21% |
| Blake 2016 | 0.31 | -0.27 | 0.90 | 5.65% |
| Bourgault 2015 | 0.33 | -0.19 | 0.87 | 6.62% |
| Dysvik 2010 | 0.32 | -0.04 | 0.69 | 10.62% |
| Helminen 2015 | 0.13 | -0.26 | 0.52 | 9.92% |
| Martins 2014 | 1.45 | 0.62 | 2.37 | 3.01% |
| Morone 2011 | 0.52 | 0.04 | 1.01 | 7.40% |
| Tavafian 2008 | 0.95 | 0.47 | 1.44 | 7.45% |
| Kwok 2016 | 0.12 | -0.47 | 0.72 | 5.57% |
| Becker 2000 | 0.19 | -0.22 | 0.60 | 9.23% |
| Tavafian 2011 | 0.33 | 0.00 | 0.66 | 12.07% |
| Jensen 2001 (Males) | 0.27 | -0.06 | 0.59 | 12.10% |
| Jensen 2001 (Females) | 0.43 | -0.19 | 1.06 | 5.15% |


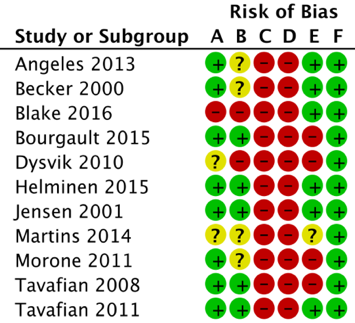


### Intermediate term follow-up

| Study | Hedges' g | CI Lower limit | CI Upper limit | Weight |
| --- | --- | --- | --- | --- |
| Amris 2014 | 0.00 | -0.28 | 0.29 | 15.59% |
| Lang 2003 | 0.29 | -0.20 | 0.78 | 5.79% |
| Morone 2011 | 0.37 | -0.11 | 0.86 | 5.93% |
| Saral 2016 | 0.79 | 0.33 | 1.26 | 6.22% |
| Tavafian 2008 | 0.41 | -0.05 | 0.88 | 6.40% |
| Van der Hulst 2008 | 0.40 | 0.08 | 0.72 | 12.59% |
| Tavafian 2011 | 0.20 | -0.13 | 0.53 | 12.16% |
| Jensen 2001 (Males) | 0.42 | -0.20 | 1.05 | 3.69% |
| Jensen 2001 (Females) | 0.42 | -0.10 | 0.95 | 5.08% |
| Grahn 1998 | 0.19 | -0.06 | 0.45 | 18.68% |
| Becker 2000 | 0.33 | -0.08 | 0.75 | 7.87% |


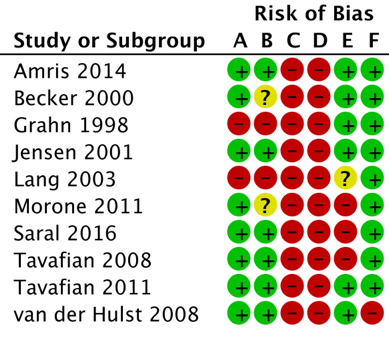


### Long term follow-up

| Study | Hedges' g | CI Lower limit | CI Upper limit | Weight |
| --- | --- | --- | --- | --- |
| Helminen 2015 | 0.06 | -0.33 | 0.45 | 17.77% |
| Tavafian 2008 | 0.26 | -0.20 | 0.72 | 15.27% |
| Tavafian 2017 | 0.67 | 0.34 | 1.01 | 19.89% |
| Jensen 2001 (Males) | 0.63 | 0.01 | 1.27 | 10.74% |
| Jensen 2001 (Females) | 0.70 | 0.18 | 1.25 | 13.04% |
| Grahn 2000 | 0.12 | -0.14 | 0.38 | 23.30% |


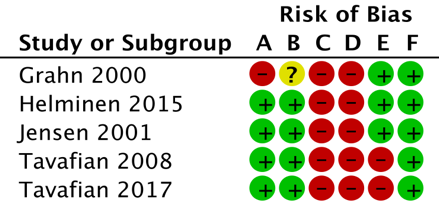


## Emotional Functioning and Wellbeing vs ACGs

### Short term follow-up

| Study | Hedges' g | CI Lower limit | CI Upper limit | Weight |
| --- | --- | --- | --- | --- |
| Angst 2009 | 0.17 | -0.06 | 0.39 | 11.63% |
| Dufour 2010 | 0.15 | -0.09 | 0.39 | 11.51% |
| Gatchel 2009 | -0.04 | -0.53 | 0.44 | 8.93% |
| Van Der Maas 2015 | 0.18 | -0.23 | 0.59 | 9.79% |
| Jensen 2001 (males) | 0.16 | -0.43 | 0.76 | 7.88% |
| Jensen 2001 (females) | 0.63 | 0.14 | 1.13 | 8.83% |
| Monticone 2012 | -0.26 | -0.71 | 0.18 | 9.41% |
| Monticone 2014 | 1.04 | 0.12 | 2.05 | 5.10% |
| Paolucci 2017 | 0.89 | 0.33 | 1.47 | 8.08% |
| Becker 2000 | 0.11 | -0.30 | 0.53 | 9.71% |
| Monticone 2013 | 1.45 | 0.99 | 1.92 | 9.13% |


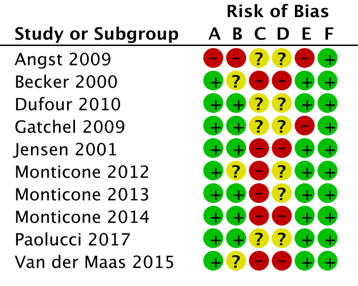


## Intermediate term follow-up

| Study | Hedges' g | CI Lower limit | CI Upper limit | Weight |
| --- | --- | --- | --- | --- |
| Angst 2009 | -0.07 | -0.34 | 0.20 | 17.70% |
| Dufour 2010 | 0.06 | -0.18 | 0.29 | 20.19% |
| Gatchel 2009 | 0.35 | -0.14 | 0.85 | 8.03% |
| Lera 2009 | 0.23 | -0.25 | 0.72 | 8.11% |
| Van Der Maas 2015 | 0.02 | -0.39 | 0.43 | 10.63% |
| Jensen 2001 (males) | 0.14 | -0.45 | 0.74 | 5.99% |
| Jensen 2001 (females) | 0.26 | -0.22 | 0.75 | 8.15% |
| Monticone 2014 | 1.29 | 0.35 | 2.34 | 2.54% |
| Becker 2000 | 0.37 | -0.04 | 0.79 | 10.22% |
| Rooks 2007 | 0.37 | -0.10 | 0.85 | 8.44% |

**
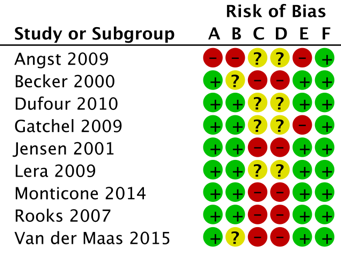
**

### Long term follow up

| Study | Hedges' g | CI Lower limit | CI Upper limit | Weight |
| --- | --- | --- | --- | --- |
| Dufour 2010 | 0.17 | -0.07 | 0.41 | 12.63% |
| Jensen 2001 (males) | 0.06 | -0.53 | 0.65 | 9.41% |
| Jensen 2001 (Females) | 0.16 | -0.32 | 0.65 | 10.42% |
| Monticone 2012 | -0.06 | -0.50 | 0.38 | 10.85% |
| Monticone 2013 | 1.98 | 1.49 | 2.51 | 10.17% |
| Ronzi 2017 | 0.06 | -0.33 | 0.45 | 11.35% |
| Westman 2010 | 0.18 | -0.20 | 0.55 | 11.47% |
| Jensen 2011 | 0.27 | 0.01 | 0.52 | 12.52% |
| Van Der Maas 2015 | -0.10 | -0.51 | 0.31 | 11.18% |


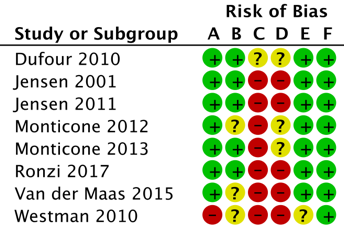


## General Health vs TAU

### Short term follow-up

| Study | Hedges' g | CI Lower limit | CI Upper limit | Weight |
| --- | --- | --- | --- | --- |
| Angles 2013 | -0.09 | -0.72 | 0.53 | 8.07% |
| Becker 2000; | 0.48 | 0.07 | 0.90 | 11.83% |
| Dysvik 2010; | 0.61 | 0.25 | 0.99 | 12.89% |
| Helminen 2015; | 0.06 | -0.33 | 0.45 | 12.50% |
| Kwok 2016 | 0.66 | 0.07 | 1.29 | 8.23% |
| Tavafian 2011 | 0.37 | 0.04 | 0.70 | 13.98% |
| Jensen 2001 (male) | 0.08 | -0.53 | 0.70 | 8.10% |
| Jensen 2001 (female) | -0.01 | -0.53 | 0.51 | 9.75% |
| Björnsdóttir 2016; | 0.82 | 0.53 | 1.13 | 14.66% |


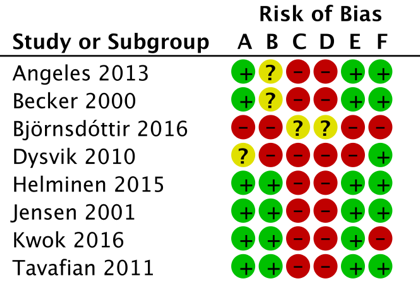


### Intermediate term follow-up

| Study | Hedges' g | CI Lower limit | CI Upper limit | Weight |
| --- | --- | --- | --- | --- |
| Becker 2000; | 0.67 | 0.26 | 1.10 | 14.34% |
| Cedraschi 2004 | 0.22 | -0.08 | 0.53 | 23.77% |
| Lang 2003 | 0.09 | -0.22 | 0.41 | 22.72% |
| Tavafian 2011 | 0.29 | -0.04 | 0.61 | 21.64% |
| Jensen 2001 (male) | 0.42 | -0.19 | 1.05 | 7.37% |
| Jensen 2001 (female) | 0.03 | -0.49 | 0.55 | 10.17% |


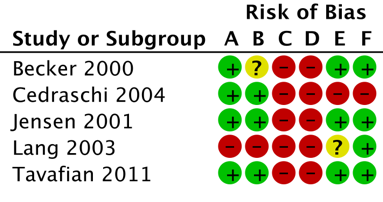


### Long term follow-up

| Study | Hedges' g | CI Lower limit | CI Upper limit | Weight |
| --- | --- | --- | --- | --- |
| Tavafian 2017 | 0.42 | 0.10 | 0.76 | 59.09% |
| Jensen 2001 (male) | 0.64 | 0.02 | 1.28 | 16.76% |
| Jensen 2001 (female) | 0.32 | -0.20 | 0.84 | 24.14% |


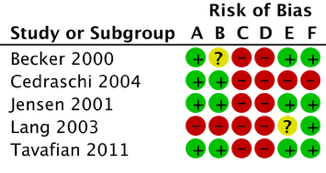


## General Health vs ACGs

### Short term follow-up

| Study name | Hedges' g | CI Lower limit | CI Upper limit | Weight |
| --- | --- | --- | --- | --- |
| Angst 2009 | -0.03 | -0.25 | 0.20 | 12.97% |
| Dufour 2010 | -0.07 | -0.31 | 0.17 | 12.90% |
| Jensen 2001 (males) | 0.02 | -0.57 | 0.61 | 10.36% |
| Jensen 2001 (females) | 0.20 | -0.28 | 0.69 | 11.19% |
| Monticone 2012 | -0.21 | -0.65 | 0.23 | 11.53% |
| Monticone 2014 | 1.22 | 0.28 | 2.26 | 7.57% |
| Paolucci 2017 | -0.01 | -0.56 | 0.53 | 10.74% |
| Becker 2000 | 0.20 | -0.22 | 0.61 | 11.73% |
| Monticone 2013 | 1.94 | 1.46 | 2.47 | 11.01% |


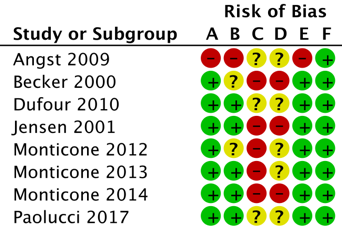


### Intermediate term follow-up

| Study name | Hedges' g | CI Lower limit | CI Upper limit | Weight |
| --- | --- | --- | --- | --- |
| Angst 2009 | 0.10 | -0.17 | 0.37 | 20.76% |
| Dufour 2010 | -0.07 | -0.31 | 0.17 | 22.14% |
| Jensen 2001 (males) | 0.32 | -0.27 | 0.92 | 10.41% |
| Jensen 2001 (females) | -0.05 | -0.54 | 0.43 | 13.12% |
| Monticone 2014 | 1.42 | 0.47 | 2.50 | 5.03% |
| Becker 2000 | 0.37 | -0.04 | 0.80 | 15.18% |
| Rooks 2007 | 0.40 | -0.08 | 0.88 | 13.36% |


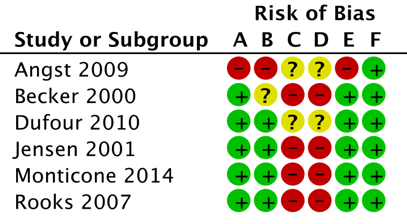


### Long term follow-up

| Study name | Hedges' g | CI Lower limit | CI Upper limit | Weight |
| --- | --- | --- | --- | --- |
| Dufour 2010 | 0.13 | -0.10 | 0.37 | 18.50% |
| Jensen 2001 (males) | 0.09 | -0.50 | 0.68 | 14.81% |
| Jensen 2001 (Females) | 0.27 | -0.22 | 0.76 | 16.01% |
| Monticone 2012 | 0.08 | -0.36 | 0.52 | 16.52% |
| Monticone 2013 | 1.92 | 1.43 | 2.44 | 15.79% |
| Jensen 2011 | 0.24 | -0.01 | 0.49 | 18.37% |


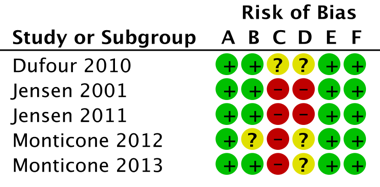


## Overall HRQoL VS TAU

### Short term follow-up

| Study name | Hedges' g | CI Lower limit | CI Upper limit | Weight |
| --- | --- | --- | --- | --- |
| Björnsdóttir 2016 | 1.31 | 1.00 | 1.62 | 32.82% |
| Hurley 2007 | 0.01 | -0.22 | 0.24 | 33.82% |
| Luciano 2013 | 0.35 | 0.08 | 0.62 | 33.36% |

###
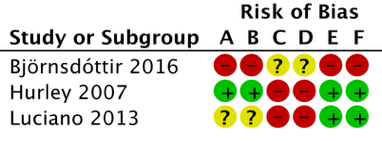


### Intermediate term follow-up

| Study name | Hedges' g | CI Lower limit | CI Upper limit | Weight |
| --- | --- | --- | --- | --- |
| Grahn 1998 | 0.16 | -0.10 | 0.41 | 20.70% |
| Taylor 2016; | 0.09 | -0.06 | 0.24 | 60.29% |
| Luciano 2013 | 0.11 | -0.16 | 0.38 | 19.01% |


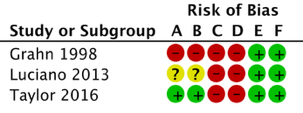


### Long term follow-up

| Study name | Hedges' g | CI Lower limit | CI Upper limit | Weight |
| --- | --- | --- | --- | --- |
| Grahn 2000; | 0.15 | -0.10 | 0.41 | 27.04% |
| Taylor 2016; | -0.03 | -0.18 | 0.12 | 32.80% |
| Luciano 2013 | 0.48 | 0.21 | 0.76 | 26.20% |
| Nygaard 2020 | -0.06 | -0.61 | 0.50 | 13.97% |

##
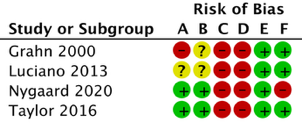


## Pain VS TAU

### Short term follow-up

| Study name | Hedges' g | CI Lower limit | CI Upper limit | Weight |  |
| --- | --- | --- | --- | --- | --- |
| Angles 2013 | 0.96 | 0.32 | 1.64 | 6.46% |  |
| Becker 2000 | 0.48 | 0.07 | 0.91 | 12.61% |  |
| Dysvik 2010 | 0.25 | -0.11 | 0.62 | 14.90% |  |
| Helminen 2015 | 0.09 | -0.30 | 0.48 | 13.85% |  |
| Tavafian 2011 | 0.45 | 0.12 | 0.78 | 16.90% |  |
| Jensen 2001 (male) | 0.01 | -0.61 | 0.62 | 7.12% |  |
| Jensen 2001 (female) | 0.21 | -0.31 | 0.73 | 9.26% |  |
| Björnsdóttir 2016 | 0.60 | 0.30 | 0.90 | 18.90% |  |

###
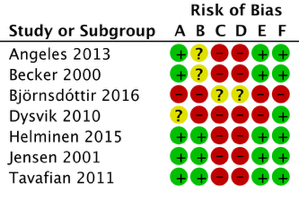


### Intermediate term follow-up

| Study name | Hedges' g | CI Lower limit | CI Upper limit | Weight |
| --- | --- | --- | --- | --- |
| Becker 2000; | 0.71 | 0.29 | 1.14 | 16.23% |
| Lang 2003 | 0.47 | 0.15 | 0.79 | 19.11% |
| Tavafian 2011 | 0.47 | 0.14 | 0.80 | 18.82% |
| Jensen 2001 (male) | 0.07 | -0.54 | 0.69 | 11.80% |
| Jensen 2001 (female) | 1.01 | 0.47 | 1.58 | 13.12% |
| Grahn 1998 | 0.03 | -0.23 | 0.28 | 20.92% |


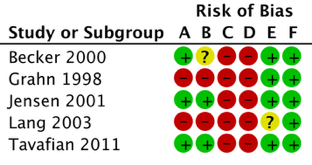


### Long term follow-up

| Study name | Hedges' g | CI Lower limit | CI Upper limit | Weight |
| --- | --- | --- | --- | --- |
| Tavafian 2017 | 0.56 | 0.23 | 0.90 | 27.85% |
| Jensen 2001 (male) | 0.65 | 0.03 | 1.29 | 20.34% |
| Jensen 2001 (female) | 1.05 | 0.51 | 1.62 | 22.18% |
| Grahn 2000; | 0.03 | -0.23 | 0.28 | 29.63% |


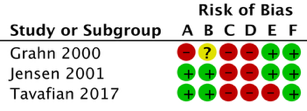


## Pain VS ACGs

### Short term follow-up

| Study name | Hedges' g | CI Lower limit | CI Upper limit | Weight |
| --- | --- | --- | --- | --- |
| Angst 2009 | -0.19 | -0.41 | 0.04 | 13.33% |
| Dufour 2010 | 0.27 | 0.03 | 0.51 | 13.23% |
| Jensen 2001 (males) | -0.06 | -0.65 | 0.53 | 10.10% |
| Jensen 2001 (females) | 0.38 | -0.10 | 0.87 | 11.06% |
| Monticone 2012 | -0.08 | -0.52 | 0.37 | 11.52% |
| Monticone 2014 | 0.81 | -0.09 | 1.79 | 7.34% |
| Paolucci 2017 | 0.00 | -0.55 | 0.54 | 10.55% |
| Becker 2000 | 0.29 | -0.13 | 0.70 | 11.75% |
| Monticone 2013 | 1.62 | 1.15 | 2.11 | 11.13% |


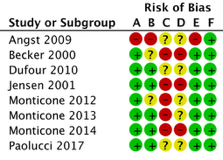


### Intermediate term follow-up

| Study name | Hedges' g | CI Lower limit | CI Upper limit | Weight |
| --- | --- | --- | --- | --- |
| Angst 2009; | -0.25 | -0.52 | 0.02 | 20.42% |
| Dufour 2010 | 0.16 | -0.08 | 0.40 | 21.73% |
| Jensen 2001 (males) | 0.42 | -0.17 | 1.03 | 10.43% |
| Jensen 2001 (females) | 0.46 | -0.03 | 0.96 | 12.97% |
| Monticone 2014 | 0.88 | -0.02 | 1.87 | 5.76% |
| Becker 2000; | 0.31 | -0.10 | 0.73 | 15.18% |
| Rooks 2007 | 0.08 | -0.39 | 0.55 | 13.52% |

###
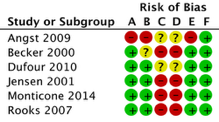


### Long term follow-up

| Study name | Hedges' g | CI Lower limit | CI Upper limit | Weight |
| --- | --- | --- | --- | --- |
| Dufour 2010 | 0.22 | -0.02 | 0.46 | 18.11% |
| Jensen 2001 (males) | 0.41 | -0.18 | 1.02 | 15.10% |
| Jensen 2001 (Females) | 0.35 | -0.13 | 0.84 | 16.13% |
| Monticone 2012 | -0.33 | -0.77 | 0.11 | 16.54% |
| Monticone 2013 | 1.77 | 1.29 | 2.28 | 16.08% |
| Jensen 2011 | -0.19 | -0.44 | 0.07 | 18.03% |

##
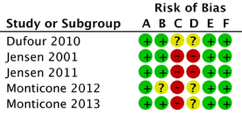


## Social Functioning VS TAU

### Short term follow-up

| Study name | Hedges' g | CI Lower limit | CI Upper limit | Weight |
| --- | --- | --- | --- | --- |
| Angles 2013 | 0.02 | -0.60 | 0.64 | 7.89% |
| Becker 2000; | 0.07 | -0.34 | 0.48 | 11.96% |
| Dysvik 2010; | 0.17 | -0.20 | 0.53 | 13.10% |
| Helminen 2015; | 0.12 | -0.27 | 0.51 | 12.51% |
| Tavafian 2011 | 0.22 | -0.10 | 0.55 | 14.17% |
| Jensen 2001 (male) | -0.03 | -0.64 | 0.59 | 7.92% |
| Jensen 2001 (female) | 0.03 | -0.49 | 0.55 | 9.62% |
| Kwok 2016 | 0.81 | 0.21 | 1.45 | 7.92% |
| Björnsdóttir 2016; | 0.77 | 0.47 | 1.07 | 14.90% |

###
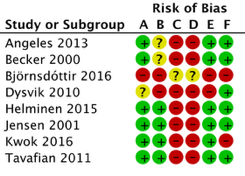


### Intermediate term follow-up

| Study name | Hedges' g | CI Lower limit | CI Upper limit | Weight |
| --- | --- | --- | --- | --- |
| Becker 2000; | 0.48 | 0.07 | 0.90 | 10.16% |
| Cedraschi 2004 | 0.20 | -0.11 | 0.51 | 18.47% |
| Lang 2003 | 0.43 | 0.11 | 0.75 | 17.11% |
| Tavafian 2011 | 0.19 | -0.14 | 0.52 | 16.43% |
| Jensen 2001 (Male) | 0.21 | -0.40 | 0.83 | 4.76% |
| Jensen 2001 (female) | 0.51 | -0.01 | 1.04 | 6.47% |
| Grahn 1998 | 0.09 | -0.17 | 0.35 | 26.60% |

###
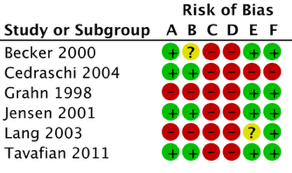


### Long term follow-up

| Study name | Hedges' g | CI Lower limit | CI Upper limit | Weight |
| --- | --- | --- | --- | --- |
| Tavafian 2017 | 0.39 | 0.06 | 0.72 | 30.04% |
| Jensen 2001 (male) | 0.15 | -0.47 | 0.77 | 16.22% |
| Jensen 2001 (female) | 0.88 | 0.34 | 1.43 | 18.87% |
| (NHP) Grahn 2000; | 0.12 | -0.13 | 0.38 | 34.87% |


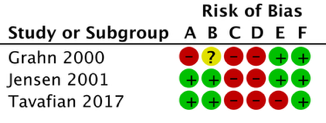


## Social Functioning VS ACGs

### Short term follow-up

| Study name | Hedges' g | CI Lower limit | CI Upper limit | Weight |
| --- | --- | --- | --- | --- |
| Angst 2009 | 0.33 | 0.10 | 0.56 | 13.90% |
| Dufour 2010 | 0.02 | -0.22 | 0.26 | 13.79% |
| Monticone 2012 | -0.32 | -0.77 | 0.12 | 11.56% |
| Monticone 2014 | 1.56 | 0.59 | 2.67 | 6.09% |
| Paolucci 2017 | 0.23 | -0.31 | 0.78 | 10.40% |
| Becker 2000 | -0.10 | -0.52 | 0.31 | 11.90% |
| Jensen 2001 (males) | -0.03 | -0.62 | 0.56 | 9.88% |
| Jensen 2001 (Females) | 0.03 | -0.45 | 0.52 | 11.08% |
| Monticone 2013 | 1.28 | 0.84 | 1.75 | 11.39% |


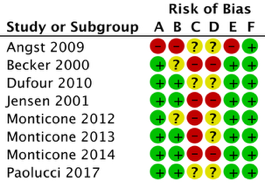


### Intermediate term follow-up

| Study name | Hedges' g | CI Lower limit | CI Upper limit | Weight |
| --- | --- | --- | --- | --- |
| Angst 2009; | -0.16 | -0.44 | 0.11 | 15.88% |
| Dufour 2010 | 0.08 | -0.16 | 0.32 | 16.39% |
| Jensen 2001 (males) | 0.24 | -0.35 | 0.84 | 10.68% |
| Jensen 2001 (females) | 0.38 | -0.10 | 0.88 | 12.27% |
| Monticone 2014 | 1.74 | 0.75 | 2.89 | 5.84% |
| Becker 2000; | 0.28 | -0.13 | 0.70 | 13.48% |
| Jousset 2004; | -0.57 | -1.02 | -0.13 | 13.01% |
| Rooks 2007 | 0.47 | -0.01 | 0.95 | 12.45% |

###
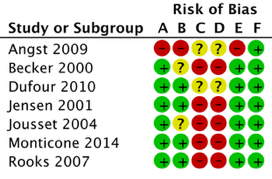


### Long term follow-up

| Study name | Hedges' g | CI Lower limit | CI Upper limit | Weight |
| --- | --- | --- | --- | --- |
| Dufour 2010 | 0.17 | -0.07 | 0.40 | 15.82% |
| Jensen 2001 (males) | 0.23 | -0.36 | 0.83 | 12.62% |
| Jensen 2001 (Females) | 0.19 | -0.29 | 0.68 | 13.68% |
| Monticone 2012; | 0.00 | -0.44 | 0.44 | 14.12% |
| Monticone 2013 | 2.04 | 1.54 | 2.57 | 13.38% |
| Roche-Leboucher 2011 | 0.64 | 0.26 | 1.03 | 14.66% |
| Jensen 2011 | 0.15 | -0.10 | 0.40 | 15.73% |


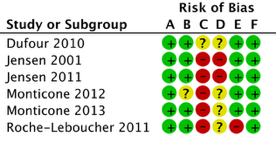


## Vitality VS TAU

### Short term follow-up

| Study name | Hedges' g | CI Lower limit | CI Upper limit | Weight |
| --- | --- | --- | --- | --- |
| Angles 2013 | 0.03 | -0.59 | 0.65 | 9.61% |
| Becker 2000 | 0.22 | -0.20 | 0.63 | 11.64% |
| Dysvik 2010 | 0.45 | 0.08 | 0.82 | 12.06% |
| Helminen 2015 | 0.02 | -0.37 | 0.41 | 11.88% |
| Kwok 2016 | 0.75 | 0.15 | 1.37 | 9.66% |
| Tavafian 2011 | 0.40 | 0.08 | 0.73 | 12.44% |
| Jensen 2001 (male) | 0.39 | -0.23 | 1.02 | 9.57% |
| Jensen 2001 (female) | 0.20 | -0.32 | 0.72 | 10.58% |
| Björnsdóttir 2016 | 1.37 | 1.05 | 1.68 | 12.56% |

###
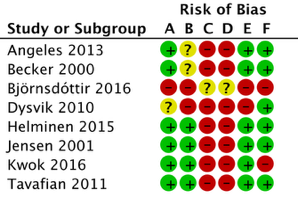


### Intermediate term follow-up

| Study name | Hedges' g | CI Lower limit | CI Upper limit | Weight |
| --- | --- | --- | --- | --- |
| Becker 2000 | 0.76 | 0.34 | 1.19 | 14.54% |
| Lang 2003 | 0.06 | -0.26 | 0.38 | 21.03% |
| Tavafian 2011 | 0.32 | -0.01 | 0.65 | 20.25% |
| Jensen 2001 (male) | 0.48 | -0.13 | 1.12 | 8.24% |
| Jensen 2001 (female) | 0.54 | 0.02 | 1.08 | 10.69% |
| Grahn 1998 | 0.32 | 0.05 | 0.59 | 25.26% |

###
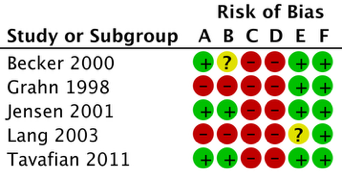


### Long term follow-up

| Study name | Hedges' g | CI Lower limit | CI Upper limit | Weight |
| --- | --- | --- | --- | --- |
| Tavafian 2017 | 0.39 | 0.06 | 0.72 | 30.45% |
| Jensen 2001 (male) | 0.87 | 0.24 | 1.53 | 12.11% |
| Jensen 2001(female) | 0.50 | -0.02 | 1.04 | 16.44% |
| Grahn 2000 | 0.19 | -0.05 | 0.43 | 41.00% |

##
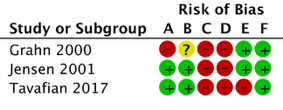


## Vitality VS ACGs

### Short term follow-up

| Study name | Hedges' g | CI Lower limit | CI Upper limit | Weight |
| --- | --- | --- | --- | --- |
| Angst 2009 | 0.25 | 0.03 | 0.48 | 14.05% |
| Dufour 2010 | 0.14 | -0.10 | 0.38 | 13.92% |
| Jensen 2001 (males) | 0.26 | -0.33 | 0.85 | 9.76% |
| Jensen 2001 (females) | 0.14 | -0.35 | 0.62 | 11.03% |
| Monticone 2012 | -0.10 | -0.55 | 0.34 | 11.57% |
| Monticone 2014 | 1.17 | 0.24 | 2.21 | 6.33% |
| Paolucci 2017 | 0.09 | -0.45 | 0.63 | 10.34% |
| Becker 2000 | 0.05 | -0.37 | 0.46 | 11.90% |
| Monticone 2013 | 1.57 | 1.11 | 2.06 | 11.11% |


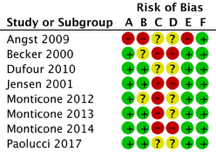


### Intermediate term follow-up

| Study name | Hedges' g | CI Lower limit | CI Upper limit | Weight |
| --- | --- | --- | --- | --- |
| Angst 2009 | 0.03 | -0.24 | 0.30 | 22.21% |
| Dufour 2010 | 0.17 | -0.07 | 0.41 | 24.22% |
| Jensen 2001 (males) | 0.06 | -0.53 | 0.66 | 9.54% |
| Jensen 2001 (females) | 0.23 | -0.25 | 0.72 | 12.36% |
| Monticone 2014 | 1.47 | 0.51 | 2.55 | 4.19% |
| Becker 2000 | 0.51 | 0.09 | 0.93 | 14.70% |
| Rooks 2007 | 0.26 | -0.21 | 0.74 | 12.78% |


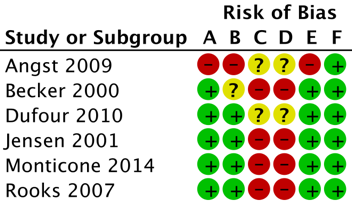


### Long term follow-up

| Study name | Hedges' g | CI Lower limit | CI Upper limit | Weight |
| --- | --- | --- | --- | --- |
| Dufour 2010 | 0.28 | 0.04 | 0.52 | 18.18% |
| Jensen 2001 (males) | 0.13 | -0.46 | 0.72 | 15.15% |
| Jensen 2001 (Females) | 0.00 | -0.48 | 0.48 | 16.19% |
| Monticone 2012 | 0.10 | -0.34 | 0.55 | 16.59% |
| Monticone 2013 | 2.14 | 1.64 | 2.68 | 15.79% |
| Jensen 2011 | 0.10 | -0.15 | 0.35 | 18.10% |


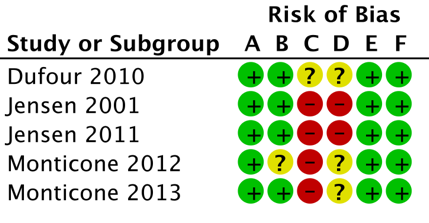

Supplement: Supplementary file 2 — Data S1: ejp70176‐sup‐0002‐DataS1.docx. [file EJP-30-0-s001.docx]
